# Simultaneous speech and gesture decoding for multimodal communication in paralysis

**DOI:** 10.1038/s41593-026-02446-2

**Published:** 2026-09-14

**Authors:** Samantha C. Brosler, Jessie R. Liu, Alexander B. Silva, Irina P. Hallinan, Cady M. Kurtz-Miott, Jonah F. B. Dunkel Wilker, Adelyn Tu-Chan, Karunesh Ganguly, Edward F. Chang

**Affiliations:** 1 Department of Neurological Surgery, University of California, San Francisco, San Francisco, CA USA; 2 Weill Institute for Neurosciences, University of California, San Francisco, San Francisco, CA USA; 3 University of California, Berkeley–University of California, San Francisco, Graduate Program in Bioengineering, Berkeley, CA USA; 4 Department of Neurology, University of California, San Francisco, San Francisco, CA USA

**Keywords:** Neural decoding, Brain-machine interface

## Abstract

Stroke and neurodegenerative diseases can impair speech and nonverbal gestures, limiting natural communication. Brain–computer interfaces (BCIs) aim to restore these functions by translating neural activity into commands for external devices, although prior work has primarily focused on decoding speech or gestures in isolation. Here we show that neural signals recorded with a single high-density electrocorticography implant can support simultaneous decoding of speech and gestures in people with paralysis. We first show that isolated upper-limb and orofacial movements can be reliably decoded among three participants. Using parallel speech and gesture decoders, we then enabled participants to control a personalized virtual avatar by attempting speech and gestures simultaneously or in isolation. Training models on both isolated and simultaneous data improved performance across behavioral contexts. These findings demonstrate that one cortical implant can support multi-effector control and provide a step toward BCIs that enable more natural communication for people with paralysis.

## Abstract (web summary)

Brosler et al. develop a brain–computer interface that simultaneously decodes speech and gestures from a single cortical implant to animate a virtual avatar, providing a step toward more flexible communication for people with paralysis.

## Main

Natural communication is often concurrent and multi-effector, involving distinct motor systems such as the vocal tract and upper limbs. For example, spoken language is frequently accompanied by gestures, and these cospeech gestures contribute to how listeners understand and integrate communicated information^1–3^. Stroke and neurodegenerative diseases, such as amyotrophic lateral sclerosis (ALS), can impair both speech and body movements^4,5^, limiting agency, participation in social activities and substantially reducing quality of life^6,7^. Brain–computer interfaces (BCIs) aim to restore lost motor functions by translating neural activity from the sensorimotor cortex (SMC) into commands for external devices. Recent BCI research has demonstrated the decoding of speech^8–14^ and other nonspeech movements, such as facial expressions^8^, cursor control^15^, manual gestures^16–18^ and walking^19^. However, most BCI research to date has focused on decoding these behaviors in isolation. It therefore remains unclear whether a single BCI implant can reliably decode speech and gestures when they are attempted together, as they often are during natural communication. As BCI systems become more capable, developing multi-effector, multifunctional systems that support both speech and body movements—and that remain robust across isolated and concurrent contexts—is important for future real-world clinical utility and for supporting natural communication.

The organization of the SMC has been shown to support multi-effector decoding of isolated movements at multiple spatial scales. At the population level, movements of the legs, arms, hands and face follow a rough somatotopy^20–22^, enabling movements of these whole-body effectors to be decoded from electrocorticography (ECoG) arrays spanning the precentral and postcentral gyri^8,23–25^. While this general somatotopy exists, it does not necessarily equate with strict neural population boundaries. At finer spatial scales, recent intracortical spiking recordings have shown that, while sites along the precentral gyrus may show a coarse ‘preferred’ body region consistent with the classic somatotopy, neurons at a single site can be broadly tuned and modulated by movements of all four limbs as well as the head and face^26,27^. Given the existence of this partially heterogeneous tuning, speech has been decoded from intracortical spiking activity in the dorsal precentral gyrus—in the canonical ‘hand knob’ region^28^—and cursor control has been demonstrated from the ventral precentral gyrus^15^, an area more commonly associated with speech-motor and orofacial representations^29–31^.

However, a key challenge is developing multi-effector decoding systems that remain accurate when behaviors are attempted concurrently, not only in isolation. Neural patterns observed during isolated behaviors may differ substantially from when behaviors are attempted concurrently, degrading performance of decoders trained only on isolated data. For example, prior work with intracortical spiking recordings showed that, during concurrent movements of both hands, neural tuning changed nonlinearly relative to isolated hand movements, requiring decoder modifications to achieve accurate decoding^18^. Similar challenges have been observed between speech and cursor control, again using intracortical spiking recordings, where simultaneous speech degraded cursor control in the ventral precentral gyrus^15^ and simultaneous cursor control attenuated speech-related signals recorded in the hand knob region^32^. However, it remains unclear how concurrent multi-effector speech and body movements impact decoding when recording from ECoG. Prior work using ECoG or stereoelectroencephalography, which record local field potentials reflecting the combined activity of thousands of neurons, has also shown partially overlapping upper-limb and orofacial activations in SMC^23,33^. These results raise the possibility that ECoG decoders trained only on isolated, single-effector data may not generalize to simultaneous speech and gestures. It is an open question of how multi-effector decoders, whether based on intracortical spiking activity or local field potentials, should be adapted to support reliable decoding when speech and gestures are attempted together. We hypothesized that reliable simultaneous decoding would require training data that includes examples of simultaneous behavior, rather than isolated speech or gestures alone.

Here we used high-density ECoG to record neural activity from three chronically implanted participants with severe paralysis to investigate the feasibility of simultaneous, multi-effector decoding from a single sensorimotor implant. First, we establish that each participant’s array supports reliable decoding across a range of effectors, including upper-limb and speech-related orofacial movements. Building on this, we implemented a real-time system with speech and gesture decoders in parallel to drive a personalized full-body avatar. We show that, while models trained only on isolated behaviors did not automatically generalize to simultaneous speech-and-gesture attempts, incorporating both isolated and simultaneous trials during training improved performance across contexts. Together, these results form a key proof of concept toward a unified BCI that supports multi-effector communication for people with paralysis.

## Results

### Neural representations of multiple body groups for driving a multi-effector BCI

**Fig. 1:**
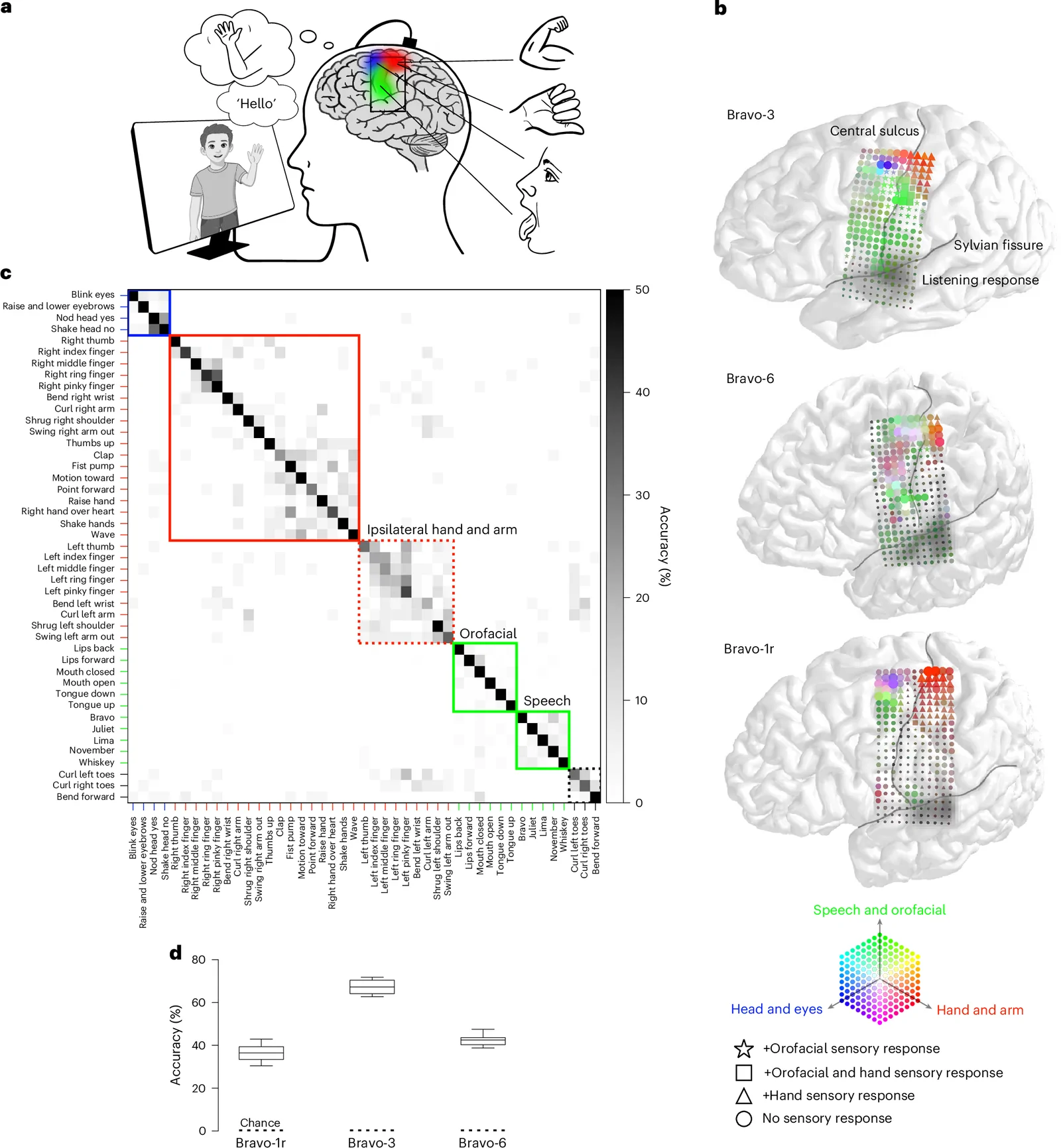
A single ECoG array captures sensorimotor neural representations to drive a multi-effector BCI. **a**, Schematic of a single ECoG array sampling multi-effector representations that can be used for BCI control. **b**, Maps displaying the relative sensorimotor tuning of electrodes in each participant’s ECoG array between three movement categories—head and/or eyes in blue, hand and/or arm in red and speech and/or orofacial in green. Electrodes are colored according to the three-dimensional RGB colormap based on the movement categories. An electrode with strong and relatively equal tuning to all three categories would appear white, an electrode with strong and relatively equal tuning to hand/arm and speech/orofacial but no head/eyes tuning would appear yellow, while an electrode with strong speech/orofacial tuning, weaker hand/arm tuning and no head/eyes tuning would appear chartreuse and so on. The relative size of electrodes is scaled with the magnitude of neural response. The marker used indicates whether the electrode had orofacial or hand sensory responses, both or neither. The central sulcus and Sylvian fissure are annotated with black lines. A density map of listening responses is shown in gray behind the grid. **c**, Confusion matrix for the classification of multi-effector movements for Bravo-3. Blue, red and green ticks and boxes correspond to the effector groups in **b**, with hand/arm further broken out into which are ipsilateral and speech/orofacial broken out into orofacial movements versus speech. Movements in the dotted boxes are not a part of the maps in **b**. The black ticks and box correspond to other movements, including torso, leg and foot movements. **d**, Average accuracy of multi-effector classification for each participant across *n* = 10 cross-validation folds. Chance is 2.22% for Bravo-3 and Bravo-6 and 2.33% for Bravo-1r. Box plots show the median (centerline) and the 25th and 75th percentiles (box limits), and the whiskers extend to the minimum and maximum values within 1.5× interquartile range.

BCIs that aim to support communication through simultaneous speech and nonspeech gestures must sample multi-effector motor representations^15,23,26,27^, including representations of hand and orofacial movements (Fig. 1a). Therefore, we first sought to verify that single ECoG arrays placed over the SMC could robustly sample both orofacial and body representations in several participants with varying degrees of paralysis. We hypothesized that robust sampling of both orofacial and body movement representations from a single ECoG array would be possible given prior work demonstrating that the broad somatotopic organization of the SMC^20^ persists even after paralysis^8,34,35^.

We recorded neural activity while three participants performed a battery of movements involving different effectors across the body. Bravo-1r and Bravo-3 had severe vocal-tract and limb paralysis, while Bravo-6 had severe vocal-tract paralysis and moderate limb paralysis. Participants’ attempt strategies differed based on paralysis severity and participant preference (for detailed descriptions of each participant’s behavior, see ‘Participants’). Bravo-1r silently attempted speech and minimally attempted other movements, Bravo-3 silently attempted speech and attempted other movements and Bravo-6 attempted speech with vocalization (overt) and imagined other body movements with no behavioral output (covert). Because Bravo-1r and Bravo-3 had severe bodily paralysis, body movement attempts often did not result in observable behavior.

By computing the maximum evoked high-gamma activity (HGA; 70–150 Hz) per movement type, we assessed the preference of each electrode for movements that involved the orofacial speech articulators, hands and head/eyes (excluding ipsilateral movements and those of the trunk or legs; ‘Multi-effector sensorimotor maps’). Across our participants, we found that electrodes with preferences for these articulator groups were arranged roughly somatotopically along the SMC (Fig. 1b), in line with our prior work^8^. Electrodes that preferred hand movements were generally located on the dorsal–posterior aspect of the SMC covered by our grids. Electrodes that preferred head/eye movements were located on the dorsal–anterior aspect of the grid, partially overlapping with putative frontal eye fields^36^. Finally, electrodes that preferred orofacial speech movements were localized ventrally to those other electrode groups, extending toward the Sylvian fissure. While this coarse organization was present in all three participants, the degree of segregation varied across individuals. In particular, Bravo-6 showed more intermixed preferences across the grid, with hand-preferring and arm-preferring electrodes extending more ventrally than in Bravo-1r and Bravo-3 (Fig. 1b). This interparticipant variability may reflect differences in array coverage and/or clinical etiology (Bravo-6’s motor impairments were caused by ALS, whereas Bravo-1r and Bravo-3 had brainstem stroke), consistent with prior reports that sensorimotor organization and recruitment can vary across individuals^22^. Overall, this somatotopic organization aligns with prior work in healthy individuals^20,29,37^ and those with paralysis^8,34,35^.

As our coverage also extended to somatosensory cortex, we then tested whether we could sample tactile sensory representations. Indeed, we found a subset of electrodes, largely confined to the postcentral gyrus, that had activity evoked by tactile sensation (Fig. 1b). These electrodes were also specific for either the hands or orofacial articulators, arranged somatotopically and aligning with prior work^20,35^. Finally, our ECoG arrays also extended to the superior temporal gyrus where we observed expected neural responses to listening to various sentences (Fig. 1b).

While access to these representations is a critical first step, activity must also be decodable across different movements to drive a multi-effector BCI^15,23,26,27^. To test this, we fit a single decoding model across all these movements, including ipsilateral movements and a few additional movements involving the trunk and legs (‘Multi-effector classification’). Notably, many of these movements were above chance and readily distinguishable from each other (Fig. 1c,d and Supplementary Fig. 1), with the exception of left hand movements and leg movements, aligning with the overall coverage of the ECoG arrays. Thus, a single ECoG array can provide access to a host of movement representations that could drive multi-effector BCIs.

### Decoder performance depends on behavioral context

**Fig. 2:**
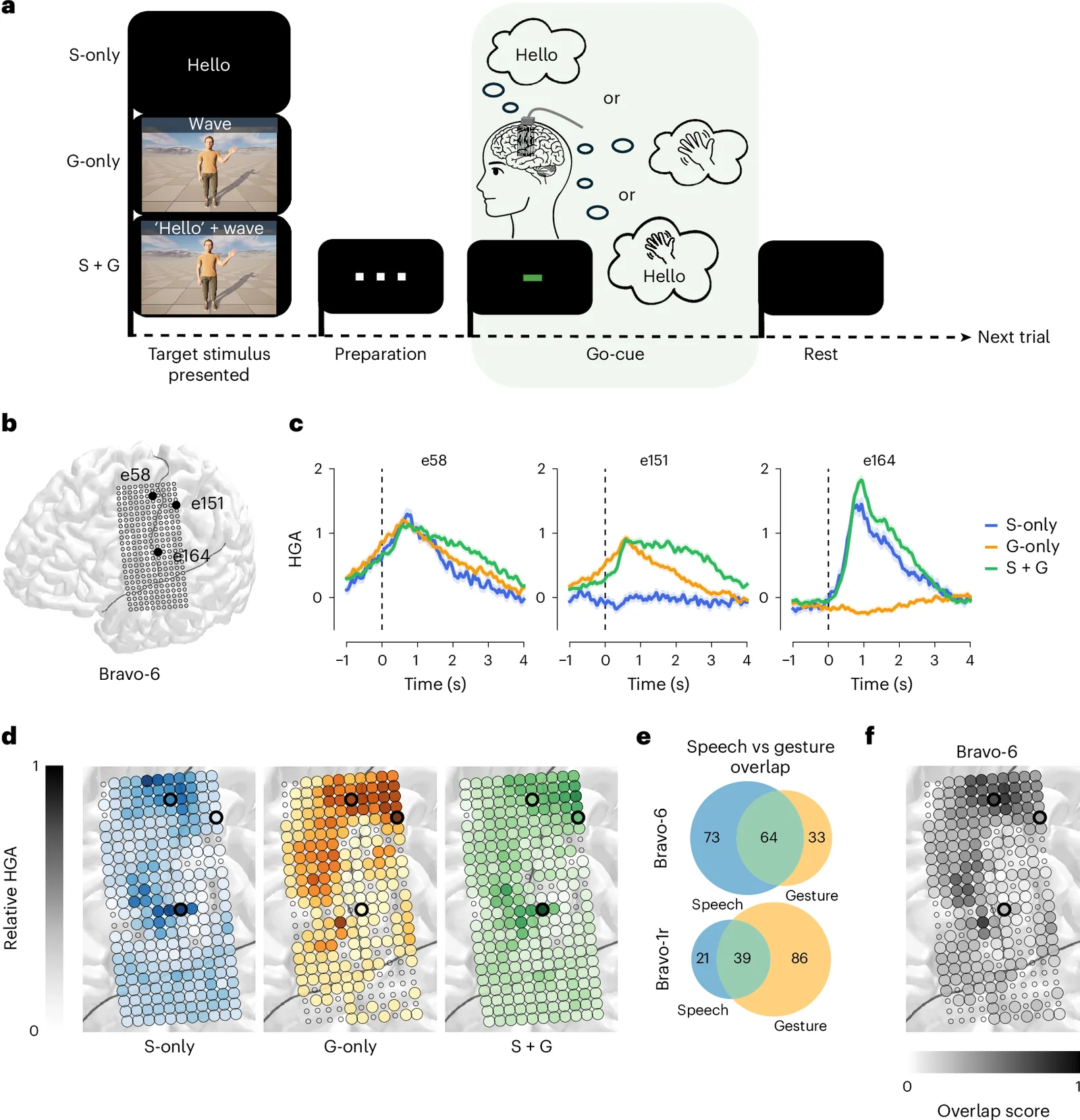
Attempted speech and gestures recruit partially overlapping neural populations. **a**, Trial structure for three task paradigms—S-only (text stimulus), G-only (animated avatar stimulus) and simultaneous S + G (text and animated avatar stimulus). Stimulus presentation is followed by a countdown to a go-cue, when the participant is instructed to attempt the target (see Methods for behavioral descriptions). **b**, Highlighted electrodes (e58, e151 and e164) overlaid on Bravo-6’s brain. **c**, HGA (mean ± s.e.m.) for the same three example electrodes during S-only trials (blue), G-only trials (orange) and S + G trials (green) aligned to the go-cue. **d**, Spatial maps of relative HGA during S-only, G-only and S + G trials for Bravo-6. Circled electrodes correspond to those labeled in **b** and plotted in **c**. **e**, Venn diagrams of the overlap between electrodes with significant neural activity during S-only trials and G-only trials (Bravo-6, top; Bravo-1r, bottom). **f**, The overlap map highlights electrodes significantly active during both speech and gesture conditions for Bravo-6. Higher overlap scores indicate electrodes with stronger relative HGA modulation during both behaviors, whereas lower overlap scores indicate weaker modulation in one or both conditions. Nonsignificant electrodes are shown as empty outlined circles.

To investigate multi-effector decoding for communication, we focused on speech and communicative gestures and collected data in three behavioral contexts—speech-only (S-only), gesture-only (G-only) and simultaneous speech-plus-gesture (S + G) trials in which two participants were instructed to attempt a target phrase and gesture concurrently (Fig. 2a and ‘Copy task’). At the go-cue, the stimulus was replaced by a green line instructing the participant to attempt the cued behavior. Each trial ends with an intertrial rest period before the next trial. Of note, Bravo-6 and Bravo-1r used slightly different strategies to attempt speech and gestures. Bravo-1r silently attempted speech and minimally attempted gestures, whereas Bravo-6 overtly attempted speech and imagined gestures (‘Participants’). For Bravo-1r, we used a vocabulary of 4 gestures, 5 speech utterances and 12 S + G combinations. For Bravo-6, we used a vocabulary of 10 gestures, 10 speech utterances and 100 S + G combinations. Across participants, there was robust HGA when attempting speech and gestures at multiple electrodes spanning the grid (Fig. 2b–d and Supplementary Figs. 2 and 3), with some electrodes showing selective activity only during speech, others only during gestures and others during both speech and gestures (Fig. 2c). The electrodes significantly active during S-only and G-only also showed significant activations during S + G trials. Across behaviors, the strongest modulation was primarily in the precentral gyrus for all participants (Fig. 2d and Supplementary Fig. 4). As expected, speech-evoked activity was concentrated more ventrally in the vPrCG, whereas gesture-evoked activity was localized more dorsally near putative hand and arm representations (Fig. 2d and Supplementary Fig. 4). Notably, speech and gesture responses were not fully segregated. A subset of electrodes, particularly in precentral gyrus, were modulated during both behaviors (Fig. 2e). We further quantified the overlap between putative speech-related and gesture-related activity at each electrode using the geometric mean of the maximum HGA observed during S-only and G-only trials^33^ (‘Overlap score’). This metric yields a high score for electrodes with strong activation during both S-only and G-only trials and a low score for electrodes with weak activation during either behavior. In both participants, electrodes with high overlap scores were localized to the precentral gyrus, spanning a broader spatial extent in Bravo-6 and concentrated more focally in mPrCG in Bravo-1r (Fig. 2f and Supplementary Fig. 4d). This partial overlap suggests that models trained only on neural activity during isolated behaviors may not fully capture the population activity recruited when they are attempted concurrently, motivating an explicit test of generalization to simultaneous contexts.

We first evaluated how well models trained on isolated speech or gesture data generalized to simultaneously attempted speech and gestures. Separate neural network models were trained on neural activity recorded during S-only and G-only blocks, respectively (‘Speech and gesture modeling’). Each model was then tested on data from S + G trials, in which the participant was instructed to attempt a target phrase while simultaneously imagining a communicative gesture. We varied the amount of training data by training models on increasing amounts of isolated data and compared their performance to models trained exclusively on S + G data, evaluating all models on held-out S + G trials (Supplementary Fig. 5). For both participants, S + G decoding accuracy increased with additional training data across model types (Supplementary Fig. 5a,b). At the largest shared training size, S + G-trained models achieved higher accuracy on S + G trials than models trained only on isolated data, whereas models trained only on isolated data achieved higher accuracy on S-only or G-only trials than S + G-trained models (Supplementary Fig. 5; two-sided Mann–Whitney *U* tests, all *P* < 0.002). Together, these findings show that decoder performance is context dependent, with models performing best when training and testing are matched to the same behavioral context.

### Training on a combination of isolated and simultaneous behavior improves model generalization

**Fig. 3:**
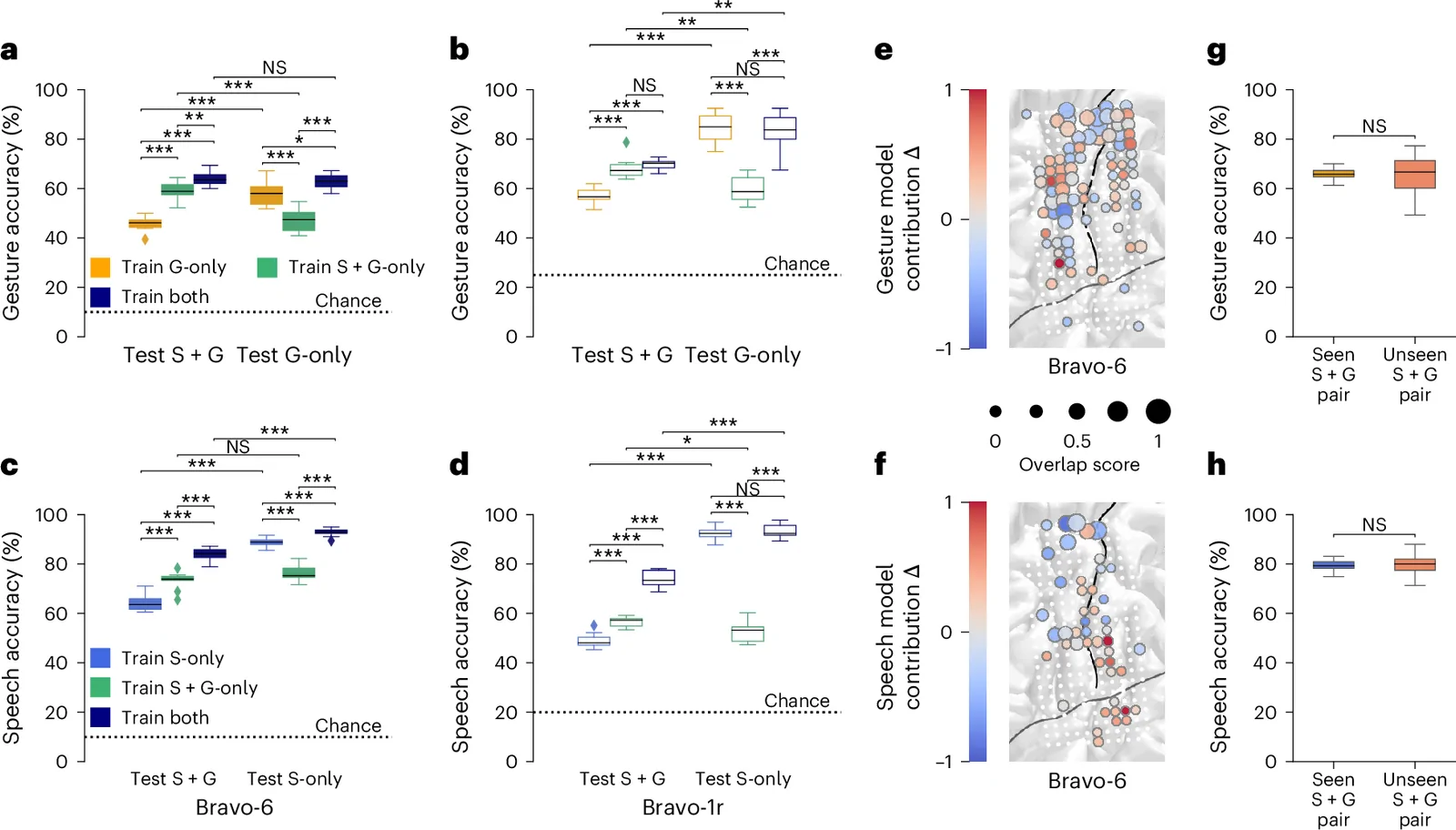
Simultaneous speech and gestures can be decoded from cortical activity. **a**, Gesture decoding accuracy for Bravo-6 on simultaneous S + G and isolated G-only test sets for models trained on G-only, S + G-only or both datasets (ten gestures; chance = 10%). **b**, Same as **a** for Bravo-1r (four gestures; chance = 25%). **c**, Speech decoding accuracy for Bravo-6 on S + G and isolated S-only test sets for models trained on S-only, S + G-only or both datasets (ten phrases; chance = 10%). **d**, Same as **c** for Bravo-1r (five phrases; chance = 20%). In **a**–**d**, each box plot contains *n* = 10 ensemble decoding accuracies per training/test condition. Pairwise comparisons used two-sided Mann–Whitney U tests with nine-way FDR correction (NS, *P* ≥ 0.05; *P < 0.05; **P < 0.01; ***P < 0.001). Exact *P* values are provided in Supplementary Table 1. **e**, Change in electrode contribution scores between Bravo-6 gesture models trained on isolated versus S + G data; warmer/cooler colors indicate increased/decreased electrode contribution after S + G training. Electrode size is scaled by overlap score (Fig. 2f). **f**, Same as **e**, but for the Bravo-6 speech model. **g**, Leave-one-phrase-out analysis for the Bravo-6 gesture decoder. Accuracy was evaluated on S + G trials containing phrases that were either included during training (‘seen’) or withheld from S + G training (‘unseen’). **h**, Leave-one-gesture-out analysis for the Bravo-6 speech decoder. Accuracy was evaluated on S + G trials containing gestures that were either included during training (‘seen’) or withheld from S + G training (‘unseen’). Accuracy was not significantly different between conditions (two-sided Mann–Whitney *U* test, *P* = 0.29 and *P* = 0.35, respectively). In **g** and **h**, each box contains *n* = 150 ensemble decoding accuracies, corresponding to ten leave-one-out conditions evaluated across 15 train-test splits from threefold cross-validation with five repeats. All box plots show the median (centerline) and the 25th and 75th percentiles (box limits), and the whiskers extend to the minimum and maximum values within 1.5× interquartile range.

Because decoder performance depended on training context, we then asked whether combining isolated and simultaneous datasets could enhance generalization across behavioral contexts. We trained hybrid models using an equal split of isolated behavior and S + G trials (‘Speech and gesture modeling’). These hybrid models maintained high accuracy across both isolated and simultaneous test conditions, outperforming models that were, in contrast, trained on only one type of data (Fig. 3a–d). To probe a potential contributor to this context dependence, we compared electrode contribution maps from models trained on isolated versus simultaneous data (Fig. 3e,f and ‘Electrode contributions’). We calculated the difference in electrode contributions for models trained exclusively on simultaneous versus isolated data and found training-dependent changes in both speech and gesture models. In Bravo-6, the largest decreases in electrode contributions for both speech and gesture models occurred at electrodes that also had high overlap between speech-related and gesture-related activity. The largest increases typically occurred at electrodes with lower overlap. In Bravo-1r, the largest changes in electrode contributions were similarly concentrated at regions of high speech–gesture overlap (Supplementary Fig. 6). Notably, these shifts did not suggest that overlapping electrodes were always uninformative for decoding, but that training on simultaneous behavior changed how those electrodes were weighted. Indeed, many electrodes that were downweighted still had significant electrode contributions. Simultaneous training may reduce model reliance on shared electrodes and increase reliance on more separable, modality-specific signals, which could contribute to improved decoding in the simultaneous context. Further analyses of embeddings output by the final gated-recurrent unit (GRU) layer showed that training on both isolated and simultaneous behaviors brought the centers of the embedding distributions for the same phrase or gesture closer together across contexts (Supplementary Figs. 7a–d and 8a–d). However, differences in the geometry of the isolated and simultaneous embedding distributions remained (Supplementary Figs. 7e,f and 8e,f). These findings indicate that simultaneous trials contain representational structure not fully captured by isolated data alone, which may explain the improved generalization of models trained on both isolated and simultaneous data across behavioral contexts. Practically, these results demonstrate that multi-effector BCIs intended for use during both isolated and simultaneous behaviors should be context inclusive. That is, training should include examples of both behavioral contexts.

To test whether models trained on both isolated and simultaneous data generalize to speech–gesture pairings not seen together during training, rather than memorizing specific combinations, we performed a leave-one-out analysis. For the speech decoder, all S + G trials containing one gesture were withheld from S + G training data; for the gesture decoder, all S + G trials containing one phrase were withheld from S + G training data, while models were trained on all remaining S + G combinations (‘Speech and gesture modeling’). For example, when the phrase ‘hello’ was withheld from the gesture decoder, the model was trained on all 10 isolated gesture trials and the 90 remaining S + G combinations, but no simultaneous trials that paired ‘hello’ with a gesture. Thus, the individual speech classes were still seen by the speech model in isolation and the individual gesture classes were still seen by the gesture model in isolation, whereas the specific held-out simultaneous pairing was not. We then compared decoding accuracy on S + G trials containing phrases or gestures included during S + G training (‘seen’) versus those withheld (‘unseen’; Fig. 3g,h). This analysis was performed only for Bravo-6 due to utterance set limitations in Bravo-1r (‘Copy task’). For both models, median performance on seen and unseen S + G trials did not differ significantly. For the gesture model, median accuracy was 65.8% (95% confidence interval (CI) = 65.6, 66.2) on seen pairs compared to 66.7% (95% CI = 64.7, 68.7) on unseen pairs (*P* = 0.29, two-sided Mann–Whitney *U* test; Fig. 3g). For the speech model, median accuracy was 79.3% (95% CI = 78.9, 79.8) on seen pairs compared to 80.0% (95% CI = 79.3, 80.7) on unseen pairs (*P* = 0.35, two-sided Mann–Whitney *U* test; Fig. 3h). We further tested whether generalization depended on whether the speech–gesture pair was categorized as natural or unnatural in conversation. Within both the seen and unseen conditions, neither speech nor gesture decoding accuracy differed significantly between natural and unnatural pairs (Supplementary Fig. 9). These results indicate that decoder performance in the simultaneous context did not depend strongly on whether a specific phrase–gesture pairing had been present during S + G training or the naturalness of that combination in conversation. Together, this suggests that, although training on simultaneous behavior improves performance in simultaneous contexts, the models may not need to observe every possible speech–gesture combination to decode new simultaneous pairings. This finding is encouraging for scaling to larger vocabularies, where exhaustively sampling all possible speech–gesture combinations would be impractical.

Finally, we examined longitudinal stability by freezing context-inclusive speech and gesture decoders trained through successive cutoff dates and evaluating them without recalibration on later S + G sessions. Accuracy remained above chance across days to months, although stability varied across participants and modalities, and incorporating more recent training data generally improved performance on subsequent sessions (Supplementary Figs. 10 and 11).

### Cross-modality training reduces false positive rate (FPR) and false negative rate (FNR)

**Fig. 4:**
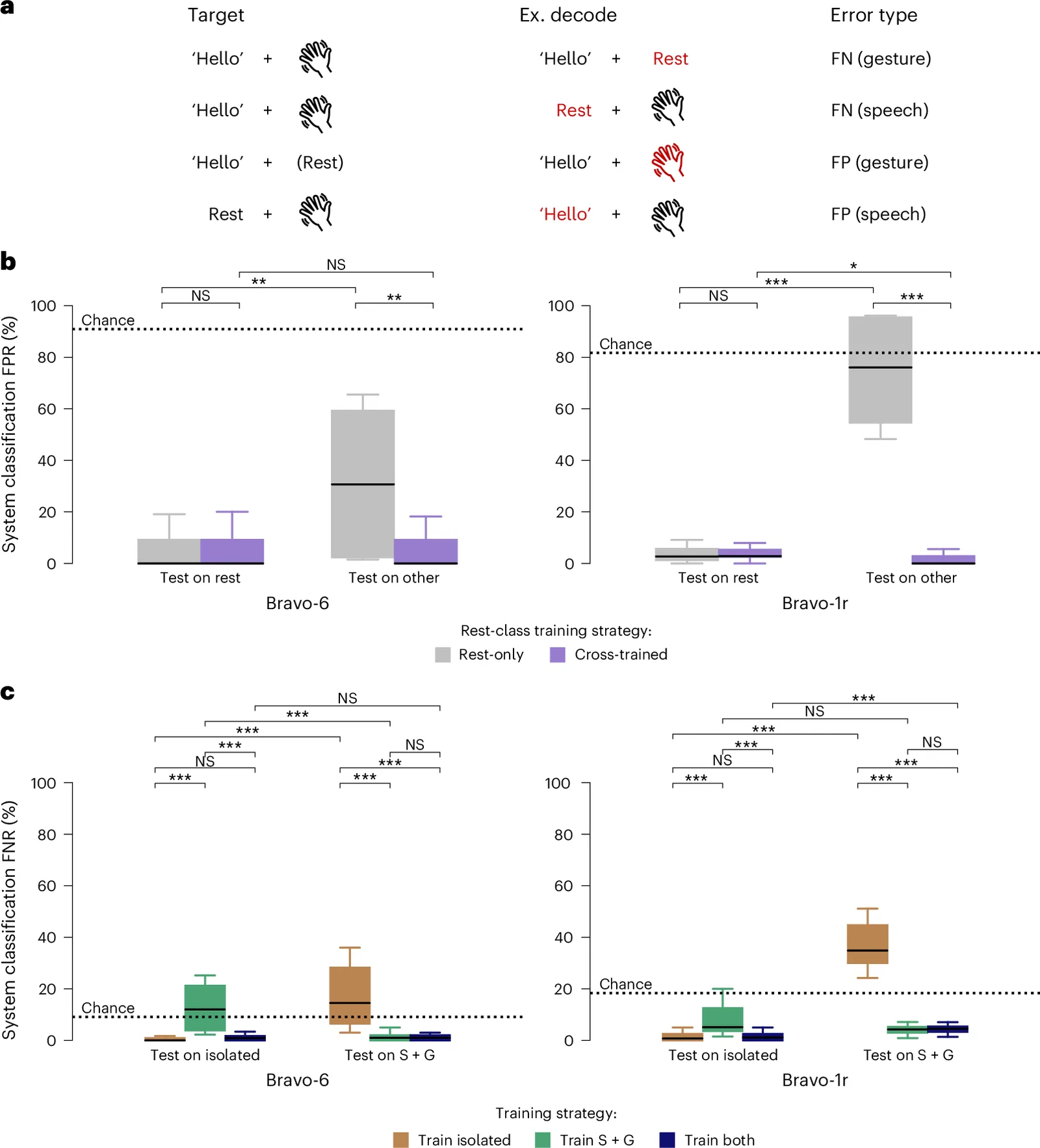
Cross-modality and context-inclusive training reduces error rates. **a**, Schematic of error types when speech and gesture decoders are run in parallel. FPs occur when a decoder outputs an active class instead of rest during true rest or opposite-modality trials; FNs occur when a decoder outputs rest during attempted speech or gesture. **b**, System classification FPR for models trained with a rest class composed of true-rest data only (rest-only) or a balanced combination of true rest and opposite-modality data labeled as rest (cross-trained). Cross-modality training reduced FPR during opposite-modality testing in both participants. **c**, System classification FNR for models trained on isolated behaviors only (train isolated), simultaneous S + G trials only (train S + G) or both (train both). Dotted lines indicate chance level. Each box contains *n* = 20 decoder-evaluation values (10 speech-decoder and 10 gesture-decoder test splits) for one participant. Box plots in **b** and **c** show the median (centerline) and the 25th and 75th percentiles (box limits), and the whiskers extend to the minimum and maximum values within 1.5× interquartile range. Pairwise comparisons were performed using two-sided Mann–Whitney U tests with Benjamini–Hochberg FDR correction across four comparisons in **b** and nine comparisons in **c**, separately within each participant. Exact *P* values for **b** and **c** are provided in Supplementary Table 2 (NS, *P* ≥ 0.05; *P < 0.05; **P < 0.01; ***P < 0.001).

To use speech and gesture decoders in parallel, each decoder must remain inactive when the participant is performing the other modality; the speech decoder should output rest during G-only trials, and the gesture decoder should output rest during S-only trials. We therefore quantified FPR during both true rest and the opposite modality, where opposite-modality FPR was defined as the proportion of opposite-modality trials incorrectly classified as a nonrest output rather than rest (Fig. 4a and ‘FP and FN calculations’). Figure 4 summarizes system-level FPR and FNR across the parallel speech and gesture decoders, and decoder-specific error rates are shown separately in Supplementary Fig. 12.

Speech and gesture models were initially trained to predict rest, in addition to their speech or gesture classes, by incorporating true-rest trials during training. When tested on true-rest data, pooled system-level FPR was low for both participants, with median FPRs of 0.0% (95% CI = 0.0, 9.1) for Bravo-6 and 2.7% (95% CI = 1.4, 5.4) for Bravo-1r (Fig. 4b). However, FPR was much higher when tested on the opposite modality, with median FPRs of 30.6% (95% CI = 2.6, 58.6) for Bravo-6 and 76.0% (95% CI = 54.8, 95.3) for Bravo-1r. Thus, models trained with only true-rest examples in the rest class often produced false activations when the participant attempted the other modality.

To reduce these false activations, we implemented cross-modality training, in which the rest class was trained using a balanced combination of true-rest data and data from the opposite modality (‘Cross-modality training’). That is, the gesture-model rest class was trained with both true-rest trials and from S-only data labeled as ‘rest’, and vice versa, for the speech model. Cross-modality training significantly reduced opposite-modality FPR to 0.0% (Bravo-6—95% CI = 0.0, 9.1; Bravo-1r—95% CI = 0.0, 2.7) for both participants, while maintaining low FPR on true-rest trials (Bravo-6—0.0% (95% CI = 0.0, 9.1%); Bravo-1r—2.8% (95% CI = 2.7, 4.1%); Fig. 4b). This reduction was observed for both the gesture and speech models (Supplementary Fig. 12a,b).

We then evaluated FNR, defined as the proportion of true attempted speech or gesture trials incorrectly classified as rest. Models trained only on isolated behaviors frequently misclassified simultaneous S + G trials as rest (Fig. 4c). Specifically, isolated-trained models had median FNRs of 0.0% (95% CI = 0.0, 0.8) for Bravo-6 and 0.8% (95% CI = 0.4, 2.4) for Bravo-1r on isolated trials, but FNRs increased to 14.5% (95% CI = 7.0, 27.5) and 34.9% (95% CI = 30.6, 44.3), respectively, on S + G trials (Fig. 4c). On the other hand, models trained exclusively on S + G data often misclassified isolated trials as rest, with median isolated-test FNRs of 12.0% (95% CI = 4.4, 20.2) for Bravo-6 and 5.2% (95% CI = 3.8, 11.2) for Bravo-1r. In contrast, models trained on both isolated and simultaneous behaviors showed low FNRs across test contexts in both participants, with median FNRs of 0.8% (95% CI = 0.0, 1.7) for Bravo-6 and 1.1% (95% CI = 0.0, 2.5) for Bravo-1r on isolated trials and 1.0% (95% CI = 0.0, 1.5) for Bravo-6 and 4.5% (95% CI = 3.5, 5.3) for Bravo-1r on S + G trials. Together, these results show that cross-modality and context-inclusive training reduced FPRs and FNRs, supporting the reliability of parallel speech-and-gesture decoding across behavioral contexts.

Training these models on the full available isolated and simultaneous datasets and incorporating cross-modality training, mean offline simultaneous decoding accuracy was 68.8% (95% CI = 66.4, 71.1) for gestures (ten gestures plus rest, chance = 9.1%) and 77.5% (95% CI = 75.1, 79.8) for speech (ten phrases plus rest, chance = 9.1%) for Bravo-6. For Bravo-1r, mean offline simultaneous accuracy was 88.3% (95% CI = 86.8, 89.6) for gestures (four gestures plus rest, chance = 20%) and 84.0% (95% CI = 82.8, 85.1) for speech (five phrases plus rest, chance = 16.7%; Supplementary Fig. 13).

### Decoded speech and gesture outputs can control a virtual avatar in real time

**Fig. 5:**
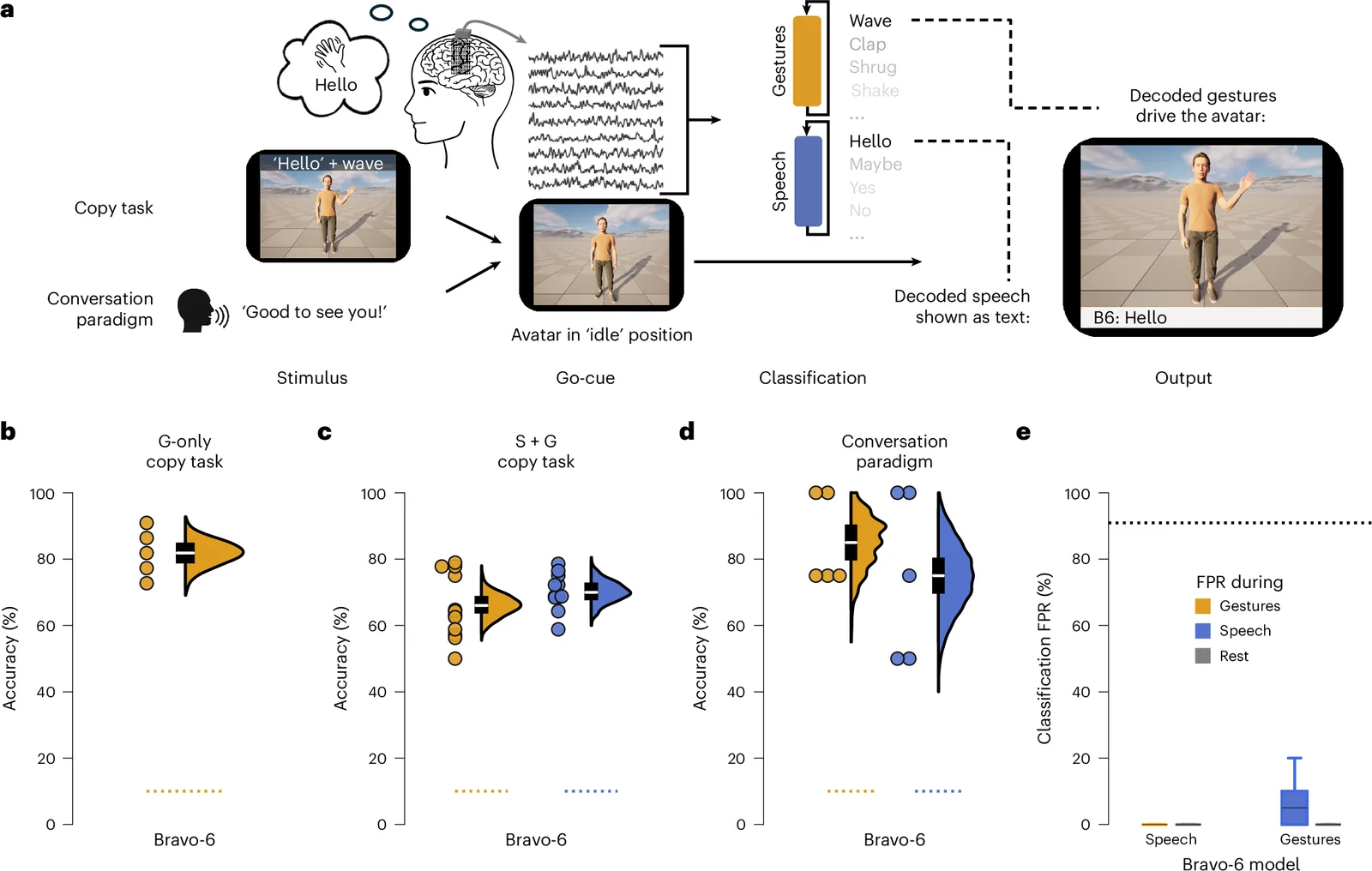
ECoG BCI control of a personalized full-body avatar. **a**, Schematic of the two real-time, closed-loop task paradigms. In both the copy task and conversation paradigm, Bravo-6 attempted speech and/or gestures, from a vocabulary of ten gestures and ten speech utterances, after a go-cue while neural activity was streamed to speech and gesture decoders in parallel. Decoded speech was displayed as text and decoded gestures were animated online by a personalized virtual avatar. **b**, Real-time gesture decoding accuracy in an online G-only copy task for Bravo-6. Dots denote accuracy for each real-time block, half-violin plots show the bootstrapped distribution of these accuracies and dotted lines indicate chance level. **c**, Real-time speech and gesture decoding accuracy in an online simultaneous speech-and-gesture copy task for Bravo-6 (ten gestures and ten phrases, excluding rest trials). **d**, Similar to **c**, but for the cued conversation paradigm for Bravo-6 across proof-of-concept conversational prompts. **e**, FPR of each decoder during nontarget behavior, including rest and isolated attempts of the opposite modality. Dotted line indicates the chance. Box plots in **b**–**e** show the median (centerline) and the 25th and 75th percentiles (box limits), and the whiskers extend to the minimum and maximum values within 1.5× interquartile range.

To support more expressive communication than text alone, we mapped decoded speech and gestures to control a personalized full-body avatar in real time for both Bravo-1r and Bravo-6. Two types of closed-loop tasks were collected with the avatar interface (Fig. 5a, ‘Copy task’ and ‘Conversation paradigm’). In the copy task, the participants were cued to attempt specific gestures in isolation or simultaneously with an attempted speech phrase (Supplementary Videos 1 and 2). In the cued conversation paradigm, the participant responded to spoken conversational prompts (Supplementary Videos 3 and 4). In both paradigms, decoded gestures drove the movement of the avatar and decoded speech was displayed as text at the bottom of the screen (‘Real-time avatar animation’).

In the G-only copy task, real-time decoding accuracy was 81.8% (95% CI = 74.5, 89.1) for Bravo-6 and 61.7% (95% CI = 55.3, 68.2) for Bravo-1r across the same set of gestures plus rest (chance = 9.1%; Fig. 5b, Supplementary Video 1 and ‘Real-time evaluation’). In the speech-and-gesture copy task, accuracy was 66.0% (95% CI = 59.5, 72.5) for gestures and 70.0% (95% CI = 63.5, 76.0) for speech across ten gestures and ten phrases for Bravo-6 (chance = 9.1% for each decoder; Fig. 5c and Supplementary Video 2). For Bravo-1r, simultaneous speech-and-gesture accuracies were evaluated only in offline analyses (Supplementary Fig. 13). The conversation paradigm was run for a limited number of trials and speech–gesture pairs in both participants to provide a proof of concept for parallel decoding of speech and gestures in a conversational setting. The conversation paradigm included S-only, G-only and simultaneous speech-and-gesture trials to quantify performance. Across five blocks of the conversation paradigm, average accuracy for Bravo-6 was 85.0% (95% CI = 70.0, 100.0) for the gesture decoder and 75.0% (95% CI = 55.0, 90.0) for the speech decoder (chance = 9.1% for each decoder; Fig. 5d and Supplementary Video 3). Median accuracy for Bravo-1r was 100.0% for both gesture and speech decoders across three blocks (chance = 16.7% for speech, chance = 20.0% for gestures; Supplementary Video 4). These accuracies were consistent with performance in the nonconversational copy tasks, showing that speech-and-gesture decoding remained robust during more naturalistic conversational prompting. FPRs were low across real-time tasks (average speech FPR = 0.0% and average gesture FPR = 2.5% (95% CI = 0.0, 7.5) for Bravo-6; Fig. 5e). As a qualitative extension of these closed-loop experiments, we also demonstrate that the same system can support a fully virtual conversation between the participant’s BCI-controlled avatar and a second avatar controlled by an experimenter through keyboard-entered speech and gesture commands. In this demonstration, speech outputs appeared as text in a shared chatbox, while gestures animated the corresponding avatars and were logged as italicized text in the chat history, allowing the participant and experimenter to interact virtually using speech alone, gestures alone, or speech and gestures simultaneously (Supplementary Video 5).

## Discussion

Here we investigated the feasibility of a multi-effector speech-and-gesture BCI using a single ECoG implant. Different effectors recruit distinct, yet partially overlapping, spatial patterns across the SMC. By spanning broad sensorimotor territory, we show that a single ECoG grid can support decoding of a variety of upper-body, orofacial and articulatory movements in three participants. We demonstrate a proof-of-concept, real-time interface that decodes simultaneous speech and upper-body gestures to animate a personalized, full-body avatar for expressive interaction. Together, these findings support the feasibility of simultaneous, multi-effector decoding from a single implant as a step toward more flexible, expressive communication for people with severe bodily and vocal-tract paralysis.

BCIs have strong potential to restore both communication and motor function for people with paralysis^38–40^. In practice, BCI systems will need to support naturalistic, multi-effector behavior and remain reliable when those behaviors occur in isolation or concurrently. Although prior work has established accurate decoding of individual modalities from the SMC (for example, speech^8–14^, cursor control^15,41^ and gestures^16^), most demonstrations decode these functions in isolation. Comparatively less work has examined concurrent multi-effector decoding in which speech is decoded in parallel with another motor behavior. The few studies that have tested this scenario suggest that concurrent behaviors can interfere with one another, indicating that simultaneous multi-effector control is a general challenge for BCIs rather than one specific to our recording modality or effector pairing. For example, in two studies using intracortical spiking recordings, simultaneous speech was found to degrade cursor control driven by neural activity from the ventral precentral gyrus, while simultaneous cursor control attenuated speech-related activity in the dorsal precentral gyrus^15,32^. Whether similar challenges arise for decoding neural activity recorded using ECoG—and how best to mitigate them—has remained largely untested. We therefore asked the following questions: (1) how well do ECoG decoders generalize when speech is decoded in parallel with another motor behavior, and vice versa? And (2) what training strategies support reliable decoding during concurrent behavior? We addressed these questions by evaluating speech and gesture decoders both when speech and gestures were attempted in isolation and when they were attempted simultaneously.

We found that simultaneous speech-and-gesture decoding with ECoG, like previous studies with intracortical spiking recordings, was also nontrivial—speech and gesture models trained exclusively on isolated attempts did not fully generalize to simultaneous speech-and-gesture attempts. Incorporating simultaneous trials during model training, alongside isolated trials, improved generalization across behavioral contexts, enabling more reliable decoding during isolated or concurrent attempts. In addition, including opposite-modality-only trials during training gave each model more context about conditions in which the target behavior was absent, reducing false activations when the participant intended only speech or only gestures and improving performance when the two decoders were run in parallel. Together, these training strategies enabled proof-of-concept real-time simultaneous speech-and-gesture decoding in participants with severe vocal tract and bodily paralysis. Building on our prior work demonstrating a BCI-controlled facial avatar^8^, we implemented a full-body avatar output interface to render parallel speech-and-gesture decoding and to capture the contribution of cospeech gestures to natural communication. Even within the restricted proof-of-concept vocabulary used here, decoded gestures rendered by the avatar could serve as standalone communicative responses or, when paired with speech, add emphasis or expressivity.

Multiple factors may necessitate providing models with simultaneous speech-and-gesture context during training. One likely challenge is that effector representations in the precentral gyrus are not perfectly segregated^20,42–45^. Indeed, we observed overlapping HGA modulation in the precentral gyrus during speech and upper-limb gestures across participants (Fig. 2e,f and Supplementary Fig. 4d). Notably, this partial overlap does not imply that speech and gesture signals during concurrent activity are indistinguishable. Alongside electrodes that showed overlapping activity, we also observed electrodes that were only active during speech or gestures, and not both, that contributed to decoding movements of that group. Simultaneous speech-and-gesture training generally shifted model reliance toward electrodes with lower overlap and away from electrodes with higher overlap, suggesting that the models learned to place greater weight on more separable task-relevant signals (Fig. 3e,f). At the same time, overlapping electrodes were not uniformly detrimental, as some electrodes with strong overlap still contributed to model predictions. Population-coding frameworks similarly suggest that overlap at the level of individual ECoG electrodes or single neurons does not preclude separable population-level representations, as single low-dimensional neural manifolds within a single cortical area can support multiple distinct motor behaviors^46^. Here speech-related and gesture-related representations may remain distinguishable both when attempted alone and when attempted together, while the population pattern associated with each behavior shifts between isolated and concurrent contexts. A model trained only on isolated attempts could learn patterns that distinguish speech or gesture classes in that context, but those patterns may not fully generalize when the behaviors are attempted concurrently. This interpretation reconciles the successful decoding of both behaviors during concurrent attempts with the incomplete generalization of isolated-trained decoders, and the improved performance obtained by including simultaneous-context training.

Notably, prior intracortical spiking studies investigating simultaneous cursor control and speech^15,32^ did not explicitly train models for multi-effector contexts, which may have contributed to reduced performance during concurrent behavior. Provided one or more microelectrode arrays can capture separable, task-relevant signals for speech and body movements, similar context-inclusive training strategies may also improve simultaneous speech and body movement decoding from intracortical spiking recordings.

The nature of this overlap has been a topic of recent study. Some work suggests that the canonical somatotopy across the precentral gyrus is interleaved with regions that respond to multiple effectors and may support the planning and coordination of complex movements^33,47,48^. Intracortical work has similarly found whole-body representations throughout the region rather than purely following a strict somatotopy, including speech-preferential territory adjacent to and overlapping with traditionally limb-related cortex^27^. More broadly, grasp-related representations have been shown to extend beyond the canonical hand knob into the middle and ventral precentral gyrus^49–51^, while other recent work has shown that some of these putative multi-effector areas are critical for speech-motor sequencing and production^52,53^. Whether the overlapping activity observed here reflects the coordination of complex, multi-effector movements, the recruitment of nonoverlapping but spatially proximal neural populations or other mechanisms remains an important question for future studies that aims to understand the neural basis of movement planning and production.

A limitation of this proof-of-concept study is the restricted speech and gesture vocabularies used for simultaneous decoding, although this allowed us to sample all possible speech–gesture combinations. Scaling to larger vocabularies is necessary for practical use, but would make collecting data for every possible pairing impractical, increasing the importance of model generalization to unseen speech-and-gesture combinations. Encouragingly, offline simulations showed that speech and gesture models generalized to simultaneous speech-and-gesture combinations not observed during training, which suggests that only a limited number of simultaneous-training examples may be needed for models to generalize to unseen speech-and-gesture pairs. However, decoding performance was not necessarily uniform across effectors; in particular, ipsilateral movements were generally decoded less accurately than contralateral or speech-related behaviors, consistent with prior work showing weaker ipsilateral movement-related signals in the SMC^21^. In addition, although incorporating simultaneous-context training improved performance, generalization to concurrent speech-and-gesture attempts remained imperfect. This may reflect several factors, including increased cognitive demands during the simultaneous task that could result in greater trial-to-trial variability, and limited simultaneous-training data relative to the full variability of concurrent behavior. Overall, to assess the scalability of these approaches, further testing with larger speech and gesture repertoires, including balanced sampling of orofacial and bodily articulators, reducing latency by using incremental or continuous decoding approaches, and testing with other recording methodologies, such as noninvasive devices, is of future research interest. Finally, it is possible that participant differences, such as paralysis etiology or residual movement capability, could have impacted cortical representations or decoding performance. Given the small number of participants involved in this study, although findings were similar across participants, we cannot determine whether these factors differentially affected performance. Larger cohorts spanning different etiologies and degrees of paralysis will be needed to determine these potential effects.

Together, our results demonstrate a proof-of-concept multi-effector BCI in which a single ECoG implant supports parallel decoding of speech and upper-body gestures in restricted vocabularies, with decoded outputs rendered through a personalized full-body avatar. By showing that isolated-trained models do not automatically generalize to simultaneous behavior, and targeted training strategies can improve model performance and reduce false activations, we highlight key challenges for robust multi-effector decoding and offer a practical path toward addressing them. More broadly, these findings represent a step toward unified neuroprosthetic systems that combine multiple forms of communication, including spoken language and body movements, to support more flexible, expressive communication for people with paralysis.

## Methods

### Clinical trial overview

Three individuals participated in this research as part of the BCI Restoration of Arm and Voice (BRAVO) clinical trial (ClinicalTrials.gov registration: NCT03698149). The trial began in November 2018, and data collection for the present study occurred between September 2022 and November 2025. All three participants received $50 per data collection session (up to $5,000 annually) and provided CFR Part 11-compliant informed consent through DocuSign 21 for participation in the study and the release of recorded videos. This clinical trial is a phase I, single-center early-feasibility study designed to evaluate the long-term potential of ECoG-based neural interfaces to control advanced neuroprostheses for motor and communication restoration. The predefined primary outcomes are the safety of the investigational device, assessed by treatment-emergent adverse events and the feasibility of using ECoG signals to control devices for motor and communication restoration. The research presented here is not intended to inform conclusions about the primary outcomes of the clinical trial. The U.S. Food and Drug Administration approved the investigational use of the neural-implant device, and the study protocol was approved by the University of California, San Francisco, Institutional Review Board and the National Institute on Deafness and Other Communication Disorders, National Institutes of Health. The full clinical trial protocol is available in the Supplementary Information.

### Participants

There were slight differences in how each participant attempted the speech, gesture and other movements in this work, which are described at the end of this section.

Bravo-1r enrolled at the age of 36 years, has intact cognitive function and was diagnosed by neurologists and a speech-language pathologist with severe spastic quadriparesis and anarthria due to a bilateral pontine stroke at the age of 20 years^12,54,55^. Upon initial enrollment, Bravo-1r was implanted with a 128-channel ECoG array (4-mm center-to-center spacing)^12^. At the age of 41 years, the array was replaced with a higher-density of 253-channel ECoG array (3-mm center-to-center spacing; ‘Neural implant’). The designation ‘Bravo-1r’ refers to the higher-density implant. Although Bravo-1r can vocalize, he is unable to produce intelligible speech. To communicate, he relies on an augmentative and alternative communication interface that uses residual head movements to spell out words on a computer. Bravo-1r’s native language is Spanish and he has professional working proficiency in English.

Bravo-3 enrolled at the age of 47 years and has intact cognitive function. She was diagnosed with quadriplegia and anarthria by neurologists and a speech-language pathologist after a pontine stroke at the age of 30 years^8,11,55^. She is unable to produce intelligible speech and has difficulty in vocalizing, so she communicates through a Tobii Dynavox device that tracks head movements to type on a computer. Bravo-3’s native language is English.

Bravo-6 enrolled at the age of 60 years and was diagnosed with ALS at the age of 56 years. His cognitive function was intact, scoring 29/30 on the Mini-Mental State Examination and missing one point due to his physical inability to perform a task. His symptoms began at approximately the age of 50 years with progressive lower-extremity spasticity and impaired balance. At the time of data collection, the primary effect on his upper body was loss of fine finger movements and progressive vocal-tract paralysis, while he had retained a normal range of movement in his arms. Bravo-6 began to show dysarthria at the age of 57 years, progressing to the complete loss of intelligible speech within 9 months, and he has been diagnosed as anarthric by a speech-language pathologist. To communicate, Bravo-6 uses residual motor function in his arms and hands to type out messages on his phone and computer. While functional, typed communication is slow and effortful due to the loss of hand dexterity. Bravo-6’s native language is German and he has full professional proficiency in English.

Neural activity during multiple body movements was recorded from all three participants, as shown in Fig. 1. Although participants attempted roughly the same movements, exact attempt strategies differed. Attempt strategy (for example, overt, silent for speech or fully imagined) was determined by the participant’s residual capability and whether they found the strategy sustainable, such that their attempts would be consistent over time. Bravo-3 was unable to vocalize, so she silently attempted speech. While Bravo-1r could vocalize, he felt it was too fatiguing and preferred silent attempts. All participants performed instructed eye blinks normally. Bravo-1r was instructed to silently attempt speech and minimally attempt (that is, without overarticulating or exaggerating) all other body movements. This resulted in observable, but minimal and uncoordinated, movements during speech, orofacial movements, gestures and head movements, but no consistently observable movements for individual finger, shoulder, arm, trunk, leg or foot movements. Bravo-3 was instructed to silently attempt speech and attempt all other body movements. This resulted in minimal and uncoordinated movements during speech, orofacial movements and head movements, but no consistently observable movements for all other body movements. Bravo-6 was instructed to overtly attempt speech (that is, with vocalization), attempt orofacial movements and covertly attempt all other body movements. This resulted in audible but unintelligible vocalizations during speech, minimal and uncoordinated movements during orofacial and head movements, and no visible movements for all other body movements. Although instructed to imagine all nonorofacial body movements, Bravo-6 described the observed head movements as involuntary. Full-body avatar experiments were conducted after Bravo-3 withdrew from the trial. Accordingly, all avatar demonstrations and simultaneous speech-and-gesture results involve only participants Bravo-1r and Bravo-6.

### Neural implant

The neural-implant device used in this study consists of a high-density ECoG array (PMT) and a percutaneous pedestal connector (Blackrock Microsystems). The ECoG array is designed with 253 disk-shaped electrodes, organized in a grid pattern with 3-mm center-to-center spacing. Each electrode features a recording contact diameter of 1 mm and an overall diameter of 2 mm. The arrays were surgically implanted subdurally on the pial surface of the left hemisphere of the participants’ brains, targeting areas of speech production and motor movements. The percutaneous pedestal connectors, which were secured to the skull during the same operation, carry electrical signals from the ECoG array to an external digital headstage (CerePlex E256, Blackrock Microsystems). All three devices were implanted at the UCSF Medical Center with no surgical complications. Bravo-1r was implanted in April 2024, Bravo-3 in September 2022 and Bravo-6 in March 2025. For each participant, electrodes were localized by coregistering their pre-operative T1 scan to their postoperative computed tomography scan and reconstructing pial surfaces using FreeSurfer (v7.2.0) and the img_pipe package^56^. For Bravo-6, some brain shift^57^ was observed postoperatively, likely due to cortical atrophy associated with ALS^58^, that made exact electrode localization difficult. Despite this, functional responses (for example, listening responses that are unaffected by ALS) are approximately in the locations we expect, so broad spatial trends appear accurate.

### Task design

#### Copy task

The S-only, G-only and simultaneous speech-and-gesture tasks were designed following a copy-task paradigm. Each trial within these tasks follows a multiphase structure. A target stimulus was first presented on the screen. Next, four dots disappeared sequentially to cue the participant to prepare to attempt the target word and/or gesture. A green line then replaced the stimulus as a ‘go’-cue to attempt the target. Each trial ended with a 2 s blank intertrial interval. In the S-only task, targets were presented as text. In the G-only task, targets were presented as an animation of the movement. In the speech-and-gesture task, targets were presented as both text and an animation of the simultaneous gesture. Bravo-1r’s speech was silently attempted, and gestures were minimally attempted. Bravo-6’s speech was overtly attempted, and his gestures were imagined (see ‘Participants’ for attempt strategy details).

For Bravo-1r, the S-only dataset consisted of five phrases, the G-only dataset consisted of 4 gestures and the simultaneous speech-and-gesture dataset consisted of 12 conversationally realistic speech–gesture pairs selected from the S-only and G-only sets (Supplementary Table 3). For Bravo-6, we collected a larger dataset of 10 phrases, 10 gestures and 100 speech-and-gesture pairs across the S-only, G-only and simultaneous tasks (Supplementary Table 4). For Bravo-6, we included all possible speech–gesture combinations, including pairings that may be uncommon in natural conversation, so that every phrase occurred with every gesture and vice versa (Supplementary Table 5). This design prevented fixed correlations between speech and gesture labels that could allow either decoder to rely on the paired modality. For instance, the gesture decoder could otherwise learn to predict ‘wave’ from its repeated pairing with ‘hello’, rather than independently decoding the intended gesture.

#### Conversation paradigm

We designed a cued conversation paradigm to demonstrate real-time use of simultaneous speech-and-gesture decoding in an interactive setting. The experimenter conversed with participants verbally or through typing into a chatbox. For typed conversations, the participant could see the conversation text on the screen. The verbal conversation was conducted with both Bravo-1r and Bravo-6. However, the typed conversation was recorded with Bravo-6 only. Neural activity was recorded as the participant attempted to respond to a question or comment from the experimenter. The participant was cued to respond when three dots at the bottom of the screen disappeared after the experimenter’s prompt. The participant could respond using any phrase and/or gesture from the discrete set the speech and gesture models were trained on. Decoded speech was displayed on the monitor as text, and decoded gestures drove the animation of the avatar. In the typed conversation paradigm, the decoded gestures were displayed as text on the screen alongside decoded speech and used to drive corresponding avatar movements. If neither speech nor gesture was attempted, no text was displayed and no gesture was animated.

#### Sensory data

Data were collected to assess neural responses to hand and face sensations in each participant. In brief, sensations for individual fingers and facial locations were delivered using a hard-edged plastic or wooden applicator. For each sensory target, the applicator was applied with moderate pressure and gently moved across the surface. For example, for the right thumb, the applicator would be placed on the fingertip and moved along the pad of the fingertip. Participants confirmed that they could feel the sensation and that it was not painful. The same sensory targets were tested in each participant—the right and left thumb, index, middle, ring and pinky fingertips for the hands and the lower lip, upper lip and tongue tip for the face. Sensations were manually annotated from video recordings and aligned to neural data.

Across sensory targets, 13–20 sensations were performed and accurately annotated for Bravo-1r, 25–46 for Bravo-3 and 19–22 for Bravo-6. Sensations had an average duration of 0.77, 0.87 and 0.65 s for Bravo-1r, Bravo-3 and Bravo-6, respectively. The average time between successive sensations was 1.77, 2.03 and 1.39 s for Bravo-1r, Bravo-3 and Bravo-6, respectively.

### Data acquisition and preprocessing

Each contact on the implanted ECoG arrays recorded the local field potentials from the surface of the cortex at a sampling rate of 30 kHz. Signals were common average referenced and preprocessed to extract high-gamma activity (HGA; 70–150 Hz) and low-frequency signals (LFSs; 1–100 Hz) at a 200 Hz sampling rate^59^. We normalized the HGA and LFS features independently using a 30 s sliding-window *z* score in real time during data collection. All data collection and online decoding experiments were performed at the participant’s residence using custom Python (v3.8) software package (rtNSR) for recording neural activity, task management and deploying real-time decoding models^59^. Models were trained offline using NVIDIA Tesla V100 GPUs.

### Multi-effector sensorimotor maps

For the multi-effector sensorimotor maps in Fig. 1b, we used pretrial *z*-scored HGA from three datasets—motor, sensory and listening.

For motor movements, we defined three broad effector groups—head/eyes, hand/arm and speech/orofacial. All movements were collected using the copy-task paradigm, in which a target movement was presented followed by a go-cue to attempt that movement. We then took a window of HGA, from 0.25 s before to 1 s after the go-cue, for every trial and electrode. We averaged across time points within each window, and across trials within the three effector groups. Within each group, relative HGA values were normalized to the maximum across electrodes and any negative values were clipped to 0. This yielded relative HGA values between 0 and 1 for each electrode for the three groups. We used these three values to define the relative preference of each electrode, plotted as red-green-blue (RGB) color values. The electrode size and α values are also scaled relative to the maximum relative HGA value for each electrode, across the three groups.

To find electrodes that were also modulated by individual finger, lip or tongue sensations, we similarly took a 0.25 s window of HGA aligned to the annotated sensation onset and averaged across time points. Although participants were instructed to remain as still and relaxed as possible during sensations, there were some involuntary movements (typically pursing the lips) that occurred in most sensations. To identify electrodes with real sensory responses rather than motor-related activity, we further computed two-sided Kruskal–Wallis tests between the finger sensations (five right-hand fingers) and the facial sensations (lower lip, upper lip and tongue tip) using the time-averaged HGA. False discovery rate (FDR) correction for multiple comparisons was applied across electrodes. We also averaged the time-averaged HGA across trials for the finger and facial sensation groups, yielding a single average HGA value per electrode for each sensation group. Electrodes were deemed to have a true sensory response if they were both significant given the Kruskal–Wallis test and if their average HGA response was at or above the 67th percentile of responses for that sensation group. If an electrode had a significant sensory response for only the finger sensations, only the facial sensations, both or neither, they were plotted with a triangle, star, square or circle marker in Fig. 1b.

Finally, because the ECoG arrays in each participant covered the superior temporal gyrus, we computed listening responses during a passive listening task. In this task, each participant listened to a set of ten sentences with silence in between. We took a 1 s window of HGA aligned to the onset of each sentence and averaged across time points and trials. These values were normalized by the maximum response across electrodes and any negative values were clipped to 0, yielding a single average HGA value per electrode during listening. A spatial density of these responses was plotted on each participant’s brain reconstruction in Fig. 1b, by computing a smoothed two-dimensional histogram weighted by the average HGA value for each electrode.

### Multi-effector classification

The multi-effector decoding models shown in Fig. 1 and Supplementary Fig. 1 were trained using approaches similar to those in our previous work^54,55^. The HGA across electrodes, aligned to the window of (−1, 2) seconds relative to movement attempt, was used to train the models. The HGA was decimated by a factor of 6 before model training and evaluation. The model architecture was a one-dimensional temporal convolutional layer followed by a series of GRU layers and then a final fully connected layer with a softmax function where the output dimension was equal to the number of classes (43 for Bravo-1r and 45 for Bravo-3 and Bravo-6). Models were trained with stochastic gradient descent and the Adam optimizer. Evaluation was performed using tenfold cross-validation, as in prior work^54,55^.

### Relative high-gamma activity electrode maps

Relative cortical HGA maps in Fig. 2 and Supplementary Fig. 4 were computed separately for speech, gesture and simultaneous speech-and-gesture conditions. Within each condition, trials were grouped by class, and for each electrode the median HGA time course across trials was computed for each class. For each class, the peak HGA value within the 0–3 s period after the go-cue was extracted, and the maximum across classes was used as the summary for that electrode. Task-responsive electrodes were identified using electrode-wise Kruskal–Wallis tests comparing activity from each condition against pooled rest activity. Resulting *P* values were corrected across electrodes using Benjamini–Hochberg FDR (*α* = 0.05). For visualization, significant electrode values within each condition were scaled from 0 to 1 using min-max normalization, and electrode color was scaled according to the normalized HGA value. Nonsignificant electrodes were displayed only as small outlined circles on the cortical surface.

### Overlap score

For each electrode, the speech–gesture overlap score was calculated as the geometric mean of the normalized relative HGA values from the S-only and G-only conditions:

$${\rm{O}}_{e}=\sqrt{{\rm{S}}_{e}{\rm{G}}_{e}}$$

where S_*e*_ and G_*e*_ are the normalized speech-related and gesture-related HGA values for electrode *e*, respectively. Overlap scores were calculated only for electrodes that were significantly task responsive in both conditions. Higher scores indicate strong relative HGA modulation during both speech and gesture, whereas lower scores indicate weaker modulation during one or both conditions.

### Speech and gesture modeling

We trained participant-specific neural network models to decode attempted speech and gestures from ECoG activity. For the offline analyses in Fig. 3a–d, we trained separate speech and gesture models under three training-data conditions—isolated trials only, S + G trials only and hybrid training with both isolated and S + G trials. For the real-time closed-loop experiments in Fig. 5 and Supplementary Videos, the deployed speech and gesture models used the training configuration that performed best in offline analyses and were trained with both isolated and S + G trials. Specifically, the speech model was trained to classify discrete phrases using trials from the S-only copy task and the simultaneous speech-and-gesture copy task. The gesture model was trained to classify discrete gestures using trials from the G-only copy task and the simultaneous copy task. Each model produced one predicted label per trial.

We used the *z*-scored HGA and LFS signals streamed from the real-time system at 200 Hz as neural features^8^. Unless otherwise noted, the speech and gesture models were trained using both HGA and LFS features. The only exception was the Bravo-6 gesture model, where including LFS did not improve performance relative to using HGA alone; therefore, only HGA features were used for that model. All electrodes were included as input features.

Before model training and inference, features were downsampled from 200 Hz to 33.33 Hz for the speech models and 25 Hz for the gesture models. We then normalized HGA and LFS feature streams separately to have an L2 norm of 1 across all time steps for every channel. Models were trained on different time windows of neural activity aligned to the go-cue—for speech decoding, we used a window of (−1, 4) seconds during training for both participants; for gesture decoding, we used (0, 5) seconds for Bravo-1r and (0, 3) seconds for Bravo-6. To improve robustness to variability in the timing of attempted actions following the go-cue, we also implemented time-jitter augmentation during training (Supplementary Methods, Data augmentation). Models were trained offline in PyTorch (v2.0.1) and saved on the Linux computer at the participant’s residence for real-time inference.

#### Cross-modality training

Because the speech and gesture decoders ran in parallel during real-time use, both models included an explicit rest class to reduce false activations when the participant was not attempting the corresponding modality. The rest class was trained using the following two sources of negative examples: (1) ‘true-rest’ data collected in a dedicated rest task in which the participant was instructed not to speak and not to attempt any movement, and (2) trials from the opposite modality (for example, G-only trials contributed to the speech model’s rest class; S-only trials contributed to the gesture model’s rest class). For each model, rest-class training examples consisted of half true-rest trials and half opposite-modality trials. The output dimensionality of each model equaled the number of discrete phrases or gestures plus this rest class.

#### Model architecture

Speech and gesture models for both participants (Bravo-1r and Bravo-6) were trained using a convolutional recurrent neural network architecture^59^. Each decoder consisted of a one-dimensional temporal convolution layer (kernel size = 4, stride = 4) followed by two layers of bidirectional GRUs (hidden size = 274). The final output of the last GRU layer was passed through a fully connected linear layer to produce logits over all discrete classes plus rest (speech model—phrases + rest; gesture model—gestures + rest). A softmax function was used to convert the model logits to probability estimates. Dropout (*P* = 0.54) was applied after the convolution layer, across GRU layers and before the final linear layer to reduce overfitting.

Because the number of target classes differed across participants and modalities, the primary architectural difference across models was the dimensionality of the final output layer—the Bravo-6 speech and gesture models each classified 10 classes plus rest (11 total classes), the Bravo-1r speech model classified 5 classes plus rest (6 total classes) and the Bravo-1r gesture model classified 4 classes plus rest (5 total classes).

Models were trained using cross-entropy loss and the Adam optimizer (learning rate = 0.0005; batch size = 16). During training, gradients were clipped to an L2 norm of 1. Training used early stopping (patience = 5 epochs) based on validation accuracy, with ties in accuracy broken using validation loss.

#### Model ensembling

To improve robustness, ensembling was used for both the speech and gesture models. The final speech and gesture models consisted of an ensemble of ten models trained on cross-validation folds. Folds were generated by randomly holding out trials using stratified *k*-fold cross-validation to maintain balanced class distributions. We trained one model per fold using the same hyperparameters for each ensemble. The final predicted class was selected by averaging the class probabilities across the ten models in each ensemble and selecting the class with the highest mean probability.

### Offline evaluation

Offline performance on simultaneous speech-and-gesture trials was evaluated using stratified *k*-fold cross-validation over the simultaneous dataset. For each fold, the simultaneous dataset was split into a training portion and a held-out test portion. The gesture-model training pool for that fold was formed by concatenating all G-only trials with the training portion of simultaneous trials. Separately, the speech-model training pool for that same test fold was formed by concatenating all S-only trials with the training portion of simultaneous trials.

Within each test fold, the training pool was further split into ten stratified validation folds. For each fold, labels for simultaneous trials were mapped to the appropriate unimodal label space (for example, ‘hello + wave’ was mapped to ‘hello’ for the speech model and ‘wave’ for the gesture model). Label mapping and encoding were performed after splitting to preserve balanced class distributions within each training/validation fold. The best-performing model from each validation fold (by validation accuracy; ties broken by validation loss) was retained, and the resulting ten models were combined into an ensemble for evaluation on the held-out test fold. This procedure was repeated across ten test folds to compute average offline test accuracy for the speech and gesture models in each participant.

Separately, we performed an offline evaluation on whether model training could have been further improved by explicitly encouraging the models to represent each movement class similarly, whether it was performed in isolation or simultaneously. This was done by adding an auxiliary supervised contrastive loss (using cosine similarity), treating same-label embeddings from isolated S + G trials as positive pairs and encouraging them to become more similar relative to other examples. Although there were no significant improvements in accuracy for the gesture models in either participant, there were small improvements to the speech models (Supplementary Figs. 14 and 15), suggesting that further adjustments to model training could increase performance.

### Electrode contributions

To generate electrode contributions for the speech and gesture decoders, we used a gradient-based saliency method^60^. For each trained model, we computed the loss for a batch of input data and backpropagated to obtain the gradient of the loss with respect to the input data. To quantify electrode-level contributions, we took the absolute value of these gradients and averaged them across samples and time steps, yielding a single contribution score per channel. Electrodes with higher contribution scores have a greater impact on the model’s loss compared to those with lower scores, suggesting a greater influence on model outputs. This approach is analogous to methods used in convolutional neural networks to highlight important regions in an image^61^.

To compare how electrode contributions changed as a function of training context (Fig. 3e,f), we computed average electrode contribution maps separately for models trained on isolated behavior and for models trained on simultaneous speech-and-gesture data. For each decoder type, contribution scores were averaged across ten model instances within each training condition, yielding one mean contribution value per electrode. Models in each training condition were trained on the same quantity of data. Electrode significance was assessed separately for each training condition by comparing the observed mean contribution at each electrode against a bootstrap-derived null distribution generated from 110 null-model saliency maps for the corresponding condition (50,000 bootstrap samples). Empirical one-sided *P* values were computed for each electrode and corrected across electrodes using the Benjamini–Hochberg FDR procedure (*α* = 0.05). To facilitate comparison across training conditions, each mean contribution map was L1 normalized by dividing each electrode’s contribution value by the sum of contribution values across all electrodes, such that the normalized contributions across electrodes summed to 1. Electrode contribution change was then defined as the L1-normalized simultaneous-training contribution minus the L1-normalized isolated-training contribution. Positive values therefore indicate electrodes that contributed more to models trained on simultaneous data, whereas negative values indicate electrodes that contributed more to models trained on isolated data. Electrodes significant in either training condition were displayed on the cortical surface (Fig. 3e,f and Supplementary Fig. 6).

### FP and FN calculations

FPR is defined as the proportion of trials for which the speech or gesture model incorrectly predicted a phrase or movement when the true behavior was rest. FNR is defined as the proportion of trials for which the speech or gesture model incorrectly predicted rest when the true behavior was attempted speech or movement. Chance-level FPR and FNR were calculated as the probability of making the corresponding error under uniform random prediction across all output classes. Specifically, chance FPR was calculated as the number of nonrest output classes divided by the total number of output classes, whereas chance FNR was calculated as 1 divided by the total number of output classes.

To evaluate how the rest-class training strategy impacted FPR, we computed FPR from ensemble predictions on held-out test sets whose ground-truth label was rest, including (1) true-rest trials and (2) opposite-modality trials that were treated as rest for the corresponding model (for example, G-only trials for the speech model and S-only trials for the gesture model). To evaluate how training-data type impacted FNR, we computed FNR from ensemble predictions on held-out nonrest trials from both isolated (S-only or G-only) test sets and simultaneous speech-and-gesture test sets. For both FPR and FNR, we repeated evaluation across ten held-out test splits to obtain distributions of error rates.

### Real-time evaluation

Real-time decoding performance was quantified for (1) the G-only copy task (Bravo-6 = 5 blocks), (2) the speech-and-gesture copy task (Bravo-6 = 12 blocks) and (3) the conversational speech-and-gesture blocks (Bravo-6 = 5 blocks; Bravo-1r = 3 blocks). For each participant and task type, per-block accuracy was plotted as individual scatter points, and trial-level accuracy was summarized with a bootstrapped distribution visualized as a half-violin plot. Bootstrap distributions were computed by resampling trials across blocks with replacement (1,000 iterations) and recomputing accuracy for each resample.

#### Real-time G-only blocks

For Bravo-6, five G-only blocks were collected (100 total gesture trials, 10 rest trials), with two repetitions per gesture. For Bravo-1r, 12 G-only blocks were collected (200 total gesture trials, 20 rest trials), with blocks either including one or two repetitions per gesture.

#### Real-time speech-and-gesture blocks

In Bravo-6, 200 simultaneous speech-and-gesture trials were collected across 12 blocks. To quantify FPRs, additional isolated trials were interleaved within these blocks, including 20 S-only trials (used to quantify gesture-model FPR during speech) and 20 G-only trials (used to quantify speech-model FPR during gestures). A small number of rest trials were also included to assess false activations during rest; specifically, two rest trials contributed to the FPR calculations for both models.

#### Real-time conversational blocks

Conversational blocks were collected as a qualitative demonstration of combined use of speech and gestures in conversation. For Bravo-6, five blocks were collected with four trials per block (two simultaneous speech-and-gesture trials, one S-only trial and one G-only trial). For Bravo-1r, three blocks were collected with three trials per block (two simultaneous speech-and-gesture trials and one S-only trial).

#### Real-time FPR

FPR was defined as the proportion of trials for which a model predicted a nonrest label when the ground-truth label corresponded to rest (or to the opposite modality treated as rest for that model). For Bravo-6, speech-model FPR was computed from G-only trials (20 trials) and rest trials (2 trials), and gesture-model FPR was computed from S-only trials (20 trials) and rest trials (12 trials).

### Real-time avatar animation

All real-time experiments were run using two machines. A Linux machine (Ubuntu 18.04, Colfax International) was used for data acquisition, running tasks, online preprocessing and model inference. A separate Windows machine (Windows 11 Pro 64-bit, Titanus Technologies) ran Unreal Engine (v5.3.2) to render and display the full-body avatar. Speech and gesture predictions generated by models running on the Linux machine were transmitted to the Windows machine through the Transmission Control Protocol (TCP) connection for real-time animation in Unreal Engine. The average transmission latency from the Linux machine to the Windows machine through TCP was 7.5 ms. For complete hardware specifications of both machines, see Supplementary Methods, Hardware specifications.

Within Unreal Engine, avatar animation and on-screen text were controlled by a state machine that received TCP messages and parsed them depending on which socket sent the signal. At the start of a recording task, the participants observed their custom avatar in an idle pose, with the idle animation looping continuously until interrupted by an incoming message. TCP messages encoded either (1) task prompts to be attempted by the participant or (2) decoded outputs from the speech model, gesture model or both. Prompts were displayed at the top of the participant-facing monitor, decoded speech outputs were displayed as text at the bottom of the screen and decoded gesture outputs triggered the corresponding avatar animation. Each gesture animation was used once for each decoded output, after which the avatar returned to the idle pose.

All avatars were created in the Unreal Engine (v5.3.2) MetaHuman Creator software. Of the 11 gesture animations used in the tasks with all participants (including idle), 7 were made using Adobe Mixamo FBX assets and 4 were custom made using a Rokoko motion-capture suit. All animation files were mapped into the custom participant and experimenter avatars and further refined in Unreal Engine into 2–3 s animation sequences.

Before designing the full-body avatars, we interviewed two participants, Bravo-1r and Bravo-6, about their preferences for the avatar face, body, clothes and the virtual environment in which the avatars were placed. For each participant, three full-body avatar options were created based on this input, iteratively refined based on the participant feedback, and a final avatar was selected. The conversational partner avatar used in the conversation task with Bravo-6 was designed without participant feedback.

### Statistical analysis

All statistical analyses were performed using two-sided nonparametric tests. Unless otherwise noted, two-sided Mann–Whitney *U* tests (using the ‘SciPy’ Python package) were used for all comparisons. Where multiple comparisons were performed, *P* values were adjusted within each analysis using the Benjamini–Hochberg FDR correction (*α* = 0.05, using the ‘statsmodels’ Python package). Statistical details are provided in the corresponding figures (Figs. 2–4 and Supplementary Figs. 7–12, 14 and 15) and ‘Results’. Box plots show the median, 25th and 75th percentiles, whiskers extending to 1.5× interquartile range and outliers.

We calculated 95% CIs using nonparametric percentile bootstrap resampling with 10,000 bootstrap samples. For each analysis, the observations underlying the reported estimate were sampled with replacement, with each bootstrap sample containing the same number of observations as the original dataset. The reported summary statistic, either the mean or median, was recalculated for each bootstrap sample. The 2.5th and 97.5th percentiles of the resulting distribution of bootstrapped estimates were used as the 95% CI bounds.

### Reporting summary

Further information on research design is available in the Nature Portfolio Reporting Summary linked to this article.

## Online content

Any methods, additional references, Nature Portfolio reporting summaries, source data, extended data, supplementary information, acknowledgements, peer review information; details of author contributions and competing interests; and statements of data and code availability are available at https://doi.org/10.1038/s41593-026-02446-2.

## Supplementary information

Supplementary Information Supplementary Methods, Supplementary Figs. 1–15, Supplementary Tables 1–5 and Clinical Protocol.

Reporting Summary

Supplementary Data 1 Exact FDR-corrected *P* values and corresponding test statistics for Supplementary Figs. 7–12, 14 and 15.

Supplementary Video 1 Real-time decoding of gestures during the copy task with Bravo-6. This video shows an online demonstration of gesture decoding with Bravo-6. The participant is shown a target gesture as text at the top of the screen, which counts down to a go-cue. At the go-cue, the participant covertly attempts the target gesture. The neural activity during his attempt is passed to the gesture model that predicts the gesture and is subsequently used to animate the personalized avatar. The gesture model also predicts rest if no gesture was attempted.

Supplementary Video 2 Real-time decoding of simultaneous speech and gestures during the copy task with Bravo-6. This video shows an online demonstration of parallel, simultaneous speech-and-gesture decoding with Bravo-6. Similar to Supplementary Video 1, the participant is shown a target speech, target gesture or target speech-and-gesture combination to attempt at the go-cue. Bravo-6 overtly attempts speech targets and covertly attempts the gesture targets. The neural activity during his attempts are passed in parallel to a speech model and to a gesture model. Predicted speech is shown at the bottom of the screen as text while the predicted gesture animates the avatar. Both speech and gesture models can predict ‘rest’ if no speech and/or no gesture was attempted, allowing the system to predict during isolated speech attempts, isolated gesture attempts and simultaneous speech-and-gesture attempts. This framework enables flexibility when speech and gestures are attempted simultaneously or in isolation.

Supplementary Video 3 Real-time decoding of speech and gestures in conversation with Bravo-6. In this video, Bravo-6 uses the speech and gesture decoding framework as shown to respond to a conversational partner speaking to him. A go-cue countdown appears at the bottom of the screen, where Bravo-6 can attempt a response with a speech and/or gesture attempt. Predicted speech is shown at the bottom of the screen as text, while the predicted gesture animates the avatar. If no speech was predicted, no response is shown at the bottom of the screen and, if no gesture was predicted, the avatar remains idle.

Supplementary Video 4 Real-time decoding of speech and gestures in conversation with Bravo-1r. This video shows a similar demonstration as in Supplementary Video 3, but with Bravo-1r.

Supplementary Video 5 Real-time decoding of speech and gestures in conversation with a second virtual avatar with Bravo-6. This video shows a similar demonstration as in Supplementary Video 3, but with a second virtual avatar as the conversational partner. Similar to Supplementary Video 3, Bravo-6 uses the speech and gesture decoding framework to respond to a conversational partner. The conversational partner is a second virtual avatar (named Lexi) animated by various gestures. Dialog from the second avatar is shown in a chat log on the left side of the screen. The second avatar also gestures, and various gestures are also noted in the chat log. Go-cue countdowns for Bravo-6, at which time he can attempt a response, are shown at the bottom right of the screen. Bravo-6’s decoded speech is shown as text in the chat log (with the name Oskar). Decoded gestures animate his personalized avatar, and are also noted in the chat log. This video demonstrates a proof-of-concept framework for virtual communication.

## Data Availability

ECoG neural data relevant to this study are under restricted access according to our clinical trial protocol. The protocol allows to share de-identified data with researchers from other institutions upon reasonable request but prohibits us from making it publicly available. All ECoG neural data are available in a restricted-access Zenodo repository at https://doi.org/10.5281/zenodo.21829509 (ref. ^62^). Requests for access should be directed to E.F.C. and will typically be reviewed within 3 weeks. Approved recipients must maintain confidentiality of the data and may not redistribute without prior authorization from E.F.C. The clinical trial protocol is included in the Supplementary Information. Source data underlying all figures are publicly available in the GitHub repository associated with this paper.

## Code availability

Code to replicate the main findings of this study can be found on GitHub at https://github.com/ChangLabUcsf/Brosler2026_simultaneous_decoding.
