# Supplementary material for: Simultaneous speech and gesture decoding for multimodal communication in paralysis
Source: Nat Neurosci. 2026 Sep 14;29(10):2445–55. doi: 10.1038/s41593-026-02446-2 (PMC13633112; doi:10.1038/s41593-026-02446-2)
Supplement: Supplementary file 1 [file 41593_2026_2446_MOESM1_ESM.pdf]

nature neuroscience

Supplementary information

https://doi.org/10.1038/s41593-026-02446-2

Simultaneous speech and gesture decoding for multimodal communication in paralysis

In the format provided by the authors and unedited

Supplementary Information

Simultaneous speech and gesture decoding for multimodal communication in paralysis

Samantha C. Brosler[1,2,3*], Jessie R. Liu[1,2*], Alexander B. Silva[1,2,3*], Irina P. Hallinan[1], Cady M. Kurtz-Miott[1], Jonah F. B. Dunkel Wilker[1], Adelyn Tu-Chan[4], Karunesh Ganguly[2,4], and Edward F. Chang[1,2,3]

1 Department of Neurological Surgery, University of California, San Francisco, San Francisco, CA, USA.
2 Weill Institute for Neurosciences, University of California, San Francisco, San Francisco, CA, USA.
3 University of California, Berkeley-University of California, San Francisco Graduate Program in Bioengineering, Berkeley, CA, USA.
4 Department of Neurology, University of California, San Francisco, San Francisco, CA, USA.
*These authors contributed equally.
Corresponding author: Edward F. Chang (edward.chang@ucsf.edu)

Supplementary Methods 3
- Data augmentation 3
- Hardware specifications 3
- Real-time avatar system architecture 3
- Longitudinal stability analysis 4
- Embedding geometry analysis 4
- Supervised-contrastive loss analysis 5

Supplementary Figures 6
- Figure S1. Multi-effector classification confusion matrices for Bravo-6 and Bravo-1r. 6
- Figure S2. HGA speech and gesture maps for Bravo-6. 7
- Figure S3. HGA speech and gesture maps for Bravo-1r. 8
- Figure S4. Relative HGA maps and overlap score between speech and gestures for Bravo-1r. 9
- Figure S5. Learning curves for the speech and gesture models. 10
- Figure S6. Electrode contribution change versus overlap score for Bravo-1r. 11
- Figure S7. Context-inclusive training aligns isolated and simultaneous embeddings within context-specific embedding structure in Bravo-6. 12
- Figure S8. Context-inclusive training aligns isolated and simultaneous embeddings within context-specific embedding structure in Bravo-1r. 14
- Figure S9. Naturalness of speech-gesture pairings does not affect simultaneous decoding accuracy or leave-one-out generalization in Bravo-6. 16
- Figure S10. Longitudinal stability of the Bravo-6 gesture and speech decoders. 17
- Figure S11. Longitudinal stability of the Bravo-1r gesture and speech decoders. 19
- Figure S12. False positive rates and false negative rates for each model and participant. 21
- Figure S13. Offline simultaneous decoding results. 22
- Figure S14. Supervised contrastive loss improves speech model performance on simultaneous speech-and-gesture trials for Bravo-6. 23

Figure S15. Supervised contrastive loss improves speech model performance on simultaneous speech-and-gesture trials for Bravo-1r. 24
Supplementary Tables **25**
Table S1. Statistical comparisons for Figure 3a-d. 25
Table S2. Statistical comparisons for Figure 4b-c. 27
Table S3. Bravo-1r utterance sets. 29
Table S4. Bravo-6 utterance sets. 30
Table S5. Naturalness classifications for simultaneous speech–gesture combinations used with Bravo-6. 32
Supplementary Videos **38**
Video S1. Real-time decoding of gestures during the copy task with Bravo-6. 38
Video S2. Real-time decoding of simultaneous speech and gestures during the copy task with Bravo-6. 38
Video S3. Real-time decoding of speech and gestures in conversation with Bravo-6. 38
Video S4. Real-time decoding of speech and gestures in conversation with Bravo-1r. 38
Video S5. Real-time decoding of speech and gestures in conversation with a second virtual avatar with Bravo-6. 38
CLINICAL PROTOCOL **40**
Protocol Synopsis 41
Table of Contents 49
Investigator Agreement / Protocol Signature Page 50
1.0 Introduction 51
2.0 Clinical Study Outline 55
2.1 Study Objectives 55
3.0 Investigational Plan 55
4.0 Patient Eligibility 58
5.0 Study Device(s) 59
6.0 Study Procedure 60
7.0 Clinical Measurements 71
8.0 Data Management 73
9.0 Statistical Methods and Data Analysis 74
10.0 Regulatory Requirements 75
References 77

Supplementary Methods

Data augmentation

Prior to model training, we applied time-jitter augmentation to improve model robustness to small trial-to-trial variability in movement/speech onset timing. For each trial of the training data, we extracted a fixed analysis window surrounding the go-cue (speech: [-1, 4] s during training for both participants; gestures: [0, 5] s for Bravo-1r, and [0, 3] s for Bravo-6). We then generated augmented versions of each trial by shifting this window in time by jitter offsets of {−0.33, −0.25, 0, 0.25, 0.33} s. The jittered time windows were concatenated along the trial dimension, effectively increasing the training set size by the number of jitters while preserving the original labels for each augmented window. To avoid inflating performance estimates, no jitter augmentation was applied to validation or test data; these splits used only the unshifted time window locked to the go-cue.

Hardware specifications

The Linux machine was manufactured by Colfax International and the operating system was Ubuntu 18.04.6 LTS. Its hardware specifications were Intel Xeon(R) Gold 6146 CPU 3.20GHz x 48 core processor, one Quadro P400/PCIe/SSE2 and one NVIDIA Tesla V100 GPUs, and 502.6 GB RAM. The Windows machine ran Windows 11 Pro 64-bit, manufactured by Titanus Technologies, LLC. Its hardware specifications were two Intel Xeon Gold 6244 SP 3.6GHz (4.4GHz Turbo) 150W 25MB Cache (16 Cores / 32 Threads) processors, two liquid-cooled GeForce RTX 4090 24GB GDDR6X GPUs, and 128GB (8 x 16GB) 288-Pin DDR4 SDRAM 3200 (PC4 25600) ECC Server Memory.

Real-time avatar system architecture

The Linux and Windows machines were connected via an ethernet cable. To allow for cross-platform communication, networking sockets were used on both machines. The communication between the machines was one-way: the Linux machine sent data to the Windows machine; the Windows machine didn't send any data to the Linux machine. We used the Transmission Control Protocol (TCP) to stream data from the Linux machine to the Windows machine. The Linux machine ran Python software and relied on the Python socket library to transmit data to the Windows machine. The Windows machine ran Unreal Engine v5.3.2 software and relied on the plugin “[Blueprint] Socket Helper (TCP-UDP Client-Server)” to receive data from the Linux machine. The Linux machine program created the client sockets that sent data to the Windows machine, and the Windows machine program created the server sockets, which listened for and received data from the client sockets.

The average latency of getting model predictions from a 10-model ensemble while running both decoders in parallel was 0.215 seconds for the speech decoder and 0.210 seconds for the gesture decoder.

Longitudinal stability analysis

To evaluate the longitudinal stability of speech and gesture decoding without recalibration, we performed a post hoc analysis in which separate decoder ensembles were trained using cumulative training windows. Day 0 was defined as the date of implantation for each participant. For Bravo-6, four speech decoder ensembles and four gesture decoder ensembles were trained using data collected from post-implant day 97 through cutoff days 118, 185, 210, or 238. For Bravo-1r, five speech decoder ensembles and five gesture decoder ensembles were trained using data collected from post-implant day 39 through cutoff days 67, 95, 115, 132, or 213.

For each cutoff, speech decoders were trained using all available speech-only and simultaneous speech-and-gesture (S+G) training trials collected through that cutoff, whereas gesture decoders were trained using all available gesture-only and S+G training trials. Labels from S+G trials were mapped to phrase identity for speech decoding and gesture identity for gesture decoding. S+G blocks collected after each cutoff were excluded from training and used for longitudinal evaluation. After training, decoder parameters were frozen, and each decoder was evaluated on all subsequent S+G blocks without further training or recalibration. Each decoder was an ensemble of 10 models and used the neural features, preprocessing, architecture, and training hyperparameters described in the “Speech and gesture modeling” Methods subsection.

Accuracy was calculated separately for each held-out S+G block and plotted as a function of days since implantation. When multiple blocks were collected on the same day, day-level performance was calculated as the mean of the individual block accuracies, with error bars representing the s.e.m. across blocks. For each frozen decoder evaluated on at least three test days, longitudinal trends were assessed using the Pearson correlation between days since implantation and day-level mean accuracy. P-values were corrected for multiple comparisons using the Benjamini-Hochberg false-discovery-rate procedure separately within each participant and decoder type.

Embedding geometry analysis

We compared internal embeddings generated for held-out isolated and simultaneous speech-and-gesture (S+G) trials by speech and gesture decoders trained either on isolated trials alone or on both isolated and S+G trials. Embeddings were extracted from the final GRU layer before dropout and the final classification layer at each decoder’s best-validation epoch. Analyses used ten outer test folds, each containing ten inner validation-fold models.

Before calculating embedding metrics, isolated and S+G embeddings were standardized together. Metrics were then calculated separately for each target class. Normalized centroid distance was defined as the distance between the isolated and S+G class centroids relative to the average within-context spread of the embeddings. Centroid cosine similarity measured the similarity in the orientation of the two class centroids. Context-classification accuracy was calculated using a logistic-regression classifier trained within each target class to distinguish isolated from S+G embeddings using stratified cross-validation. To compare within-class geometry, PCA was performed separately on isolated and S+G embeddings, and principal angles were calculated between the resulting subspaces using up to five principal components.

For each inner-fold model, metrics were averaged across target classes and then averaged across the ten inner-fold models to obtain one value per outer fold. Decoders trained under different context conditions were paired by outer fold because they were evaluated on the same held-out trials. Statistical comparisons were performed across the ten paired outer-fold values using two-sided Wilcoxon signed-rank tests, with Benjamini-Hochberg FDR correction for multiple comparisons within each figure panel.

Supervised-contrastive loss analysis

We trained speech and gesture decoders on both isolated and S+G trials using standard cross-entropy loss with or without an additional supervised-contrastive loss applied to the final-GRU embeddings. Training batches contained matched target classes from both contexts. Embeddings with the same target label but from different contexts were treated as positive pairs, encouraging isolated and S+G embeddings of the same target to become more similar. The contrastive loss was added to the cross-entropy loss with auxiliary-loss weights of λ = 0.003, 0.01, 0.1, 0.2, or 0.3. The λ = 0 baseline used cross-entropy loss alone with the same batch-sampling procedure.

S+G test accuracy was evaluated after each training epoch. To summarize performance during early training, we calculated the normalized area under the S+G accuracy learning curve using trapezoidal integration over epochs 0-15 for Bravo-6 and epochs 0-12 for Bravo-1r. AULC was normalized by the length of the epoch interval so that differences remained in percentage-point units. Within each outer fold, AULC was averaged across the ten inner-fold models, and the λ = 0 value was subtracted from the value for each nonzero auxiliary-loss weight. Each weight was therefore compared with its matched baseline from the same outer test fold.

Statistical comparisons were performed across the ten paired outer-fold differences using two-sided Wilcoxon signed-rank tests. Benjamini-Hochberg FDR correction was applied across the auxiliary-loss weights separately within each decoder.

Supplementary Figures

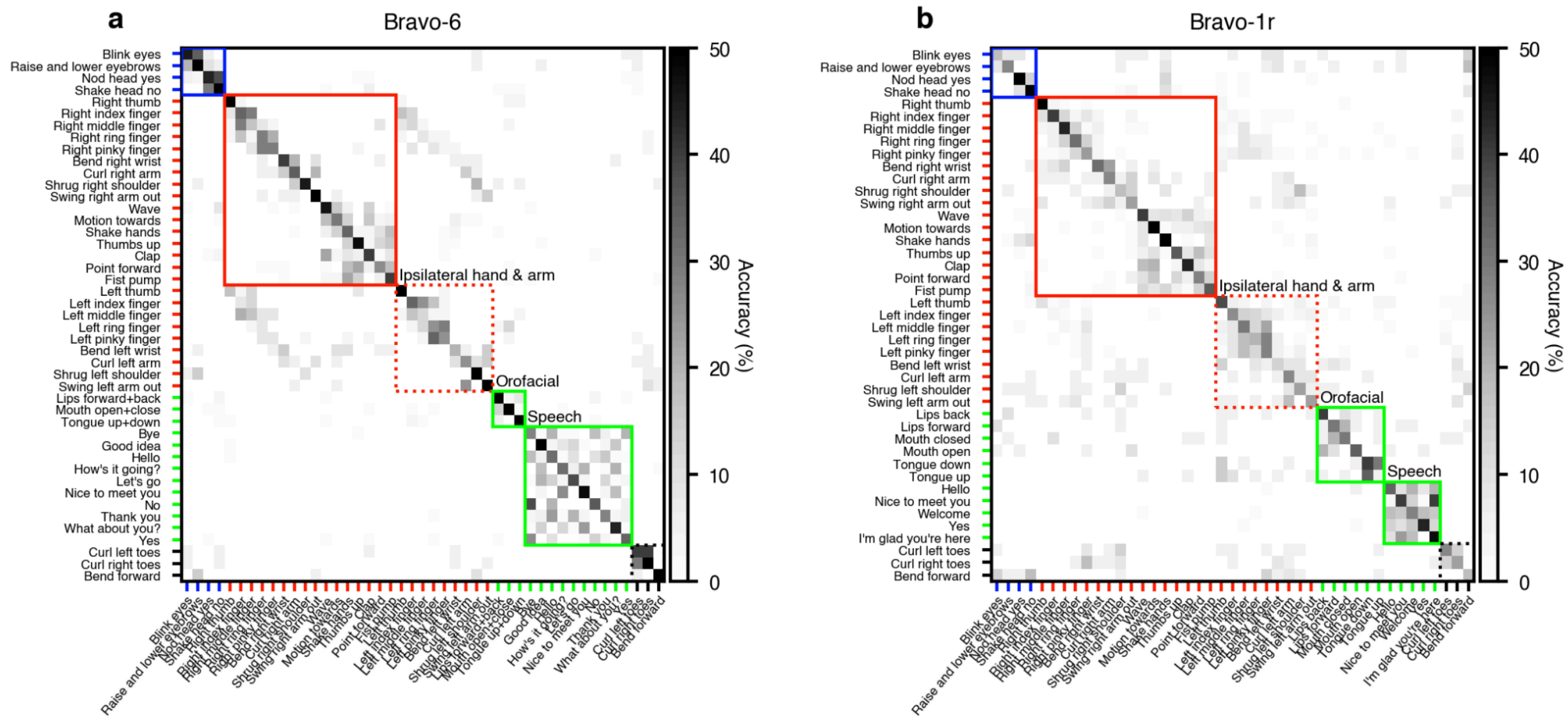

Figure S1. Multi-effector classification confusion matrices for Bravo-6 and Bravo-1r.

(a) Confusion matrix for the classification of several multi-effector movements for Bravo-6. Colored ticks and boxes are similarly defined as in Fig. 1c and correspond to the multi-effector groups shown in Fig. 1b. Blue is head or eye movements, red is hand or arm movements, green is speech and orofacial movements, and black corresponds to other movements, including torso, leg, and foot movements. (b) Confusion matrix as in (a), for Bravo-1r. Movements in the dotted boxes are not a part of the maps in Fig. 1b.

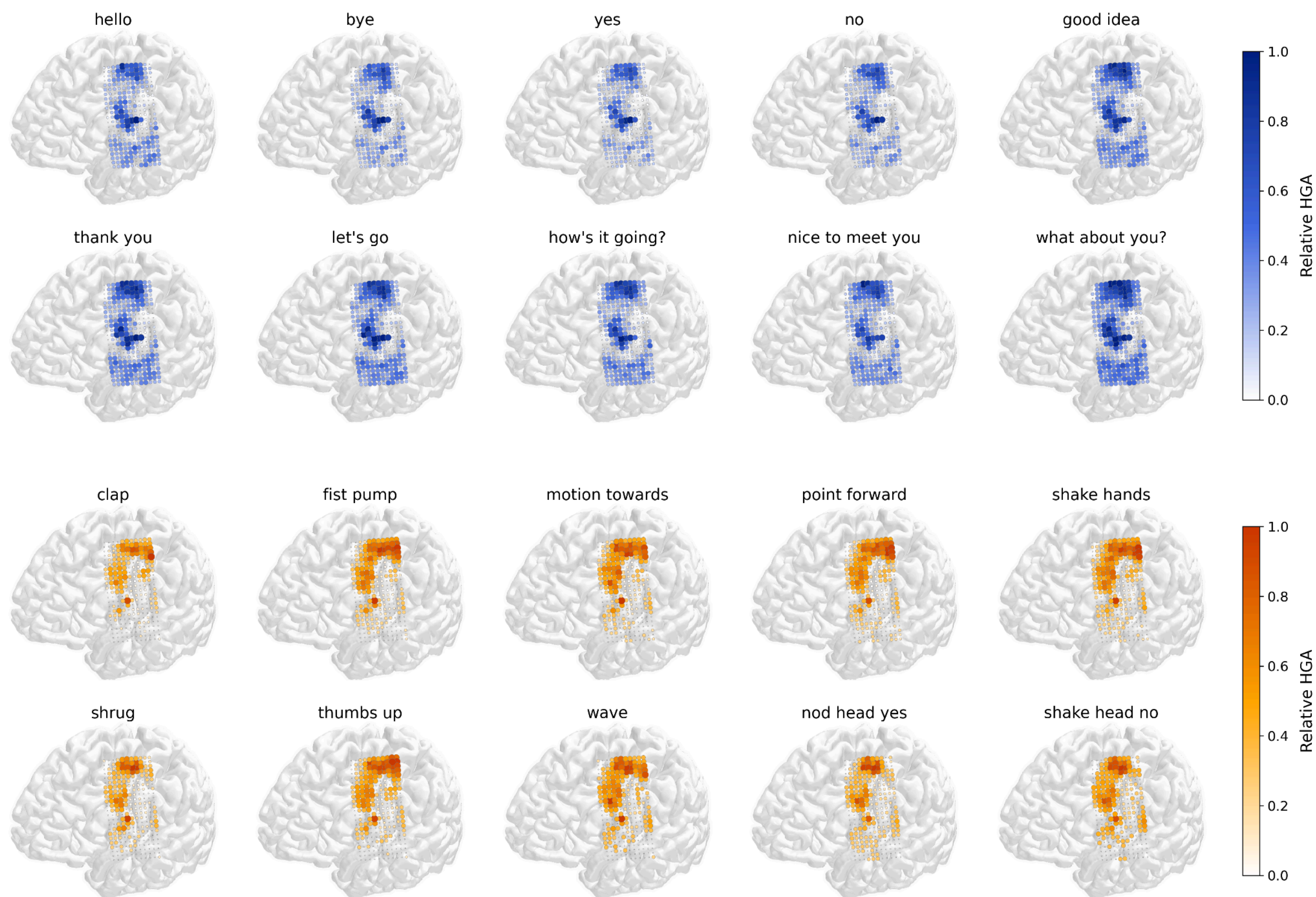

Figure S2. HGA speech and gesture maps for Bravo-6.

Relative high-gamma activity (HGA) for each of the 10 speech phrases in the speech-only condition (blue) and each of the 10 gestures in the gesture-only condition (orange) for participant Bravo-6. For electrodes with significant HGA, the relative HGA value is plotted with larger electrode sizes and darker colors indicating a higher relative HGA value. Non-significant electrodes are shown as smaller unfilled circles. See Methods for details on how the maps were computed.

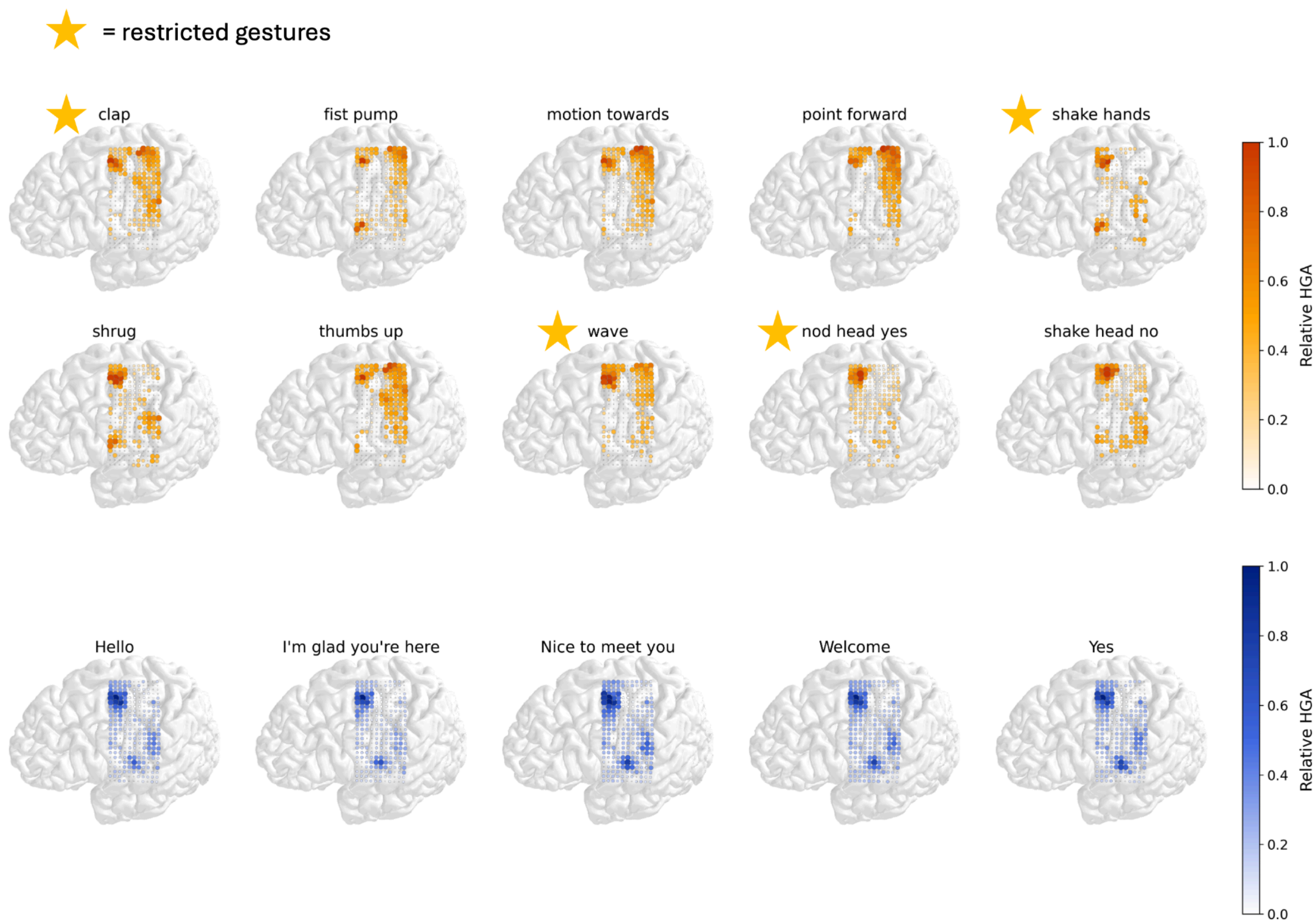

Figure S3. HGA speech and gesture maps for Bravo-1r.

Relative high-gamma activity (HGA) for each of the 10 gestures (orange) and 5 speech phrases (blue) in participant Bravo-1r. Stars indicate gestures included in the restricted gesture set, which appeared during S+G trials. For electrodes with significant HGA, the relative HGA value is plotted with larger electrode sizes and darker colors indicating a higher relative HGA value. Non-significant electrodes are shown as smaller unfilled circles. See Methods for details on how the maps were computed.

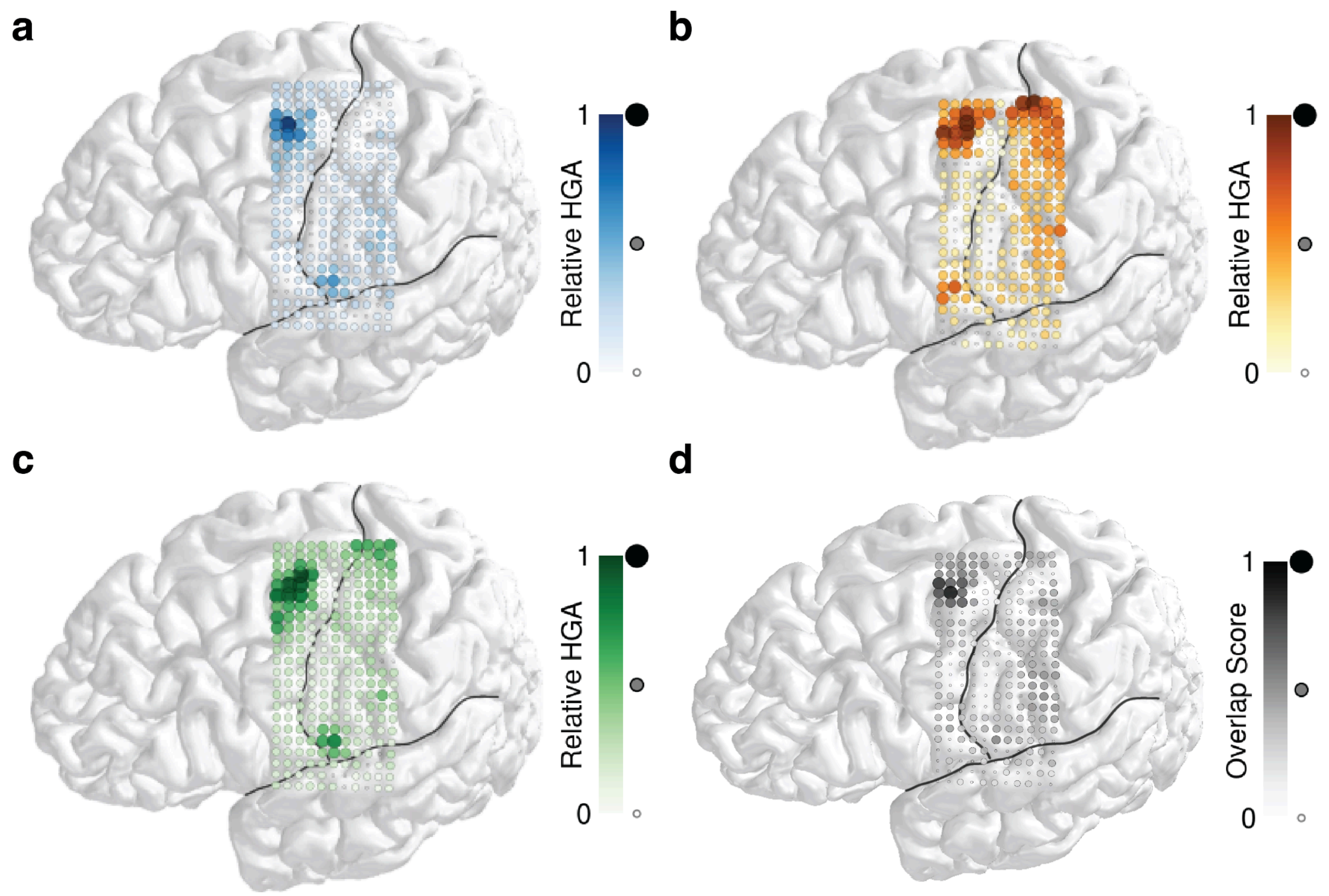

Figure S4. Relative HGA maps and overlap score between speech and gestures for Bravo-1r.

(a) Relative high-gamma activity (HGA) maps for speech in Bravo-1r, averaged across the different speech phrases. For electrodes with significant HGA, the relative HGA value is plotted with larger electrode sizes and darker colors indicating a higher relative HGA value. Non-significant electrodes are shown as smaller unfilled circles. (b) Same as (a), but across the restricted four-gesture set. (c) Same as (a), but for the simultaneous speech-and-gesture pairs. (d) Similar to (a) but plotting the overlap scores between speech and gestures. Higher overlap scores indicate electrodes with stronger relative HGA modulation during both behaviors, whereas lower overlap scores indicate weaker modulation in one or both conditions. Non-significant electrodes are shown as small dots. (a-c) are analogous to the analyses in Fig. 2d and (d) is analogous to the analysis in Fig. 2f.

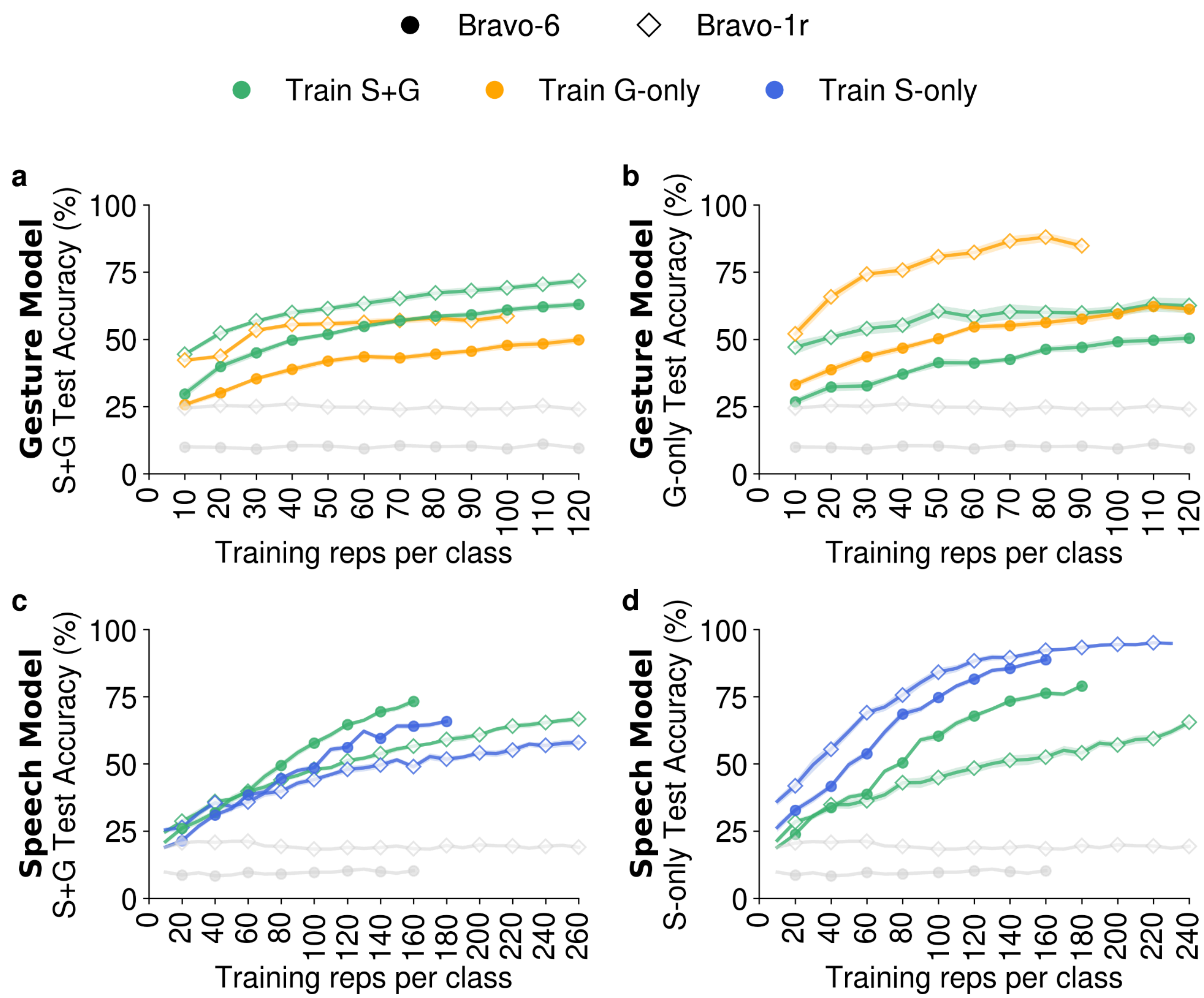

Figure S5. Learning curves for the speech and gesture models.

(a) Gesture model learning curves on held out S+G trials as a function of training data quantity, using exclusively isolated data (G-only or S-only) or S+G data. (b) Gesture model learning curves on held out G-only trials as a function of training data quantity, using exclusively isolated data (G-only or S-only) or S+G data. (c) Same as (a), but for the speech model. (d) Same as (b), but for the speech model. In all panels, color indicates training dataset, while marker style indicates the participant. At each training-data quantity, learning curves show the mean of n = 10 ensemble test accuracies, one from each held-out test fold; each ensemble combines predictions from 10 models trained using 10-fold cross-validation. Shaded regions indicate ±1 s.e.m. across the 10 ensemble test accuracies. In all panels, grey lines indicate chance accuracy, computed by shuffling the training data labels.

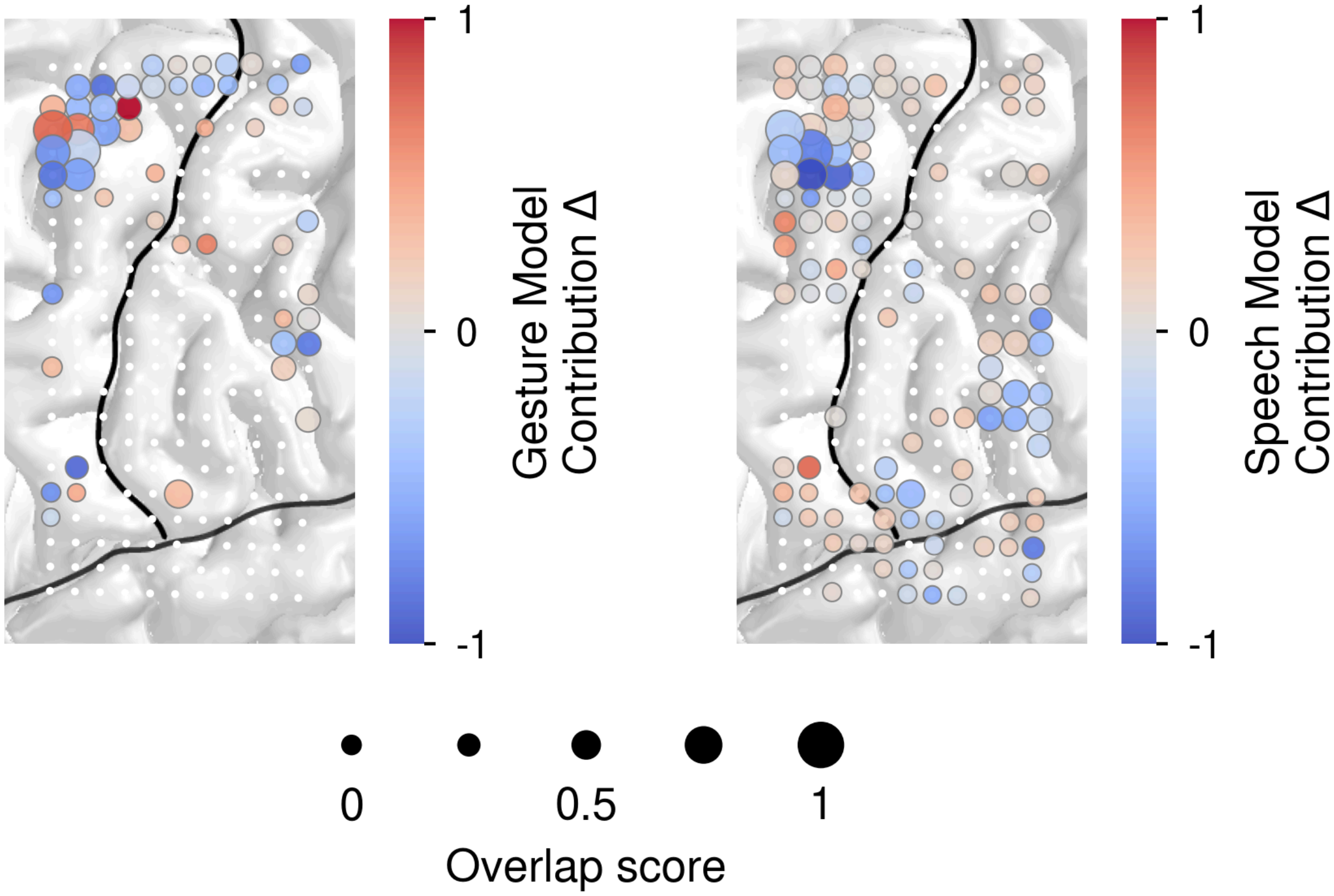

Figure S6. Electrode contribution change versus overlap score for Bravo-1r.

(left) Cortical map of the change in electrode contribution scores between Bravo-1r gesture models trained on the isolated dataset and those trained on the simultaneous dataset. (right) Cortical map of the change in electrode contribution scores between Bravo-1r speech models trained on the isolated dataset and those trained on the simultaneous dataset. Warmer colors indicate electrodes that contributed more when models were trained on S+G data, whereas cooler colors indicate electrodes that contributed less. The relative size of electrodes is scaled by overlap score (Fig. S4d). These panels are analogous to those in Fig. 3e and f.

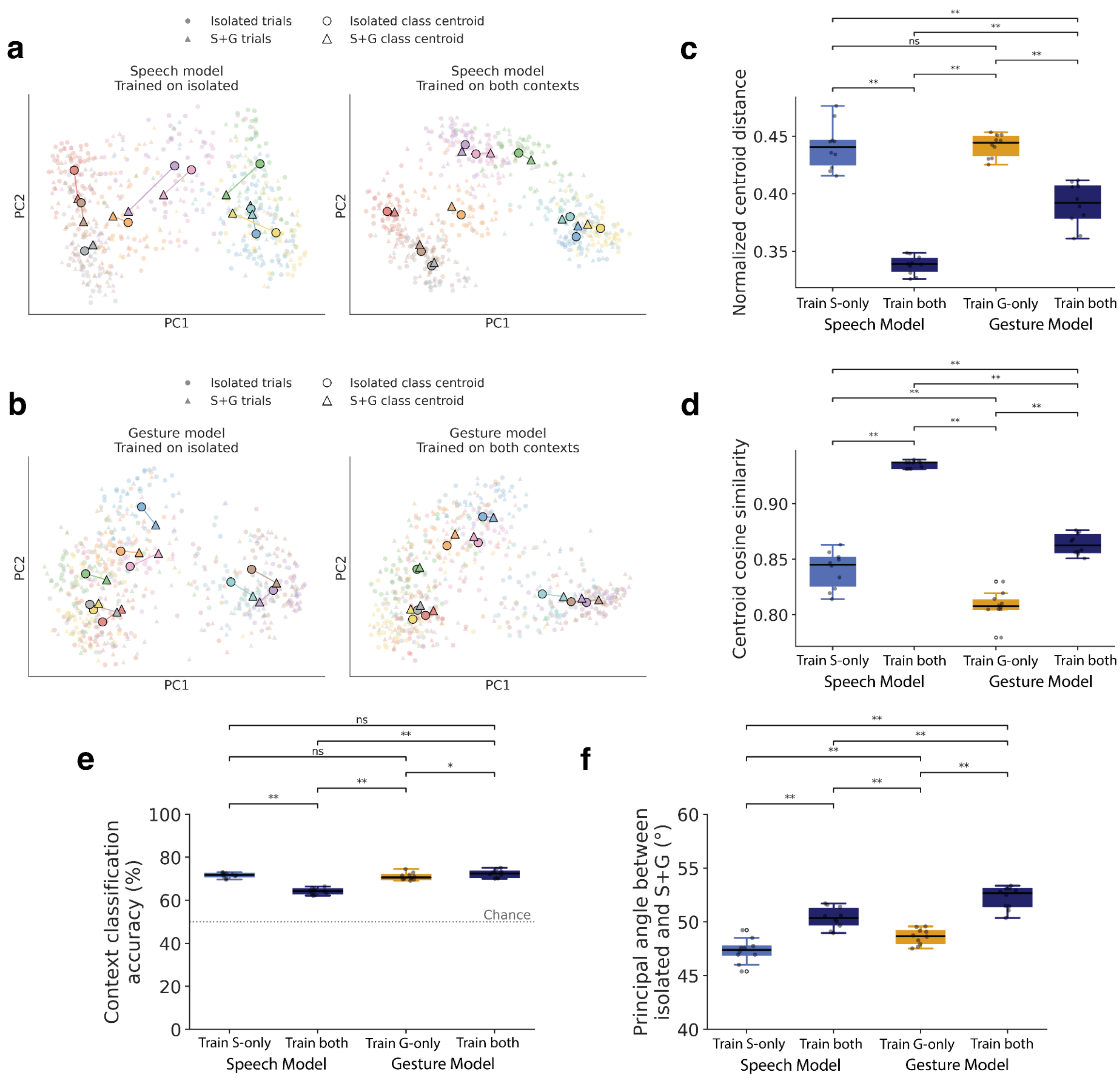

Figure S7. Context-inclusive training aligns isolated and simultaneous embeddings within context-specific embedding structure in Bravo-6.

(a) Principal-component projections of final-GRU embeddings from held-out isolated and simultaneous speech-and-gesture (S+G) trials for speech decoders trained on isolated trials alone (left) or on both isolated and S+G trials (right). (b) Principal-component projections for the corresponding gesture decoders. Points represent individual trials, colors indicate target classes, and outlined circles and triangles indicate isolated and S+G class centroids, respectively. (c) Normalized centroid distance between same-label isolated and S+G embeddings. Lower values indicate closer centroid alignment. (d) Centroid cosine similarity between same-label isolated and S+G embeddings. Higher values indicate closer centroid alignment. (e) Accuracy of linear classifiers trained separately within each target class to distinguish isolated from S+G embeddings. The dotted line indicates 50% chance accuracy. (f) Principal angle between the PCA subspaces of same-label isolated and S+G embeddings. Larger angles indicate less similarly oriented directions of trial-to-trial variation. In (c–f), each boxplot shows the distribution of n

= 10 outer-fold means per decoder-training condition; each point represents one outer-fold mean, obtained by averaging the metric across target classes and inner-fold models. Decoders trained under different context conditions were paired by outer fold because they were evaluated on the same held-out test trials. Boxplots show the median (center line), the 25th and 75th percentiles (box limits), and the whiskers extend to the minimum and maximum values within 1.5 times the interquartile range. Statistical comparisons were performed across the ten paired outer-fold measurements using two-sided Wilcoxon signed-rank tests with Benjamini–Hochberg FDR correction across the comparisons within each panel. * $P < 0.05$, ** $P < 0.01$; ns, not significant. Exact P-values for all comparisons are provided in the Supplementary Data 1 file.

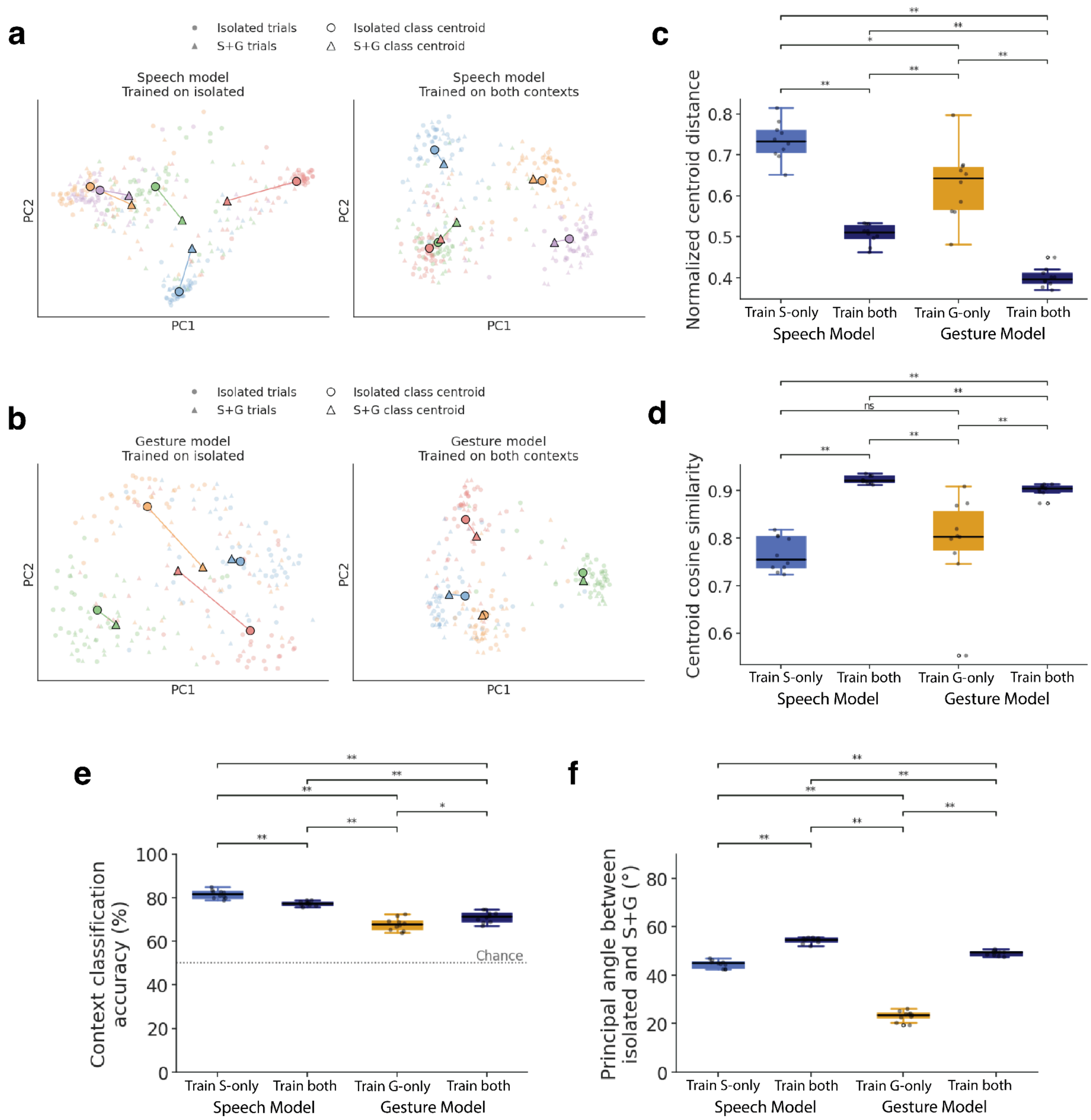

Figure S8. Context-inclusive training aligns isolated and simultaneous embeddings within context-specific embedding structure in Bravo-1r.

(a) Principal-component projections of final-GRU embeddings from held-out isolated and simultaneous speech-and-gesture (S+G) trials for speech decoders trained on isolated trials alone (left) or on both isolated and S+G trials (right). (b) Principal-component projections for the corresponding gesture decoders. Points represent individual trials, colors indicate target classes, and outlined circles and triangles indicate isolated and S+G class centroids, respectively. (c) Normalized centroid distance between same-label isolated and S+G embeddings. Lower values indicate closer centroid alignment. (d) Centroid cosine similarity between same-label isolated and S+G embeddings. Higher values indicate closer centroid alignment. (e) Accuracy of linear classifiers trained separately within each target class to distinguish isolated from S+G embeddings. The dotted line indicates 50% chance accuracy. (f) Principal angle between the PCA subspaces of same-label isolated and S+G embeddings. Larger angles indicate less similarly oriented directions of trial-to-trial variation. In (c–f), each boxplot shows the distribution of n

= 10 outer-fold means per decoder-training condition; each point represents one outer-fold mean, obtained by averaging the metric across target classes and inner-fold models. Decoders trained under different context conditions were paired by outer fold because they were evaluated on the same held-out test trials. Boxplots show the median (center line), the 25th and 75th percentiles (box limits), and the whiskers extend to the minimum and maximum values within 1.5 times the interquartile range. Statistical comparisons were performed across the ten paired outer-fold measurements using two-sided Wilcoxon signed-rank tests with Benjamini–Hochberg FDR correction across the comparisons within each panel. * $P < 0.05$, ** $P < 0.01$; ns, not significant. Exact P-values for all comparisons are provided in the Supplementary Data 1 file.

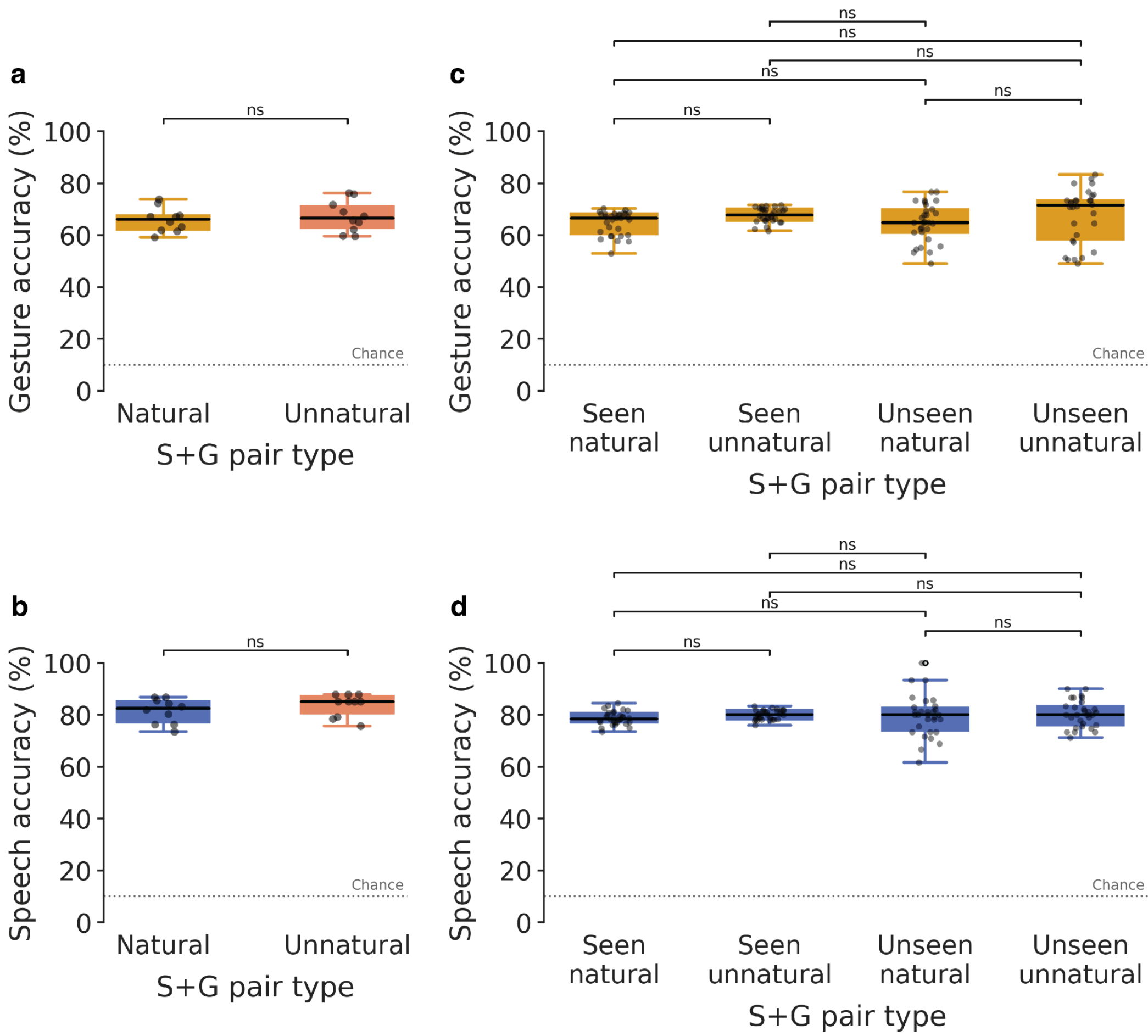

Figure S9. Naturalness of speech-gesture pairings does not affect simultaneous decoding accuracy or leave-one-out generalization in Bravo-6.

Bravo-6 attempted all 100 possible combinations of 10 speech phrases and 10 gestures, including pairs that were less common in natural communication. Each pair was annotated with a binary natural/unnatural label, and decoding accuracy was compared across pair types. (a) Offline held-out simultaneous speech+gesture (S+G) decoding accuracy for natural versus unnatural test pairs for the gesture decoder across n = 10 test splits. (b) Same as (a), but for the speech decoder, also with n = 10 test splits. (c) Leave-one-out generalization analysis separating natural and unnatural pairs for the gesture decoder. Gesture decoding accuracy was evaluated on S+G trials containing phrases that were either included during S+G training ("seen") or withheld from S+G training ("unseen"). Seen and unseen test trials were then further separated by pair naturalness. (d) Same as (c), but for the speech decoder, where seen and unseen refer to S+G trials containing gestures that were included or withheld from S+G training, respectively. Boxplots for (c) and (d) show distribution of test accuracy across n = 30 test splits. For all panels, each point represents one ensemble accuracy estimate from one held-out test split. Chance accuracy is shown for all panels as a dotted line. Pairwise comparisons were evaluated using two-sided Mann-Whitney U tests with FDR correction for multiple comparisons (6-way for each panel in (c) and (d)). Significance is reported as "ns" for non-significant, *$P < 0.05$, **$P < 0.01$, and ***$P < 0.001$. Boxplots show the median (center line), the 25th and 75th percentiles (box limits), and the whiskers

extend to the minimum and maximum values within 1.5 times the interquartile range. Exact P-values for all comparisons are provided in the Supplementary Data 1 file.

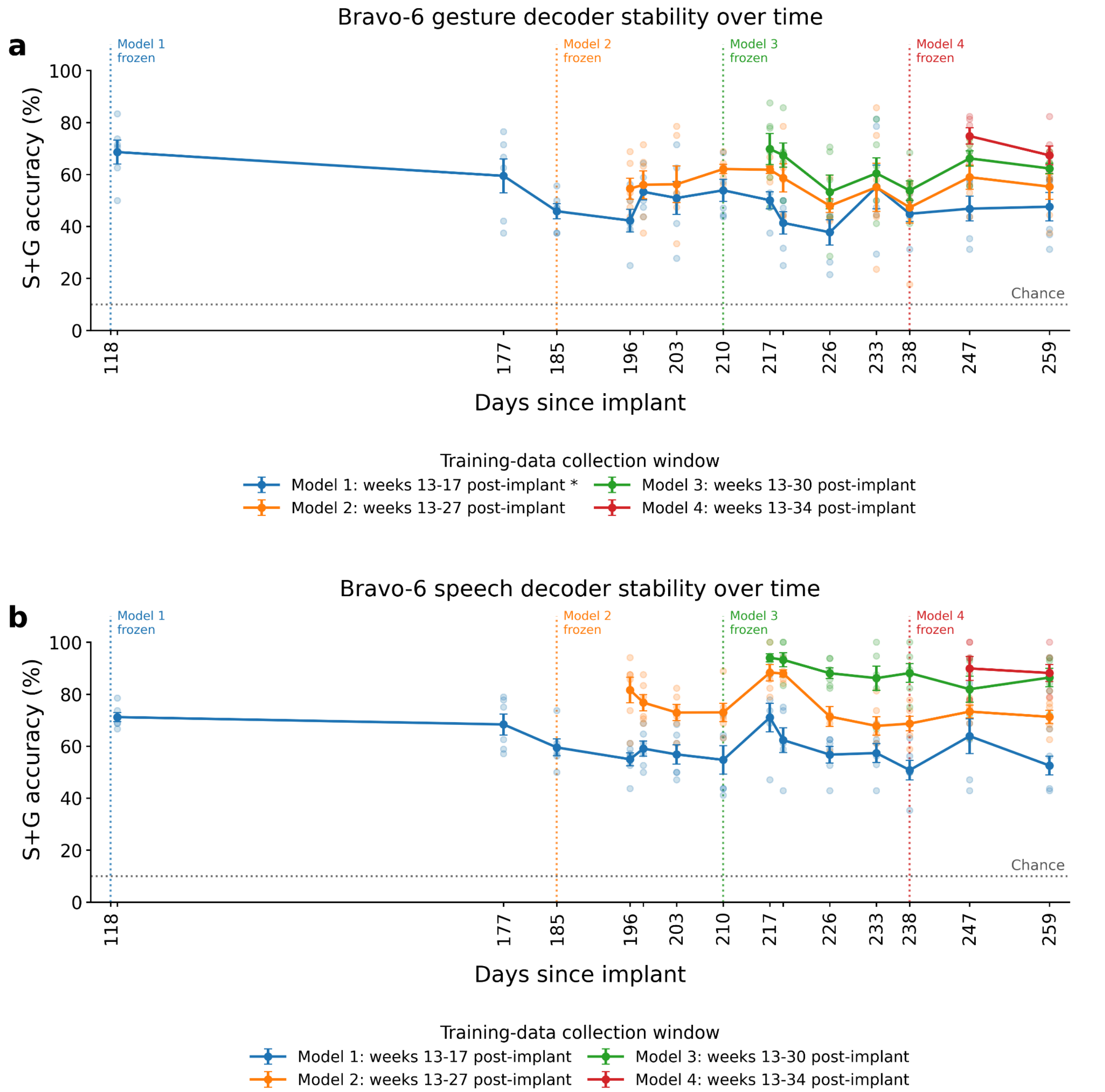

Figure S10. Longitudinal stability of the Bravo-6 gesture and speech decoders.

Four decoders for each modality were trained using cumulative isolated-behavior and S+G training data collected from about 3 months after implantation through four cutoff dates: about 4, 6, 7, and 8 months after implantation. After each cutoff date, the corresponding decoder was frozen and evaluated without further parameter updates on all S+G blocks collected after that date. The gap between 4 and 6 months reflects a period during which Bravo-6 was unavailable for data collection. (a) Gesture decoder accuracy on S+G blocks. (b) Speech decoder accuracy on S+G blocks. Dark circles with error bars show the mean ± s.e.m. across n = 6 S+G blocks collected on each date, and lines connect the date-level means. Light circles show accuracy for individual blocks. Colored vertical dotted lines indicate the final training date for each decoder, and the horizontal dotted line indicates chance accuracy (10%). Each decoder consisted of an ensemble of 10 models. For each frozen decoder, we tested whether mean accuracy changed linearly over time using two-sided Pearson correlations across all post-cutoff collection dates. P values were

FDR-corrected across all decoder-specific tests. An asterisk next to a model name indicates a significant linear temporal trend after FDR correction (FDR-adjusted $P < 0.05$). Temporal trends were not tested for decoders with fewer than four post-cutoff collection dates. Exact P-values for all comparisons are provided in the Supplementary Data 1 file.

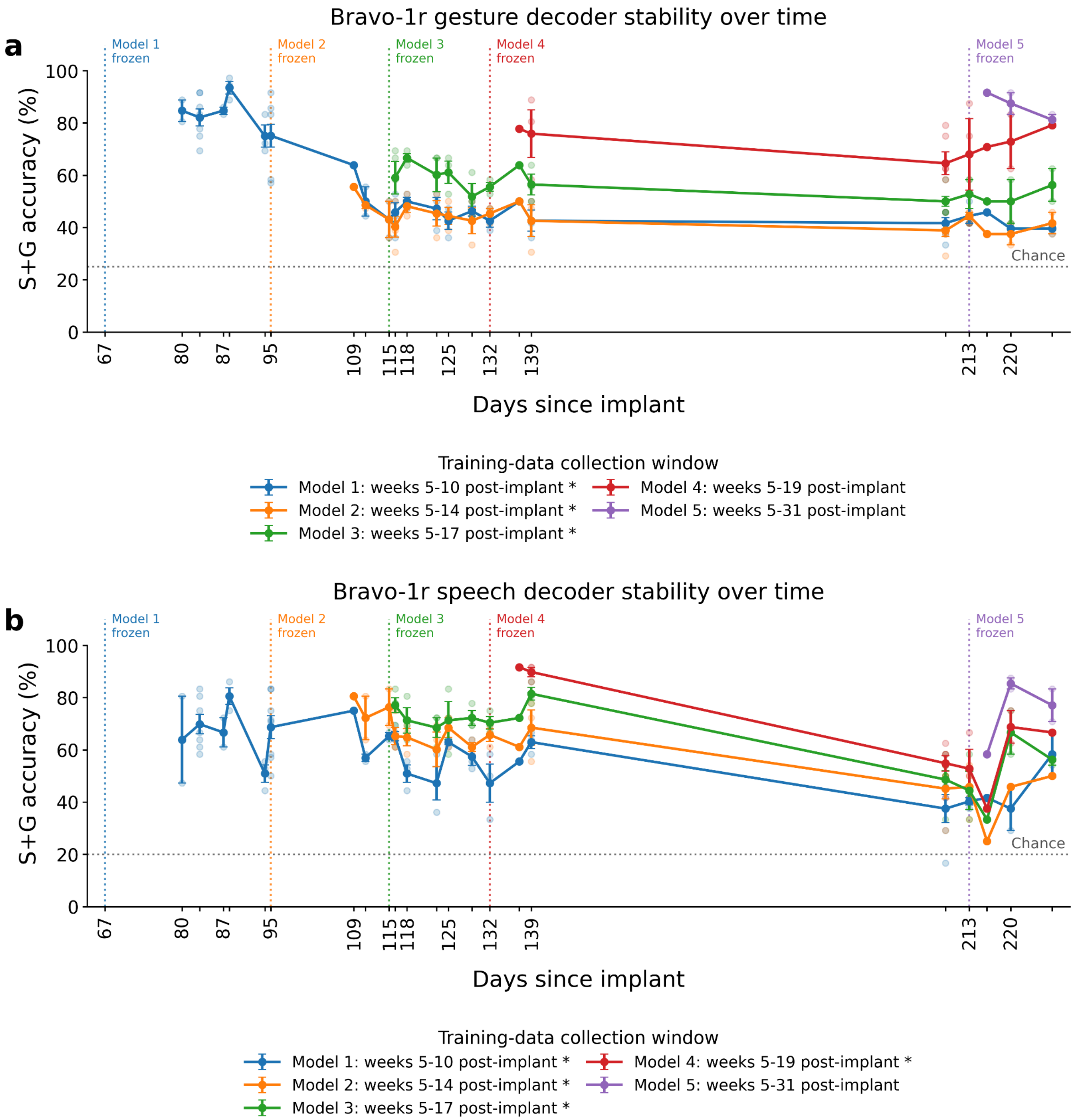

Figure S11. Longitudinal stability of the Bravo-1r gesture and speech decoders.

Five decoders for each modality were trained using cumulative isolated-behavior and S+G training data collected from about 1 month after implantation through five cutoff dates: about 2, 3, 4, 4.5, and 7 months after implantation. After each cutoff date, the corresponding decoder was frozen and evaluated without further parameter updates on all S+G blocks collected after that date. The gap between about 4.5 and 7 months reflects a period during which Bravo-1r was unavailable for data collection. a, Gesture decoder accuracy on S+G blocks. b, Speech decoder accuracy on S+G blocks. Dark circles with error bars show the mean ± s.e.m. of block accuracy for each collection date, and lines connect the date-level means. Light circles show accuracy for individual blocks. For both panels, n = 1 block on days 109, 137 and 216; n = 2 blocks on days 80, 87, 111, 115, 220, 227; n = 3 blocks on days 88, 94, 118, 123, 125, 129, 132,

139, 213; n = 4 blocks on day 116; n = 6 blocks on day 209; n = 7 blocks on day 83; and n = 8 blocks on day 95 after implantation. Error bars show s.e.m. across blocks when n > 1; no error bar is shown for collection dates with n = 1. Colored vertical dotted lines indicate the final training date for each decoder. Horizontal dotted lines indicate chance accuracy (25% for gesture and 20% for speech). Each decoder consisted of an ensemble of 10 models. For each frozen decoder, we tested whether mean accuracy changed linearly over time using two-sided Pearson correlations across all post-cutoff collection dates. P values were FDR-corrected across all decoder-specific tests. An asterisk next to a model name indicates a significant linear temporal trend after FDR correction (FDR-adjusted $P < 0.05$); all significant trends were negative. Temporal trends were not tested for decoders with fewer than four post-cutoff collection dates. Exact P-values for all comparisons are provided in the Supplementary Data 1 file.

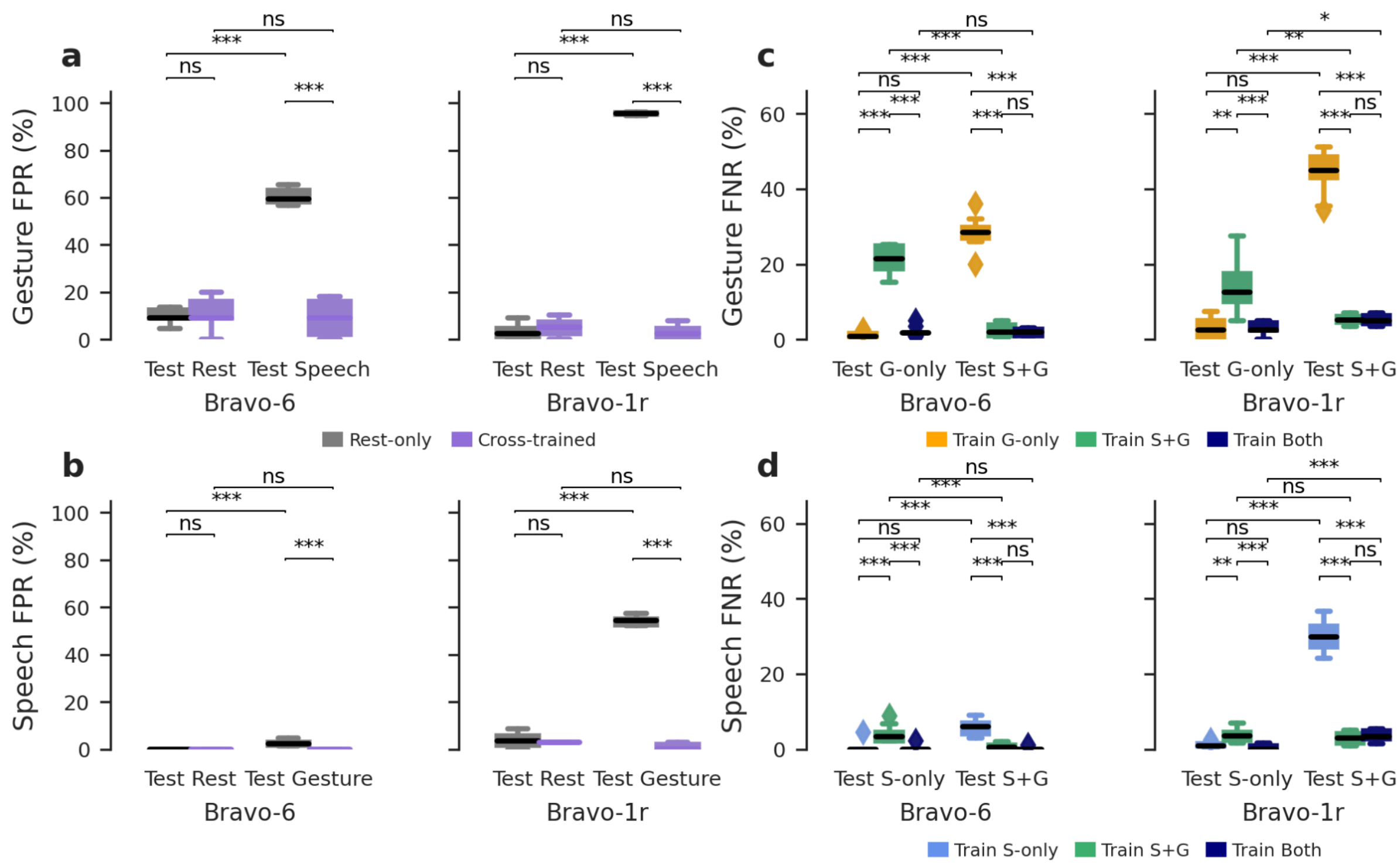

Figure S12. False positive rates and false negative rates for each model and participant.

(a) False positive rate (FPR) of the gesture model when the rest class was trained using only true rest trials ("Rest only") or with equal amounts of true rest trials and the opposite modality (e.g. addition of S-only trials, "Cross-trained"), for both Bravo-6 (left) and Bravo-1r (right). FPR was evaluated both on rest trials (see "Test Rest") and on the cross-modality trials (see "Test Speech"). (b) Same as (a), but for the speech model (here, opposite modality trials were gesture trials). (c) False negative rate (FNR) of the gesture model when trained on isolated data (G-only), simultaneous data (S+G), or both datasets ("Both"), for both Bravo-6 (left) and Bravo-1r (right). FNR was evaluated both on isolated and simultaneous datasets. (d) Same as (c), but for the speech model. In all panels, boxplots indicate the distribution of error rates across n = 10 test folds. For each participant, pairwise comparisons between training and test conditions were evaluated using two-sided Mann-Whitney U tests with FDR correction for multiple comparisons (4-way for each panel in (a) and (b), 9-way for each panel in (c) and (d)). Boxplots show the distribution of error rates across n = 10 test folds with the median (center line), the 25th and 75th percentiles (box limits), and the whiskers extending to the minimum and maximum values within 1.5 times the interquartile range. Significance is reported as "ns" for non-significant, *$P < 0.05$, **$P < 0.01$, and ***$P < 0.001$. Exact P-values for all comparisons are provided in the Supplementary Data 1 file.

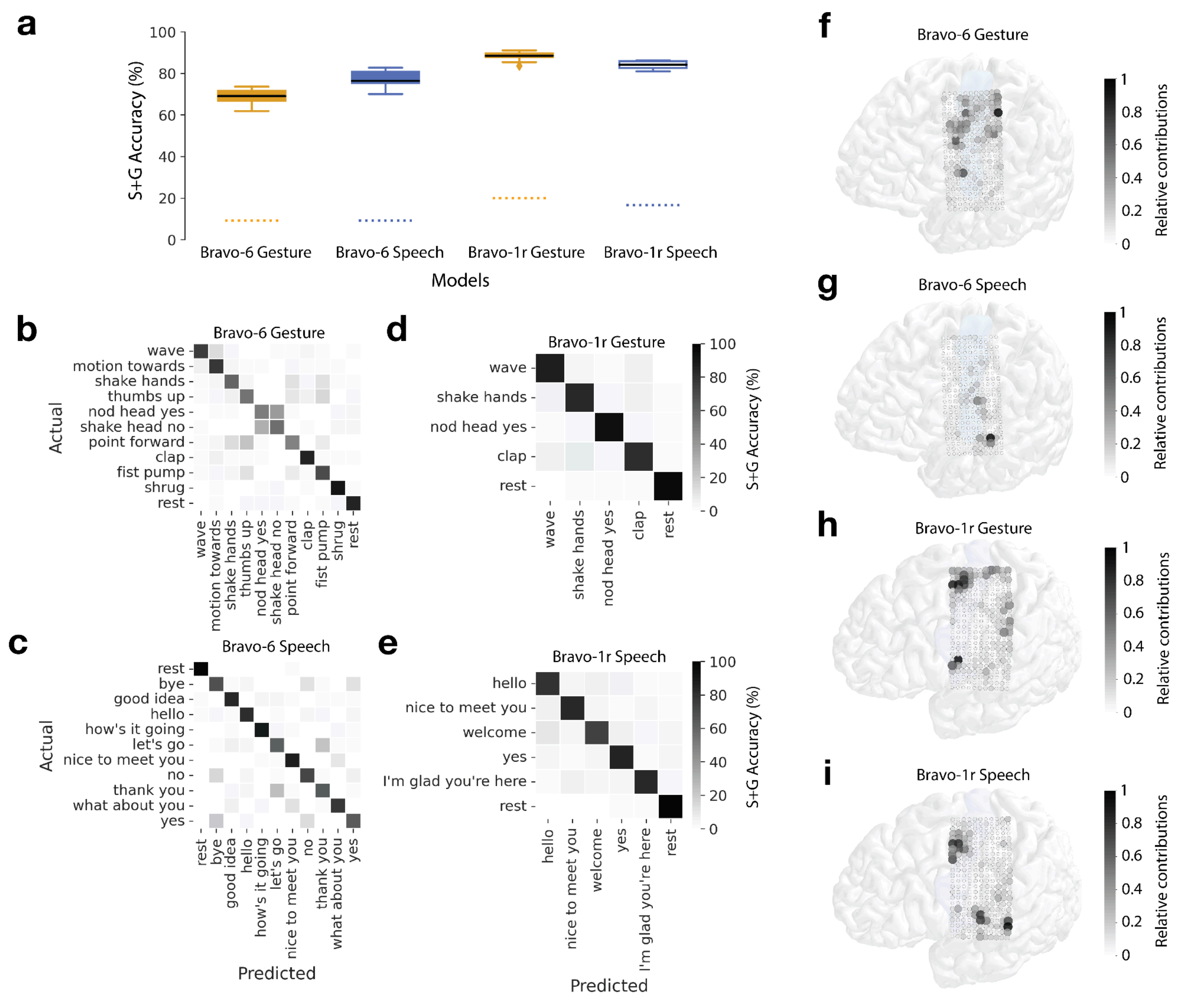

Figure S13. Offline simultaneous decoding results.

(a) Best offline classification accuracy on held-out S+G trials for speech and gesture decoders in participant Bravo-6 and Bravo-1r, trained on all of the available isolated and S+G data. The rest class for each model was trained using the cross-modality training strategy. Boxplots display accuracy across n = 10 test folds. Boxplots show the median (center line), the 25th and 75th percentiles (box limits), and the whiskers extend to the minimum and maximum values within 1.5 times the interquartile range. Dotted lines indicate chance level for each model (set by the number of possible output classes). (b-e) Confusion matrices for S+G decoding, showing that individual gesture and phrase classes are decoded well above chance for both participants. (f-i) Relative electrode contributions for the gesture and speech decoders in each participant during S+G decoding. Contributions maps highlight electrodes contributing most strongly to each decoder's predictions.

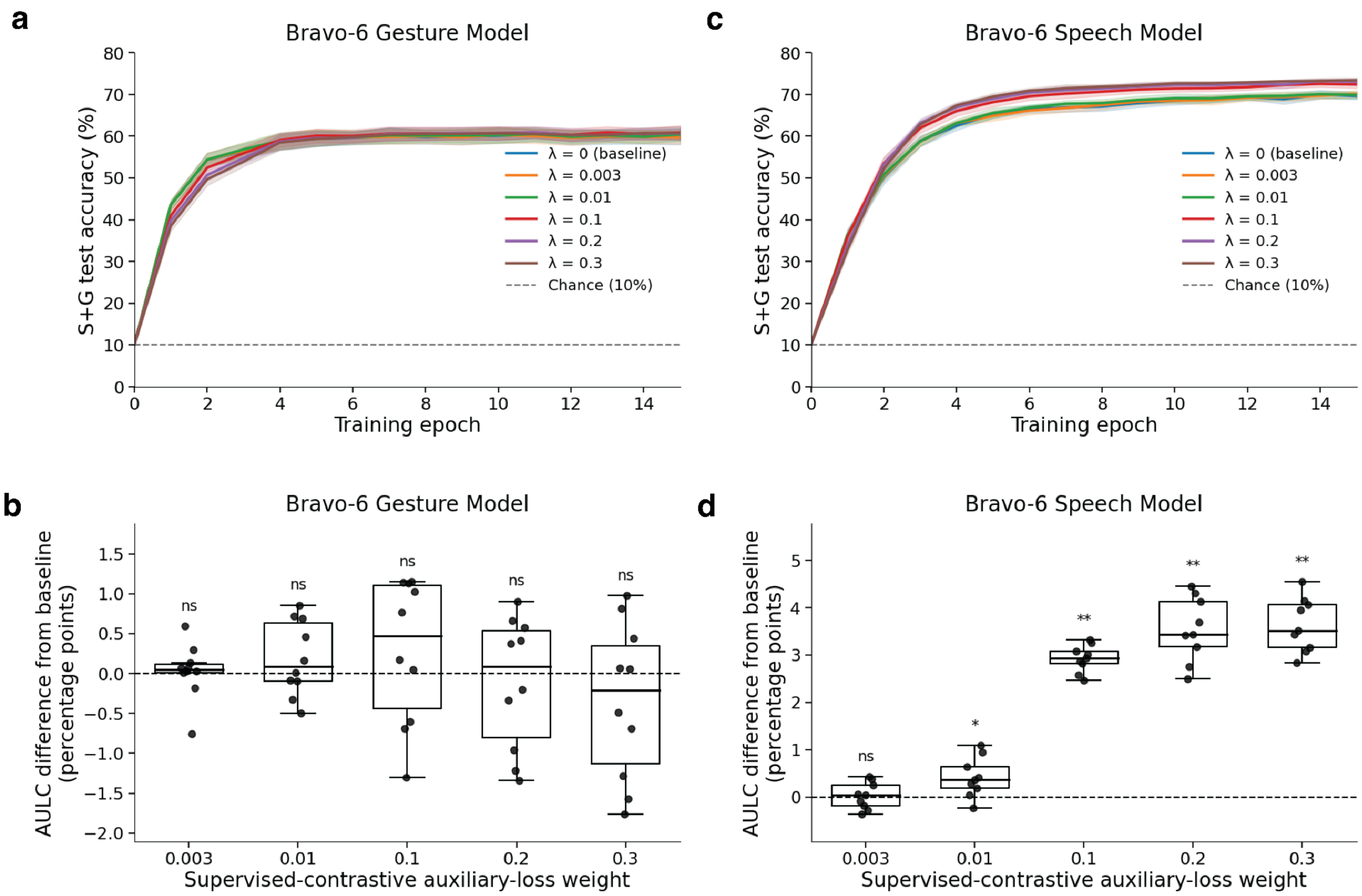

Figure S14. Supervised contrastive loss improves speech model performance on simultaneous speech-and-gesture trials for Bravo-6.

(a,c) S+G test accuracy across training epochs for the gesture (a) and speech (c) decoders trained with cross-entropy loss alone (λ = 0) or with different supervised-contrastive auxiliary-loss weights. Lines show means across outer folds after averaging across inner-fold models, shaded regions indicate 95% confidence intervals across outer folds, and dashed lines indicate chance accuracy (10%). (b,d) Difference in early normalized area under the learning curve (AULC; epochs 0–15) relative to the λ = 0 baseline for the gesture (b) and speech (d) decoders. Though there were not significant improvements to the gesture model, speech model performance (via AULC) improved up to 3.28 percentage points. Each point represents the difference between a given auxiliary-loss weight and the λ = 0 baseline within the same outer test fold (n = 10 outer folds). Boxplots show the median (center line), the 25th and 75th percentiles (box limits), and the whiskers extend to the minimum and maximum values within 1.5 times the interquartile range. Statistical comparisons were performed using two-sided Wilcoxon signed-rank tests with Benjamini-Hochberg FDR correction across auxiliary-loss weights within each decoder. * P < 0.05, ** P < 0.01; ns, not significant. Exact P-values for all comparisons are provided in the Supplementary Data 1 file.

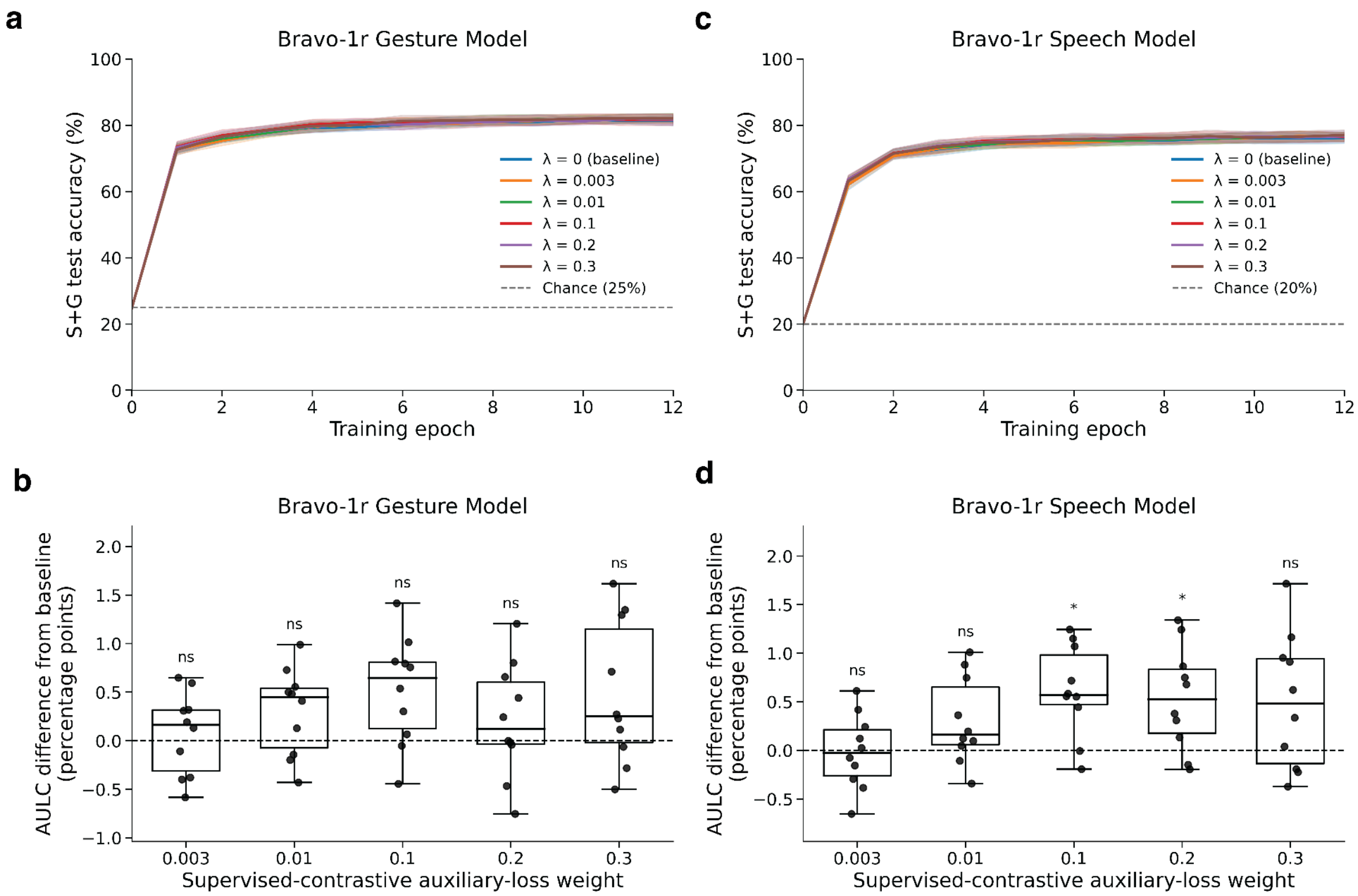

Figure S15. Supervised contrastive loss improves speech model performance on simultaneous speech-and-gesture trials for Bravo-1r.

(a,c) S+G test accuracy across training epochs for the gesture (a) and speech (c) decoders trained with cross-entropy loss alone (λ = 0) or with different supervised-contrastive auxiliary-loss weights. Lines show means across outer folds after averaging across inner-fold models, shaded regions indicate 95% confidence intervals across outer folds, and dashed lines indicate chance accuracy (chance = 25% for gesture model, 20% for speech model). (b,d) Difference in early normalized area under the learning curve (AULC; epochs 0–12) relative to the λ = 0 baseline for the gesture (b) and speech (d) decoders. Though there were not significant improvements to the gesture model, speech model performance (via AULC) had small improvements at λ = 0.1 and λ = 0.2, by 0.61 and 0.54 percentage points. Each point represents the difference between a given auxiliary-loss weight and the λ = 0 baseline within the same outer test fold (n = 10 outer folds). Boxplots show the median (center line), the 25th and 75th percentiles (box limits), and the whiskers extend to the minimum and maximum values within 1.5 times the interquartile range. Statistical comparisons were performed using two-sided Wilcoxon signed-rank tests with Benjamini-Hochberg FDR correction across auxiliary-loss weights within each decoder. * $P < 0.05$, ** $P < 0.01$; ns, not significant. Exact P-values for all comparisons are provided in the Supplementary Data 1 file.

Supplementary Tables

Table S1. Statistical comparisons for Figure 3a-d.

Panel	Participant	Condition 1	Condition 2	Mann-Whitney U	FDR Corrected P-value
a	Bravo-6	Train G-only; Test S+G	Train S+G-only; Test S+G	0	0.000406428
a	Bravo-6	Train G-only; Test S+G	Train Both; Test S+G	0	0.000406428
a	Bravo-6	Train S+G-only; Test S+G	Train Both; Test S+G	12	0.005857526
a	Bravo-6	Train G-only; Test G-only	Train S+G-only; Test G-only	95.5	0.000972061
a	Bravo-6	Train G-only; Test G-only	Train Both; Test G-only	18	0.019273305
a	Bravo-6	Train S+G-only; Test G-only	Train Both; Test G-only	0	0.000406428
a	Bravo-6	Train G-only; Test S+G	Train G-only; Test G-only	0	0.000406428
a	Bravo-6	Train S+G-only; Test S+G	Train S+G-only; Test G-only	99	0.000438276
a	Bravo-6	Train Both; Test S+G	Train Both; Test G-only	60	0.472342387
b	Bravo-1r	Train G-only; Test S+G	Train S+G-only; Test S+G	0	0.000367451
b	Bravo-1r	Train G-only; Test S+G	Train Both; Test S+G	0	0.000367451
b	Bravo-1r	Train S+G-only; Test S+G	Train Both; Test S+G	25.5	0.07811157
b	Bravo-1r	Train G-only; Test G-only	Train S+G-only; Test G-only	100	0.000367451
b	Bravo-1r	Train G-only; Test G-only	Train Both; Test G-only	56	0.673861216
b	Bravo-1r	Train S+G-only; Test G-only	Train Both; Test G-only	0.5	0.000367451
b	Bravo-1r	Train G-only; Test S+G	Train G-only; Test G-only	0	0.000367451
b	Bravo-1r	Train S+G-only; Test S+G	Train S+G-only; Test G-only	90	0.003595168
b	Bravo-1r	Train Both; Test S+G	Train Both; Test G-only	9	0.003252774
c	Bravo-6	Train S-only; Test S+G	Train S+G-only; Test S+G	4.5	0.000725605
c	Bravo-6	Train S-only; Test S+G	Train Both; Test S+G	0	0.000267922
c	Bravo-6	Train S+G-only; Test S+G	Train Both; Test S+G	0	0.000267922

c	Bravo-6	Train S-only; Test S-only	Train S+G-only; Test S-only	100	0.000267922
c	Bravo-6	Train S-only; Test S-only	Train Both; Test S-only	4	0.000724598
c	Bravo-6	Train S+G-only; Test S-only	Train Both; Test S-only	0	0.000267922
c	Bravo-6	Train S-only; Test S+G	Train S-only; Test S-only	0	0.000267922
c	Bravo-6	Train S+G-only; Test S+G	Train S+G-only; Test S-only	26	0.073902629
c	Bravo-6	Train Both; Test S+G	Train Both; Test S-only	0	0.000267922
d	Bravo-1r	Train S-only; Test S+G	Train S+G-only; Test S+G	3	0.000562442
d	Bravo-1r	Train S-only; Test S+G	Train Both; Test S+G	0	0.000274008
d	Bravo-1r	Train S+G-only; Test S+G	Train Both; Test S+G	0	0.000274008
d	Bravo-1r	Train S-only; Test S-only	Train S+G-only; Test S-only	100	0.000274008
d	Bravo-1r	Train S-only; Test S-only	Train Both; Test S-only	41	0.519579413
d	Bravo-1r	Train S+G-only; Test S-only	Train Both; Test S-only	0	0.000274008
d	Bravo-1r	Train S-only; Test S+G	Train S-only; Test S-only	0	0.000274008
d	Bravo-1r	Train S+G-only; Test S+G	Train S+G-only; Test S-only	81	0.023612269
d	Bravo-1r	Train Both; Test S+G	Train Both; Test S-only	0	0.000274008

Table S2. Statistical comparisons for Figure 4b-c.

Panel	Participant	Condition 1 (training strategy; test)	Condition 2 (training strategy; test)	Mann-Whitney U	FDR Corrected P-value
b	Bravo-6	Rest-only; test on rest	Cross-trained; test on rest	190	0.776337942
b	Bravo-6	Rest-only; test on other	Cross-trained; test on other	330	0.001449319
b	Bravo-6	Rest-only; test on rest	Rest-only; test on other	90	0.005464956
b	Bravo-6	Cross-trained; test on rest	Cross-trained; test on other	218	0.776337942
b	Bravo-1r	Rest-only; test on rest	Cross-trained; test on rest	188	0.754524762
b	Bravo-1r	Rest-only; test on other	Cross-trained; test on other	400	1.32688E-07
b	Bravo-1r	Rest-only; test on rest	Rest-only; test on other	0	1.32688E-07
b	Bravo-1r	Cross-trained; test on rest	Cross-trained; test on other	279	0.038150719
c	Bravo-6	Train isolated; test on isolated	Train S+G; test on isolated	16.5	9.52019E-07
c	Bravo-6	Train isolated; test on isolated	Train both; test on isolated	168.5	0.413053582
c	Bravo-6	Train S+G; test on isolated	Train both; test on isolated	384.5	9.52019E-07
c	Bravo-6	Train isolated; test on S+G	Train S+G; test on S+G	390	7.57557E-07
c	Bravo-6	Train isolated; test on S+G	Train both; test on S+G	398.5	3.92152E-07
c	Bravo-6	Train S+G; test on S+G	Train both; test on S+G	232.5	0.413053582
c	Bravo-6	Train isolated; test on isolated	Train isolated; test on S+G	4	3.92152E-07
c	Bravo-6	Train S+G; test on isolated	Train S+G; test on S+G	377	2.3495E-06
c	Bravo-6	Train both; test on isolated	Train both; test on S+G	199.5	1
c	Bravo-1r	Train isolated; test on isolated	Train S+G; test on isolated	56.5	0.000157151
c	Bravo-1r	Train isolated; test on isolated	Train both; test on isolated	208.5	0.824168269
c	Bravo-1r	Train S+G; test on isolated	Train both; test on isolated	350	9.71874E-05
c	Bravo-1r	Train isolated; test on S+G	Train S+G; test on S+G	400	2.03294E-07
c	Bravo-1r	Train isolated; test on S+G	Train both; test on S+G	400	2.03294E-07

c	Bravo-1r	Train S+G; test on S+G	Train both; test on S+G	178	0.630606293
c	Bravo-1r	Train isolated; test on isolated	Train isolated; test on S+G	0	2.03294E-07
c	Bravo-1r	Train S+G; test on isolated	Train S+G; test on S+G	269	0.081986994
c	Bravo-1r	Train both; test on isolated	Train both; test on S+G	51	9.71874E-05

Table S3. Bravo-1r utterance sets.

Speech-only	Gesture-only (set of 4)	Gesture-only (set of 10)	Speech + Gestures
hello	wave	wave	hello + wave
nice to meet you	shake hands	motion towards	hello + shake hands
welcome	clap	clap	nice to meet you + wave
I’m glad you’re here	nod head yes	shrug	nice to meet you + shake hands
yes		nod head yes	welcome + wave
		shake head no	welcome + shake hands
		point forward	welcome + clap
		fist pump	welcome + nod head yes
		thumbs up	I’m glad you’re here + clap
		shake hands	I’m glad you’re here + nod head yes
		wave	yes + clap
		motion towards	yes + nod head yes

Table S4. Bravo-6 utterance sets.

Speech-only	Gesture-only	Speech + gestures
bye	wave	bye + wave
good idea	motion towards	bye + motion towards
hello	clap	bye + clap
how’s it going	shrug	bye + shrug
let’s go	nod head yes	bye + nod head yes
nice to meet you	shake head no	bye + shake head no
no	point forward	bye + point forward
thank you	fist pump	bye + fist pump
what about you	thumbs up	bye + thumbs up
yes	shake hands	bye + shake hands
		good idea + wave
		good idea + motion towards
		good idea + clap
		good idea + shrug
		good idea + nod head yes
		good idea + shake head no
		good idea + point forward
		good idea + fist pump
		good idea + thumbs up
		good idea + shake hands
		hello + wave
		hello + motion towards
		hello + clap
		hello + shrug
		hello + nod head yes
		hello + shake head no
		hello + point forward
		hello + fist pump
		hello + thumbs up

		hello + shake hands
		how’s it going + wave
		how’s it going + motion towards
		how’s it going + clap
		how’s it going + shrug
		how’s it going + nod head yes
		how’s it going + shake head no
		how’s it going + point forward
		how’s it going + fist pump
		how’s it going + thumbs up
		how’s it going + shake hands
		let’s go + wave
		let’s go + motion towards
		let’s go + clap
		let’s go + shrug
		let’s go + nod head yes
		let’s go + shake head no
		let’s go + point forward
		let’s go + fist pump
		let’s go + thumbs up
		let’s go + shake hands
		nice to meet you + wave
		nice to meet you + motion towards
		nice to meet you + clap
		nice to meet you + shrug
		nice to meet you + nod head yes
		nice to meet you + shake head no
		nice to meet you + point forward
		nice to meet you + fist pump
		nice to meet you + thumbs up
		nice to meet you + shake hands

		no + wave
		no + motion towards
		no + clap
		no + shrug
		no + nod head yes
		no + shake head no
		no + point forward
		no + fist pump
		no + thumbs up
		no + shake hands
		what about you + wave
		what about you + motion towards
		what about you + clap
		what about you + shrug
		what about you + nod head yes
		what about you + shake head no
		what about you + point forward
		what about you + fist pump
		what about you + thumbs up
		what about you + shake hands
		yes + wave
		yes + motion towards
		yes + clap
		yes + shrug
		yes + nod head yes
		yes + shake head no
		yes + point forward
		yes + fist pump
		yes + thumbs up
		yes + shake hands

Table S5. Naturalness classifications for simultaneous speech–gesture combinations used with Bravo-6.

Text label	Natural or Unnatural	Justification
"bye" + wave	natural	A wave is a common nonverbal accompaniment to saying goodbye.
"bye" + motion towards	unnatural	A motion toward the listener suggests approach or invitation, which conflicts with saying goodbye.
"bye" + clap	unnatural	Clapping is celebratory and does not normally accompany a goodbye.
"bye" + shrug	unnatural	A shrug conveys uncertainty or indifference, which is not a typical goodbye gesture.
"bye" + nod head yes	natural	A nod can function as a brief social acknowledgment while saying goodbye.
"bye" + shake head no	unnatural	Shaking the head no conflicts with the social meaning of saying goodbye.
"bye" + point forward	unnatural	Pointing forward does not clearly support or modify a goodbye.
"bye" + fist pump	unnatural	A fist pump is celebratory and is not typically paired with saying goodbye.
"bye" + thumbs up	natural	A thumbs up can serve as a positive closing signal while saying goodbye.
"bye" + shake hands	natural	A handshake is a common closing gesture when parting from someone.
"good idea" + wave	unnatural	A wave is mainly a greeting or farewell and does not add clear meaning to 'good idea'.
"good idea" + motion towards	unnatural	A motion toward the listener is ambiguous and does not clearly support 'good idea'.
"good idea" + clap	natural	Clapping can show approval or enthusiasm for an idea.
"good idea" + shrug	unnatural	A shrug conveys uncertainty, which weakens or conflicts with the phrase 'good idea'.
"good idea" + nod head yes	natural	Nodding yes reinforces agreement with the phrase 'good idea'.
"good idea" + shake head no	unnatural	Shaking the head no contradicts the positive meaning of 'good idea'.
"good idea" + point forward	natural	Pointing toward the listener can plausibly indicate that their suggestion is the good idea.
"good idea" + fist pump	natural	A fist pump can express enthusiasm or approval for an idea.
"good idea" + thumbs up	natural	A thumbs up directly reinforces approval of the idea.
"good idea" + shake hands	natural	A handshake can signal agreement or acceptance of a good idea.
"hello" + wave	natural	A wave is a common greeting gesture.
"hello" + motion towards	natural	A motion toward the listener can function as an inviting greeting.
"hello" + clap	unnatural	Clapping is not usually used as a simple greeting in conversation.

"hello" + shrug	unnatural	A shrug conveys uncertainty and does not naturally accompany 'hello'.
"hello" + nod head yes	natural	A nod can function as a brief greeting or acknowledgment.
"hello" + shake head no	unnatural	Shaking the head no conflicts with the social intent of greeting someone.
"hello" + point forward	natural	Pointing forward can direct the greeting toward the listener.
"hello" + fist pump	unnatural	A fist pump is celebratory and is not a typical greeting.
"hello" + thumbs up	natural	A thumbs up can serve as a friendly positive greeting.
"hello" + shake hands	natural	A handshake is a common greeting gesture.
"how's it going" + wave	natural	A wave commonly accompanies a casual greeting such as 'how's it going'.
"how's it going" + motion towards	natural	A motion toward the listener can invite them into the interaction while asking the question.
"how's it going" + clap	unnatural	Clapping does not naturally support a greeting question.
"how's it going" + shrug	natural	A shrug can add a casual or open-ended tone to the question.
"how's it going" + nod head yes	natural	A nod can encourage the listener to respond to the question.
"how's it going" + shake head no	unnatural	Shaking the head no does not naturally fit an open greeting question.
"how's it going" + point forward	natural	Pointing forward can direct the question to the listener.
"how's it going" + fist pump	unnatural	A fist pump is celebratory and does not fit a general greeting question.
"how's it going" + thumbs up	natural	A thumbs up can add a friendly or positive tone to the greeting.
"how's it going" + shake hands	natural	A handshake can accompany a greeting such as 'how's it going'.
"let's go" + wave	natural	A wave can cue someone to come along or move with the speaker.
"let's go" + motion towards	natural	A motion toward the listener directly supports an invitation to go together.
"let's go" + clap	natural	Clapping can add encouragement or urgency to 'let's go'.
"let's go" + shrug	unnatural	A shrug conveys uncertainty, which conflicts with the directive 'let's go'.
"let's go" + nod head yes	natural	Nodding yes reinforces agreement or readiness to go.
"let's go" + shake head no	unnatural	Shaking the head no contradicts the phrase 'let's go'.
"let's go" + point forward	natural	Pointing forward can indicate direction while saying 'let's go'.
"let's go" + fist pump	natural	A fist pump can express excitement or motivation while saying 'let's go'.

"let's go" + thumbs up	natural	A thumbs up can signal agreement or readiness.
"let's go" + shake hands	unnatural	A handshake usually signals greeting, closing, or agreement rather than moving together.
"nice to meet you" + wave	natural	A wave can accompany a polite greeting or introduction.
"nice to meet you" + motion towards	unnatural	A motion toward the listener is less conventional than a greeting gesture for this phrase.
"nice to meet you" + clap	unnatural	Clapping is not typically used when saying 'nice to meet you'.
"nice to meet you" + shrug	unnatural	A shrug conveys uncertainty and conflicts with the polite greeting.
"nice to meet you" + nod head yes	natural	A nod is a common polite acknowledgment during an introduction.
"nice to meet you" + shake head no	unnatural	Shaking the head no conflicts with the positive meaning of the phrase.
"nice to meet you" + point forward	unnatural	Pointing forward is not a typical polite gesture for an introduction.
"nice to meet you" + fist pump	unnatural	A fist pump is celebratory and does not match a polite introduction.
"nice to meet you" + thumbs up	natural	A thumbs up can add a friendly positive tone to the greeting.
"nice to meet you" + shake hands	natural	A handshake is a standard gesture when meeting someone.
"no" + wave	unnatural	A simple wave usually signals greeting or farewell rather than refusal.
"no" + motion towards	unnatural	A motion toward the listener suggests invitation or approach, which conflicts with 'no'.
"no" + clap	unnatural	Clapping is positive or celebratory and does not naturally accompany 'no'.
"no" + shrug	natural	A shrug can accompany a negative or uncertain response.
"no" + nod head yes	unnatural	Nodding yes directly contradicts the spoken response 'no'.
"no" + shake head no	natural	Shaking the head no directly reinforces the spoken response.
"no" + point forward	unnatural	Pointing forward does not clearly support the negative response.
"no" + fist pump	unnatural	A fist pump is celebratory and conflicts with a negative response.
"no" + thumbs up	unnatural	A thumbs up conveys approval and conflicts with 'no'.
"no" + shake hands	unnatural	A handshake suggests agreement or greeting and does not naturally accompany 'no'.
"thank you" + wave	natural	A wave can accompany thanks, especially when closing an interaction.
"thank you" + motion towards	unnatural	A motion toward the listener does not clearly support the expression of thanks.
"thank you" + clap	natural	Clapping can express appreciation or thanks.

"thank you" + shrug	unnatural	A shrug conveys uncertainty or indifference, which weakens the expression of thanks.
"thank you" + nod head yes	natural	A nod can reinforce acknowledgment or gratitude.
"thank you" + shake head no	unnatural	Shaking the head no conflicts with the positive expression of thanks.
"thank you" + point forward	natural	Pointing forward can direct the thanks toward the listener.
"thank you" + fist pump	unnatural	A fist pump is celebratory and is not a typical gesture for gratitude.
"thank you" + thumbs up	natural	A thumbs up can signal appreciation or approval.
"thank you" + shake hands	natural	A handshake can accompany thanks in a social interaction.
"what about you?" + wave	unnatural	A wave is usually a greeting or farewell and does not clearly direct the question.
"what about you?" + motion towards	natural	A motion toward the listener can invite them to respond.
"what about you?" + clap	unnatural	Clapping does not naturally support asking the listener a question.
"what about you?" + shrug	natural	A shrug can signal uncertainty or openness while asking the question.
"what about you?" + nod head yes	unnatural	Nodding yes does not clearly add meaning to the question.
"what about you?" + shake head no	unnatural	Shaking the head no conflicts with or distracts from the open question.
"what about you?" + point forward	natural	Pointing forward directly indicates that the question is directed to the listener.
"what about you?" + fist pump	unnatural	A fist pump is celebratory and does not fit an open question.
"what about you?" + thumbs up	unnatural	A thumbs up conveys approval rather than asking the listener for their response.
"what about you?" + shake hands	unnatural	A handshake does not naturally support asking 'what about you?'.
"yes" + wave	unnatural	A wave usually signals greeting or farewell rather than agreement.
"yes" + motion towards	natural	A motion toward the listener can support an affirmative invitation or acceptance.
"yes" + clap	natural	Clapping can express approval or excitement about an affirmative response.
"yes" + shrug	natural	A shrug can soften an affirmative response or convey uncertainty while still answering yes.
"yes" + nod head yes	natural	Nodding yes directly reinforces the spoken response.
"yes" + shake head no	unnatural	Shaking the head no directly contradicts the spoken response 'yes'.
"yes" + point forward	natural	Pointing forward can direct the affirmative response toward the listener or indicate agreement with them.

"yes" + fist pump	natural	A fist pump can express enthusiasm or celebration with 'yes'.
"yes" + thumbs up	natural	A thumbs up directly reinforces agreement or approval.
"yes" + shake hands	natural	A handshake can signal agreement or acceptance.

Supplementary Videos

Video S1. Real-time decoding of gestures during the copy task with Bravo-6.

This video shows an online demonstration of gesture decoding with Bravo-6. The participant is shown a target gesture as text at the top of the screen, which counts down to a go-cue. At the go-cue, the participant covertly attempts the target gesture. The neural activity during his attempt is passed to the gesture model which predicts the gesture and is subsequently used to animate the personalized avatar. The gesture model also predicts rest if no gesture was attempted.

Video S2. Real-time decoding of simultaneous speech and gestures during the copy task with Bravo-6.

This video shows an online demonstration of parallel, simultaneous speech and gesture decoding with Bravo-6. Similar to Video S1, the participant is shown a target speech, target gesture, or target speech and gesture combination to attempt at the go-cue. Bravo-6 overtly attempts speech targets and covertly attempts the gesture targets. The neural activity during his attempts are passed in parallel to a speech model and to a gesture model. Predicted speech is shown at the bottom of the screen as text while the predicted gesture animates the avatar. Both speech and gesture models can predict “rest” if no speech and/or no gesture was attempted, allowing the system to predict during isolated speech attempts, isolated gesture attempts, and simultaneous speech and gesture attempts. This framework enables flexibility when speech and gestures are attempted in isolation or simultaneously.

Video S3. Real-time decoding of speech and gestures in conversation with Bravo-6.

In this video, Bravo-6 uses the speech and gesture decoding framework as shown to respond to a conversational partner speaking to him. A go-cue countdown appears at the bottom of the screen, where Bravo-6 can attempt a response with a speech and/or gesture attempt. Predicted speech is shown at the bottom of the screen as text while the predicted gesture animates the avatar. If no speech was predicted, no response is shown at the bottom of the screen and if no gesture was predicted, the avatar remains idle.

Video S4. Real-time decoding of speech and gestures in conversation with Bravo-1r.

This video shows a similar demonstration as in Video S3, but with Bravo-1r.

Video S5. Real-time decoding of speech and gestures in conversation with a second virtual avatar with Bravo-6.

This video shows a similar demonstration as in Video S3, but with a second virtual avatar as the conversational partner. Similar to Video S3, Bravo-6 uses the speech and gesture decoding framework to respond to a conversational partner. The conversational partner is a second virtual avatar (named Lexi) animated by various gestures. Dialogue from the second avatar is shown in a chat log on the left side of the screen. The second avatar also gestures, and various gestures are also noted in the chat log. Go-cue countdowns for Bravo-6, at which time he can attempt a

response, are shown at the bottom right of the screen. Bravo-6’s decoded speech is shown as text in the chat log (with the name Oskar). Decoded gestures animate his personalized avatar, and are also noted in the chat log. This video demonstrates a proof-of-concept framework for virtual communication.

CLINICAL PROTOCOL

(updated 11/19/2024)

Title:
A High-Performance ECoG-based Neural Interface for Communication and Neuroprosthetic Control

Protocol:
FDA reference number: G170242
UCSF-BMI-ECoG-2022-06

Study Sponsors/Investigators:

Redacted

Protocol Synopsis

**Title**	**A High-Performance ECoG-based Neural Interface for Communication and Neuroprosthetic Control**
**Study Phase**	Phase I
**Device(s)**	**Device Information:** Devices to be used in this study are grouped below according to FDA approval.<br><br>**Cleared for temporary (<30 days) recording and monitoring of brain electrical activity under 510k:**<br>● **NeuroPort Array, PN 6248 (K070272, K110010)**<br><br>**Seeking Special 510(k) (not yet submitted to FDA):**<br>● **Digital NeuroPort System (Modification to existing NeuroPort System - K042626)**<br><br>**Cleared for temporary (<30 day) use with recording, monitoring, and stimulation equipment for the recording, monitoring and stimulation of electrical signals on the surface of the brain under 510k:**<br>● **PMT Subdural Cortical Electrodes, Model #2110TX-253-001 (K082474)**<br>● PMT Subdural Cortical Electrodes, Model #2110TX-128-005 (K082474)<br><br>**Not cleared**:<br>• Blackrock Microsystems 256-channel pedestal connector (see Appendix E for justification of use)<br>• Blackrock Microsystems CerePlex E256 digital head stage<br><br>The Blackrock Microsystems NeuroPort Array connector pedestal, a subcomponent of the Blackrock Microsystems NeuroPort Array, or the 256-channel Blackrock Microsystems connector pedestal will be laser bonded to the PMT Subdural Cortical Electrode by Blackrock Microsystems. As documented below, the combination device including the Blackrock Microsystems NeuroPort Array connector pedestal laser bonded to the PMT Subdural Cortical Electrode has already been tested in non-human primates (over at least an 18-month period, see below regarding published report).<br><br>**Request for off-label use of the combined investigational device for 1 year, for the equivalent indication of recording and monitoring of brain electrical activity.**

**Indication**	Adults with neurological disorders (e.g. amyotrophic lateral sclerosis/ALS, spinal cord injury, multiple sclerosis, stroke) often develop disorders of movement and communication. We aim to determine the feasibility of ECoG based brain computer interface control of complex neuroprosthetic devices.
**Sponsor Contacts**	*Redacted*
**Data Safety Monitor Board (DSMB)**	*Redacted*
**Treatment**	The Blackrock Microsystems NeuroPort Array connector pedestal, NeuroPort system, and PMT Subdural Cortical Electrodes are currently cleared for monitoring of patients for up to 30 days. The Blackrock Microsystems 256-channel NeuroPort connector pedestal and CerePlex E256 digital head stage are not cleared by the FDA. Blackrock has modifications to the NeuroPort system for which they are seeking special 510(k) clearance. Here we to aim to use these systems combined for at least a 1-year period in subjects with neurological illness and disorders of communication to test feasibility for both neuroprosthetic control and for decoding speech from neural activity.

**Study Sites**	*Redacted*

**Study Design**	This is a **single-center early feasibility study** of the use of an ECoG-based neural interface for testing the feasibility of using ECoG signals to control complex devices for motor and speech control in adults affected by neurological disorders of movement.<br><br>A PMT Subdural Cortical Electrode array, bonded to the Blackrock Microsystems NeuroPort Array pedestal or to the 256-channel NeuroPort pedestal, will be surgically placed directly on the brain surface over the motor and language cortices of subjects with disorders of motor control. After implantation of the electrode and pedestal, the NeuroPort Biopotential Processing System will be connected to the NeuroPort Array pedestal, or to the 256-channel NeuroPort pedestal, to monitor and record neural signals. With this ECoG-based neural interface, study patients will undergo training and assessment of their ability to control assistive robotic arms and to determine if ECoG brain signals can be decoded for language communication. This will be performed in two phases.<br><br><u>Phase 1: Optimize BCI system</u><br>In Phase 1, we will optimize the entire system to reliably detect neural activity to ensure that the recorded signals are stable and free of artifacts. Moreover, we will ensure that the neural signals are converted in real-time into cursor movements. During this phase, we will primarily examine cursor based control, decoding of parameters and 'disembodied' control (i.e. the subjects' arm will not interact with the mechanical system). This phase will be conducted in the outpatient office setting and/or the patient's home environment, based on patient preference and needs. We anticipate that this phase will take approximately 1 month; however, this may be longer or shorter for each subject depending on the level of control achieved.<br><br><u>Phase 2: Testing of BCI Control</u><br>In Phase 2, we will test feasibility for both neuroprosthetic control and for decoding speech from neural activity. We will begin to perform experimental testing with the system for control of an assistive robotic arms that meets criteria for a non-significant risk device. As outlined in the sections below, we have multiple safety features to ensure that there is minimal risk for injury during use of the robotic arms, including subject contact during object interaction and the embodiment phase (i.e. the subjects arm is interacting with the exoskeleton). Throughout this period, neural signals will be recorded and analyzed, and tasks will be performed toward the development, assessment, and improvement of the neural interface system. We will assess quality of performance using kinematic parameters while performing required tasks. We will analyze stability of neural recordings and performance. To measure stability of the neural representation we will analyze the neural correlates of imagined movements. We will also assess changes in spatial correlation scales and other redundancy measures during learning and stable performance. We anticipate that testing will be conducted in the outpatient office or home setting based on the patient's preference and needs.

	In this phase, we will also continue to perform experimental testing with the system for control of a virtual communicating interface. Throughout this period, neural signals will be recorded and analyzed, and tasks will be performed toward the development, assessment, and improvement of the communication interface.

**Objectives**	In eight patients with severe disorders of movement control, we will surgically implant PMT Subdural Cortical Electrodes on the brain surface over the motor and language cortices. The electrode will be bonded to a Blackrock NeuroPort Array pedestal or the Blackrock 256-channel NeuroPort pedestal, which can be connected to the Digital NeuroPort system in order to process and record neural activity in real time. Using this neural interface, we will condition the patient to be able to control assistive robotic arms, communicate with a computer system for typing and perform speech output tasks. We will utilize optimal neural plasticity mechanisms, a novel decoder framework, and advanced language modeling during BCI conditioning.<br><br>Hypothesis: The underlying hypothesis is that ECoG recordings will allow severely paralyzed individuals to skillfully control complex neuroprosthetic devices for movement and communication. A closely related hypothesis is that the well-known stability of ECoG signals will allow us to maximally engage neural mechanism of plasticity and thereby optimize long-term skilled acquisition.

**Patient Population**	Study subjects will be adults with severe motor impairment secondary to a neurological disorder.<br><br><u>Inclusion Criteria</u><br>● Age 18-75 years old<br>● Limited ability to use upper limbs and limited ability to use speech, based on neurological examination by a board-certified neurological specialist, due to stroke, spastic quadriplegic cerebral palsy (with no significant cognitive deficits based on formal neuropsychological testing), amyotrophic lateral sclerosis (ALS), multiple sclerosis, cervical spinal cord injury, brainstem stroke, muscular dystrophy, myopathy or severe neuropathy<br>● Disability, defined by a 3 or greater score on the Modified Rankin Scale,must be severe enough to require some assistance, but able to walk without assistance of another person<br>● If stroke or spinal cord injury, at least 1 year has passed since onset of symptoms<br>● Must live within a two-hour drive of UCSF<br><br><u>Exclusion Criteria</u><br>● Pregnancy or breastfeeding<br>● Inability to understand and/or read English<br>● Inability to give consent<br>● Dementia, based on history, physical exam, and MMSE<br>● Active depression (BDI > 20) or other psychiatric illness (active general anxiety disorder, schizophrenia, bipolar disorder, obsessive-compulsive disorder (OCD), or personality disorders (e.g. multiple personality disorder, borderline personality disorder, etc.)<br>● A baseline Columbia Suicide Severity Rating Scale (C-SSRS) with positive suicidal ideation or behavior occurring within 5 years prior to enrollment<br>● Comorbidities including uncontrolled hypertension, cancer, or major organ system failure.<br>● Prior intracranial surgeries that could increase the risk to the study participant or impact the effectiveness of the investigational device<br>● History of substance abuse within the last year<br>● Ongoing anticoagulation which cannot be stopped in the peri-procedural period.<br>● Inability to comply with study follow-up visits<br>● Immunocompromised<br>● Has an active infection<br>● Has a CSF drainage system implanted or revised less than 1 year ago, history of prior CSF drainage system implant infection, presence of concurrent external ventricular drain, an active CSF leak, or other potential risk factors of CSF drainage system infection.<br>● Requires diathermy, electroconvulsive therapy (ECT), or transcranial magnetic stimulation (TMS) to treat a chronic condition

	● Has any type of cranially-implanted generator, such as a cochlear implant or deep brain stimulator<br>● Allergies or known hypersensitivity to materials in the Blackrock NeuroPort Array pedestal or Blackrock 256-channel pedestal (i.e. silicone, titanium) or the PMT Subdural Cortical Electrode (silicone, platinum iridium, nichrome)

**Sample Size**	8 patients
**Efficacy Assessments**	Primary Endpoints: Feasibility of control of assistive robotic arms and a communication interface.
**Safety Assessments**	• Physical examination at all study visits<br>• Perform functional test of the PMT/Blackrock combined neural interface system prior to implantation<br>• Surgical/ or nonsurgical protocol-defined adverse events recorded on adverse events case report forms and use of protocol-defined procedures for adverse event management<br>• Assessment of suicidality using Columbia Suicide Severity Rating Scale, and assessment of changes using the Beck Depression and Anxiety Inventories, at monthly intervals.

Table of Contents

Title Page

i. Protocol Synopsis

ii. Table of Contents

iii. Investigator Agreement / Protocol Signature Page

1. Introduction
2. Clinical Study Outline & Study Objectives
3. Investigational Plan
4. Patient Eligibility
5. Study Device
6. Study Procedure
7. Clinical Measurements and Procedures
8. Data Management
9. Statistical Methods and Data Analysis
10. Regulatory Requirements
11. References
12. Appendices

Investigator Agreement / Protocol Signature Page

Redacted

1.0 Introduction

Restoration of function with assistive robotic arms and a communication interface are important goals of BCIs. Multiple neurological disorders can result in severe bilateral upper limb weakness or paralysis. Surveys of patients with quadriplegia provide important guidance about specific rehabilitation needs and preferences as well as targets for intervention. A survey of patients with tetraplegia resulting from SCI found that restoration of arm and hand function was of highest priority (Anderson 2004). Similarly, patients with ALS reported that direct neural control of a robotic arm would be of great importance (Huggins, Wren and Gruis 2011). While there has been extensive research into each disease process, little has been proven to be clinically effective for the rehabilitation of this chronic disability (Wolpaw et al. 2002).

In patients with conditions such as high cervical SCI, basilar strokes or advanced ALS, the effects can be particularly devastating as tetraplegia may be accompanied by paralysis of oral structures, leading to the loss of voluntary vocal function. Individuals with severe traumatic brain injury or high cervical injuries who require mechanical ventilation may also have limited capacity to communicate (Kennedy 1994, Kubler et al. 2001, Monti et al. 2010). Standard augmentative and alternative communication (AAC) devices are widely available but may not be suitable for individuals with severe or complete paralysis of the voluntary motor system (Selzer et al. 2014). For example, the most commonly used devices are gaze trackers that allow computer cursor control through eye movements (Spataro et al. 2014). However, eye tracking may have a limited role in advanced cases of ALS and after brainstem strokes, where eye movements are frequently affected (Birbaumer et al. 1999, Kennedy and Bakay 1998). They also require sustained visual attention and may create a high cognitive burden. Thus, it remains critical to develop novel technologies to help restore communication as well as upper limb function.

Brain-Computer Interfaces (BCIs) are a very promising method to significantly improve quality of life by restoring upper extremity function and communication ability in patients with motor and speech disabilities. Over the past decade, the rate and sophistication of research involving invasive BCIs have rapidly increased. BMIs have the potential to seamlessly integrate the computational power of artificial electronic systems with that of the nervous system. Multiple neural signals can be used in a BCI, including non-invasive EEG signals and other neural signals such as spikes and electrocorticography (ECoG) that require invasive placement of electrodes. Non-invasive BCIs have been studied for at least two decades. Known limitations of surface EEG recordings are a low 'signal-to-noise' and contamination by muscle activity (Wolpaw et al. 2002). Recent studies suggest that BMIs using invasive recording of neural signals (spikes or ECoG) can allow more rapid intuitive control over complex devices (Carmena et al. 2003, Leuthardt et al. 2009, Leuthardt et al. 2004, Schwartz et al. 2006, Taylor, Tillery and Schwartz 2002). A large basic science and clinical effort is currently devoted to BMIs based on invasive recordings of action potentials. However, it is increasingly clear that there are significant limits on possible translation of spikes based recordings. It does not readily permit reliable long-term recordings (Chestek et al. 2009, Ganguly and Carmena 2009, Gilja et al. 2011, Hochberg et al. 2006).

Electrocorticography (ECoG) offers a promising alternative for BMIs (Pasley et al. 2012, Bouchard et al. 2013, Slutzky et al. 2011, Leuthardt et al. 2006, Ganguly et al. 2009). ECoG has the potential to serve as a robust source of neural signals that can be integrated into a clinically viable device. Specifically, it can be: (1) stably recorded over long-periods of time, (2) possesses sufficient 'signal-to-noise' and (3) is known to be rich in information about neural processes, (4) can allow long-term rapid control of multiple degrees of freedom (DOF). Because ECoG arrays lie on the brain surface, they can be stably monitored for very long periods of time (Nadkarni, Ohno-Machado and Chapman 2011a, Thompson et al. 2014b). This approach has been demonstrated clinically in tens of thousands of patients that have had arrays or stimulating electrodes made of the same materials implanted for years (for indications such as pain, movement disorders, or newer closed loop devices for epilepsy) (Nadkarni et al. 2011a).

A significant limitation of current ECoG studies and models is the use of able-bodied subjects in whom motor and sensory pathways are intact. The overwhelming majority of BMI experiments do not monitor physical movements or EMG signals during online BMI control. In addition, intact sensory feedback would likely facilitate learning BMI control. Disabled patients may not have access to such training signals (Bensmaia and Miller 2014). Moreover, it is well known that there is a major reorganization of sensorimotor areas following injury (e.g. after spinal cord injury) (Canolty et al. 2006, Churchland et al. 2012, Miller et al. 2010, Tort et al. 2010). Thus, it remains quite difficult to predict generalization of ECoG-based neuroprosthetic control to disabled patients. It remains unclear if ECoG based control of complex devices can be achieved in paralyzed patients.

Thus, this proposal aims to test feasibility of controlling assistive robotic arms and a communication interface using invasive electrocorticography (ECoG) in subjects with extensive motor disabilities. There have been two main approaches to BCI control, i.e. control of a motor prosthetic (mBCI) or a communication interface (cBCI) (Bensmaia and Miller 2014, Homer et al. 2013, Nicolelis and Lebedev 2009, Schwartz 2004, Shenoy and Carmena 2014). In general, the broader framework for communication is similar to motor control. In a motor BCI, a participant's intention to move is translated into commands for device control. The components of a motor BMI are (1) recordings of neural activity (2) algorithms to transform the neural activity into control signals, (3) an external device driven by these control signals and (4) feedback regarding the current state. Initial studies in able-bodied non-human primates as well as human subjects (e.g. undergoing epilepsy surgical monitoring) have demonstrated the general feasibility of neuroprosthetic control using ECoG based recordings (Leuthardt et al. 2006, Chao, Nagasaka and Fujii 2010, Schalk et al. 2008).

We propose to conduct an early feasibly (i.e. pilot clinical) trial of ECoG-based BMIs in subjects with motor disability. Preclinical studies have suggested that ECoG based recordings provide a viable alternative for clinical translation of BMI technology; however, it remains unclear if complex control can be achieved in patients with neurological disorders. This study will utilize devices cleared by the FDA for the same indication of recording and monitoring of brain electrical activity, but with off label use of the device for at least 1 year to meet the objectives outlined above. Specifically, we will use the Blackrock NeuroPort Array pedestal, or the Blackrock 256-channel pedestal (not currently cleared by FDA), laser-bonded to PMT Subdural Cortical Electrodes, and

connected to the NeuroPort Biopotential Signal Processing System, to acquire neural data. The digitized data will then be transmitted to a personal computer and decoded to direct motion of assistive robotic arms and a communication interface. This will allow individuals to use ECoG neural activity to control complex neuroprosthetic devices. **Demonstration of reliable prosthetic control using ECoG signals in disabled subjects would represent a major milestone. It would strongly motivate the development of implantable wireless ECoG recording systems that can be integrated with assistive robotic devices.**

The Blackrock Microsystems NeuroPort system and the NeuroPort Array have been successfully used in humans with neurological disorders. Several human studies have demonstrated successful long-term implantation of neuroprosthetic control using the Blackrock Microsystem NeuroPort microelectrode array with the NeuroPort system (Hochberg et al. 2012b, Hochberg et al. 2006, Kim et al. 2008a, Ajiboye et al. 2012, Simeral et al. 2011, Bouton et al. 2016). Thus far, at least six patients with tetraplegia have been implanted and monitored with the Blackrock NeuroPort Array and NeuroPort system from published reports. This human data supports chronic implantation of the devices in human patient populations by showing:

1) Chronic stable recordings from the cortex using the NeuroPort Signal Processing system connected to the Blackrock NeuroPort Array may be maintained for at least 5 years.
2) Extensive experience with the decoding of the neural data in humans using these devices
3) Proof of concept that individuals with tetraplegia resulting from neurological injuries could readily use motor cortex neural signals to control a computer cursor after an injury
4) Lack of significant adverse events associated with the devices observed over years after implantation

In this study, the PMT Subdural Cortical Electrodes will replace the Blackrock Microsystems NeuroPort Array microelectrode array for monitoring recordable electrical brain activity. The NeuroPort Array pedestal, or the Blackrock Microsystems 256-channel pedestal, will be bonded to the PMT Subdural Cortical Electrodes (in place of the Blackrock Microsystems NeuroPort Array electrode) in a custom-ordered device collaboratively built by the Blackrock Microsystems and PMT Corporation and sterilized by PMT Corporation. The feasibility of this approach has been demonstrated **in a non-human primate study (i.e. the PMT ECoG grid was connected to a Blackrock Microsystems pedestal connector to monitor neural signals for ~ 18 months)** (Degenhart et al. 2016). After implantation, the study explored the host-tissue response to the subdural ECoG grid implanted for 666 d, focusing on both cortical tissue health and fibrosis at the implant site, while also validating device performance by examining neural responses to overt reaching movements. They found that cortical thickness and neuronal density were both unaffected by the ECoG array implantation. As expected, the grid itself was found to be encapsulated in a fibrous envelope upon explantation. Despite this encapsulation, robust modulation of ECoG signals in recordings conducted 18 months after implantation were observed.

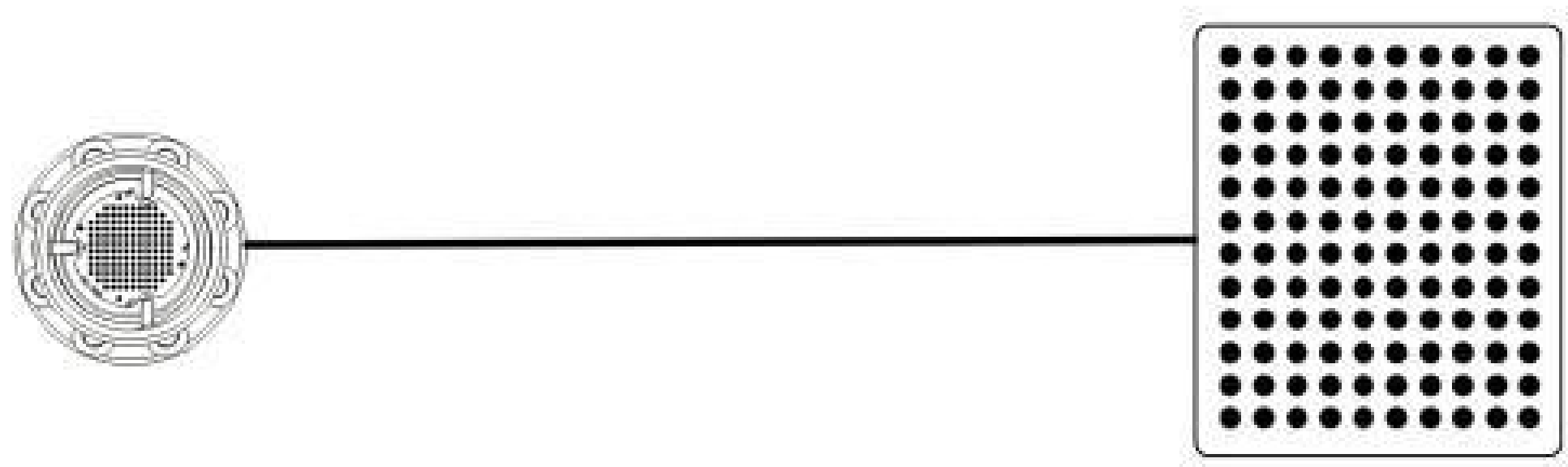

Figure 1: Diagram of pedestal connected to a 128-channel ECoG array

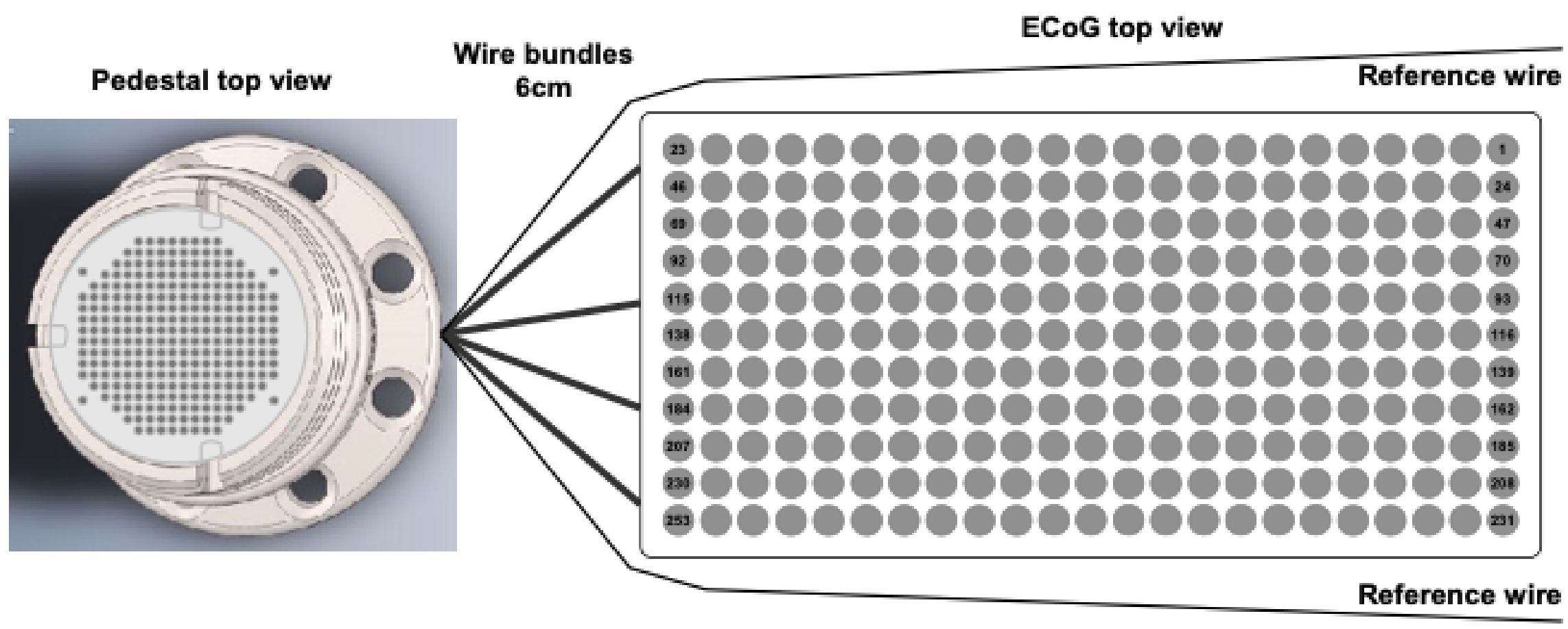

Figure 2: Diagram of pedestal connected to a 253-channel ECoG array

The current literature indicates that ECoG may provide a means by which stable chronic cortical recordings can be obtained with little neural tissue damage, and with a safety level comparable to chronic intracranial device implantations for the treatment of PD. For example, in human subjects, subdural ECoG arrays have been implanted in patients with epilepsy for over 1 year with corroborating evidence of viable long-term neural recordings (Morrell and Group 2011). The primary safety endpoint was achieved whereby the SAE rate for the ECoG system over the first 28 days (device related and not device related) was not worse than the prespecified literature-derived comparator for implantation of intracranial electrodes for seizure localization and epilepsy surgery. Moreover, the SAE rate for the ECoG system during the implant and first 84 days was not worse than the prespecified literature-derived comparator with DBS for PD (Morrell and Group 2011).

2.0 Clinical Study Outline

This study will use the Blackrock Microsystems NeuroPort Biopotential Signal Processing system to record data from the Blackrock Microsystems NeuroPort Array pedestal, or the 256-channel Blackrock Microsystems pedestal, bonded to PMT Subdural Cortical electrodes implanted over cortical areas over a period of at least 1 year. The processed neural signals received by the system will allow us to test the feasibility of training patients to use their neural activity to control assistive robotic arms and a communication interface.

2.1 Study Objectives

This study will allow us to test the feasibility of conditioning individuals with impaired motor ability to be able to control assistive robotic arms and a communication device using ECoG signals.

3.0 Investigational Plan

We aim to conduct a small pilot study to assess the feasibility of translating an ECoG-based interface to control complex neuroprosthetic devices in a severely disabled patient population. **Our underlying hypothesis is that ECoG recordings will allow disabled individuals to skillfully exert motor control of assistive robotic arms and generate verbal output using a computer communication interface.**

In a cohort of 8 subjects, we propose to record and monitor neural signals using subdural electrodes and a mounting and processing system which are already FDA cleared for the general indications of recording and monitoring of brain electrical activity. Specifically, the 510(k) documentation for the Blackrock NeuroPort system and NeuroPort Array states that it "is for temporary (< 30 days) recording and monitoring of brain electrical activity" (K060523 and K090957). The 510K documentation for the PMT Subdural Cortical Electrodes states that it is "cleared for temporary (<30 day) use with recording, monitoring, and stimulation equipment for the recording, monitoring and stimulation of electrical signals on the surface of the brain" (K082474). The Blackrock Microsystems 256-channel pedestal and 256-channel Blackrock Microsystems CereplexE256 digital headstage will also be used in this study (Appendix E). The Blackrock NeuroPort Biopotential Signal Processing System and NeuroPort Electrode Array have been used in human translational clinical studies to record neural activity over a number of months and years and enabled tetraplegic patients to volitionally control computer cursor position and an anthropomorphic prosthetic limb with 7 degrees-of-freedom (Ajiboye et al. 2012, Bouton et al. 2016, Collinger et al. 2013, Hochberg et al. 2012b, Hochberg et al. 2006, Kim et al. 2008b, Simeral et al. 2011). The PMT Subdural Cortical Electrodes have been used extensively in patients for epilepsy monitoring. The combination device of Blackrock Microsystems NeuroPort system, NeuroPort Array pedestal, and PMT Subdural Cortical Electrodes has been implanted in a non-human primate to successfully monitor overt reaching movements for 666 days without signal

degradation or without significant damage to the brain cortex. We propose to perform long-term monitoring and testing with the Blackrock/PMT combined system over similar time scales as the aforementioned studies, with the aim of motor and communication control.

We further propose that our investigational device will bear the statement, "CAUTION – Investigational device. Limited by United States law to investigational use."; all devices that will be used in this study will be labeled for investigational use and all labels and labeling will contain the investigational device statement .

Upon UCSF Institutional Review Board (IRB) approval of our investigational plan and protocol, we propose to conduct testing in two phases for each subject. We anticipate that study visits will be conducted in the outpatient office and/or home setting based on participant preference and other practicalities.

This is a single-center study of the use of a ECoG-based neural interface for testing the feasibility of using ECoG signals to control complex devices for motor and speech control in adults affected by neurological disorders with severely impaired motor and communication.

PMT Subdural Cortical Electrodes will be surgically placed directly on the brain surface over the motor and language cortices of subjects with severe disorders of motor control and communication. After implantation of the electrode, study patients will undergo training and assessment of their ability to control assistive robotic arms and determine if ECoG brain signals can be decoded for language communication. This will be performed in two phases.

Phase 1: Optimize BCI system
In Phase 1, we will optimize the entire system to reliably detect neural activity to ensure that the recorded signals are stable and free of artifacts. Moreover, we will ensure that the neural signals can be converted in real-time into cursor or external object movements. This phase will be conducted in the outpatient office setting and/or the patient's home environment, based on patient preference and needs. We anticipate that this phase will take ~1 month; we will, however, specifically tailor the period for each subject.

Phase 2: Testing of BCI Control
In Phase 2, we will test feasibility for both neuroprosthetic control and for decoding speech from neural activity. We will begin experimental testing with the system for control of assistive robotic arms that is classified as a non-significant risk device. As outlined in the sections below, we have multiple safety features to ensure that there is minimal risk for injury during use of the robotic arms, including subject contact during object interaction and the embodiment phase (i.e. the subject's arm is interacting with the exoskeleton).

Throughout this period, neural signals will be recorded and analyzed, and tasks will be performed toward the development, assessment, and improvement of the neural interface system. We anticipate that testing will be conducted in the outpatient office or home setting based on the patient's preference and needs. In this phase, we will also continue to perform experimental testing

with the communication interface; we will assess the ability to control a communication interface. We anticipate that this phase will take a minimum of 10-11 months.

4.0 Patient Eligibility

Study subjects will be adults with severe motor impairment secondary to a neurological disorder.

Inclusion Criteria

- Age 18-75 years old
- Limited ability to use upper limbs and limited ability to use speech, based on neurological examination by a board-certified neurological specialist, due to stroke, spastic quadriplegic cerebral palsy (with no significant cognitive deficits based on formal neuropsychological testing), amyotrophic lateral sclerosis (ALS), multiple sclerosis, cervical spinal cord injury, brainstem stroke, muscular dystrophy, myopathy or severe neuropathy
- Disability, defined by a 3 or greater score on the Modified Rankin Scale, must be severe enough to require some assistance, but able to walk without assistance of another person
- If stroke or spinal cord injury, at least 1 year has passed since onset of symptoms
- Must live within a two-hour drive of UCSF

Exclusion Criteria

- Pregnancy or breastfeeding
- Inability to understand and/or read English
- Inability to give consent
- Dementia, based on history, physical exam, and MMSE
- Active depression (BDI > 20) or other psychiatric illness (active general anxiety disorder, schizophrenia, bipolar disorder, obsessive-compulsive disorder (OCD), or personality disorders (e.g. multiple personality disorder, borderline personality disorder, etc.)
- A baseline Columbia Suicide Severity Rating Scale (C-SSRS) with positive suicidal ideation or behavior occurring within 5 years prior to enrollment
- Comorbidities including uncontrolled hypertension, cancer, or major organ system failure.
- Prior intracranial surgeries that could increase the risk to the study participant or impact the effectiveness of the investigational device
- History of substance abuse within the last year
- Ongoing anticoagulation which cannot be stopped in the peri-procedural period.
- Inability to comply with study follow-up visits
- Immunocompromised
- Has an active infection
- Has a CSF drainage system implanted or revised less than 1 year ago, history of prior CSF drainage system implant infection, presence of concurrent external ventricular drain, an active CSF leak, or other potential risk factors of CSF drainage system infection.
- Requires diathermy, electroconvulsive therapy (ECT), or transcranial magnetic stimulation (TMS) to treat a chronic condition
- Has any type of cranially-implanted generator, such as a cochlear implant or deep brain stimulator
- Allergies or known hypersensitivity to materials in the Blackrock NeuroPort Array pedestal or the Blackrock 256-channel pedestal (i.e. silicone, titanium) or the PMT Subdural Cortical Electrode (silicone, platinum iridium, nichrome)

5.0 Study Device(s)

Cleared for same indication with off-label use of device for at least 1 year:

- Blackrock NeuroPort Array, PN 6248
- Blackrock NeuroPort system, PN 5416
- PMT Subdural Cortical Electrodes, Model #2110TX-128-005, Model #2110TX-253-001

Modifications to Blackrock NeuroPort System with same indication with off-label use of device for at least 1 year:

- Digital Hub, 10480
- NeuroPlex E, 10908

Not cleared:

- Blackrock Microsystems 256-channel pedestal connector (see Appendix E for justification of use)
- Blackrock Microsystems 256-channel CereplexE256 digital headstage (see Appendix E for justification of use)

6.0 Study Procedure

Study Duration: The duration of this pilot study will be up to 12 years. We expect that all 8 patients will be recruited in the first 4-5 years, and data collection, device development and analysis will be completed in 12 years.

Patient recruitment and clinical characterization: Patients with motor impairments secondary to neurological disorders will be recruited from clinics specializing in the treatment of stroke, ALS, and general neurological disorders, at UCSF.

Enrollment procedures: Prior to enrollment into the study, an informal phone interview to schedule an office-based evaluation will take place, followed by three outpatient screening visits. During the first outpatient visit we will describe the trial in detail and answer all questions. Should the participant choose to continue, we will schedule another visit to conduct a physical exam and to perform screening labs to determine eligibility. During this visit we will screen for eligibility by
1) acquiring patient demographics, 2) reviewing medical history and measuring vital signs, 3) ensuring patients are not currently pregnant or plan to become pregnant, 4) obtaining a list of current medications being taken, 5) ensuring MMSE scores are within a reasonable range (≥18, also accounting for motor difficulties with taking the test), 6) obtain baseline patient-rated and investigator rated clinical global impression scales, 7) assess baseline health status with SF-36 Health Survey, 8) assess current depression and anxiety states with Beck Depression and Anxiety inventories (BDI and BAI, respectively), 9) determine suicidal ideation risk is minimal with Columbia Suicide Severity Rating Scale (C-SSRS) 10) perform a baseline neurological physical exam and 11) determine the disability rating using the modified Rankin Scale. An MRI and CT of the brain will also be obtained for future surgical planning and to further determine eligibility. Moreover, a ECG and chest x-ray will also be obtained. We will then schedule a third follow-up visit to review this data and to answer remaining questions.

Pre-operative care: Subjects will be administered Kefzol 2g IV within 60 minutes prior to surgical incision, and continued for 24 hours post-operatively. In case of allergy to Kefzol, Vancomycin 1gm IV will be used.

Surgery: After obtaining informed consent, subjects will undergo brief general anesthesia (typically ~ 3-4 hours) for the surgical procedure. This may be either IV or inhaled anesthetics. Patient will be given IV antibiotics prior to incision, and re-dosed if required. Induction and wake-up will take place in the operating room, and patients will be monitored in the post-anesthesia care unit (PACU) after surgery for 2-3 hours during the peri-operative period. Subject will then continue to recover on the surgical ward for 2 days.

Subjects will undergo surgical placement of an ECoG array over the dominant (usually left-sided) sensorimotor and speech cortices. The basic operative procedure is similar to what is commonly performed for non-penetrating subdural grid placement in patients with intractable epilepsy, which is well tolerated and has few complications [35]. This surgery will be smaller in exposure given that coverage will be limited to the regions of interest, and not broadly applied as used for epilepsy localization. For placement of the 253-channel electrocorticography array, the craniotomy will be the same size as for the 128-channel array. The craniotomy will also be smaller in exposure compared with the broader exposure for epilepsy localization, which usually includes placement of two 128-channel electrocorticography arrays. Standard procedures for craniotomy at the surgical site will be followed. Briefly, a 5-6 cm curvilinear incision will be made over the anatomic hand representation of the cortex (i.e."hand knob") which is located 3 cm lateral to the midline. Localization will be confirmed using intraoperative BrainLab stereotactic neuronavigation. A craniotomy will then be performed, exposing the dural surface. A wide slit in the dura will be opened.

After identifying the location for the electrode grid implant, the position for the pedestal connector is determined on the adjacent or contra-lateral skull surface and marked. A separate 2 cm scalp incision will be made for the pedestal connector. Holes will be drilled for connector placement and the connector will be secured to the skull with 8 small titanium screws. Once the connector is placed, the electrodes and wire bundle are gently manipulated to position the electrodes so that they are resting on the cortical surface over the area of interest. The nonpenetrating ECoG microarray will be sutured to the dura to secure its position. After successful placement of the electrode grid, the dura will be sutured closed in a watertight fashion and the bone flap will be replaced and secured in place with standard titanium cranial fixation plates and screws.

The surgical site will be irrigated with antibiotic lactated ringers solution. The fascia and skin will be closed with absorbable sutures over the covered craniotomy with a slit accommodating the passage of the connector. The wound will be dressed with a sealed surgical bandage. The expected blood loss for this procedure is 50 cc. The expected operative time is 3-6 hours.

The ECoG electrode array is manufactured by PMT Corporation, and is already FDA-cleared for temporary (<30 days) clinical monitoring of neural signals (K082474). The PMT Subdural Cortical Electrodes are a chronically implantable array containing 128 or 253 electrodes, with an electrode spacing of 4 mm or 3mm, capable of recording from areas of the central nervous system for extended periods of time. The electrode contacts are composed of medical grade platinum iridium, and embedded in a thin sheet of medical grade silastic. The model number 2110 indicates a platinum iridium wire and platinum iridium contacts. Platinum iridium is a mechanically robust alloy electrode material used in commercial DBS leads (Petrossians, Whalen and Weiland 2016). The electrodes' contacts are molded into a silicone rubber matrix in a fixed pattern. PMT Corporation electrodes are used routinely at our institution and others for seizure localization. In the past ten years, we have not encountered any adverse inflammatory reactions or bleeding specific to the electrode arrays itself (over 100 cases). Insulated wires extend from each electrode through a flexible silicone tube to the connector on the pedestal.

Post-Operative Care: Subjects will be recovered in the intensive care unit and observed for 24 hours before transferring to the ward. A postoperative head CT will be obtained to evaluate for any hemorrhage and to confirm the position of the array. IV antibiotics (Kefzol will used if there are no allergies) will be administered for 24 hours after surgery, followed by prophylactic antibiotics given up to suture removal plus 2 days. Patients will be switched to oral antibiotics as soon as possible. Subjects will undergo physical exam every day with monitoring of vital signs, neurological exam, and basic respiratory, cardiac, and gastrointestinal exams. In addition, the wound site will be inspected at all study visits.

Routine hygiene will consist of handwashing with soap/water and donning gloves using sterile technique when coming in contact with the percutaneous connector. The caregiver and patient will be trained to clean the pedestal site according to specific instructions that will be given to them, to be performed at least once every seven days or as required.

An infection control protocol will be strictly followed. At early signs of infection or irritation the patient/caregiver should contact the study physician immediately. The surgical site will need to be cleaned twice or more during the day, while using extra meticulous hand hygiene. If skin erythema, edema, pain and/or warmth are present, the available drainage will be cultured and oral antibiotics will be started. Plain radiographs of the involved area, ESR, CRP, WBC, and blood cultures will be obtained. If there is no improvement within 72 hours, rapid progression of erythema, symptoms worsen, or if there are signs of systemic toxicity, parenteral antibiotics is warranted. If high fever or severe pain is present, nuchal rigidity, or progressive deterioration in level of consciousness, the participant should go to the hospital emergency room. All implanted hardware will be surgically removed if there is evidence of hardware infection.

Pain related to small craniotomies is usually self-limited. Pain scale ratings will be assessed every 4 hours and routine postoperative pain management will be used. This includes the following medications as needed: acetaminophen and/or Percocet (acetaminophen/hydrocodone). IV pain meds (morphine sulfate or dilaudid) will also be administered if needed. In our experience, most patients do not require IV analgesics beyond the first operative day for smaller craniotomies.

Study visits: As outlined above, system testing will occur through two phases. In the first phase, we will simply optimize the system; in the second phase, we will commence testing of the motor and communication neural interface systems.

In **Phase 1**, the main goal is to ensure reliable neural signal monitoring and optimization of the real-time systems. Initial study visits will occur at defined time points, similar to those normally used in clinical care. For example, we anticipate a visit at post-op day (POD) 10 and 14. During this time, we will simply monitor the neural signals and briefly test the real-time communication with the computer interface. We anticipate additional visits 1-3x/week, based on the patient's availability and preference, to continue to monitor signal stability and check for wound healing. During this phase, we will also offer in home testing of the system to minimize the burden. While the equipment may be kept at the subject's home, testing will only occur when study personnel are

present. The total time period of Phase 1 will be approximately 1 months, the exact time will be customized for each patient.

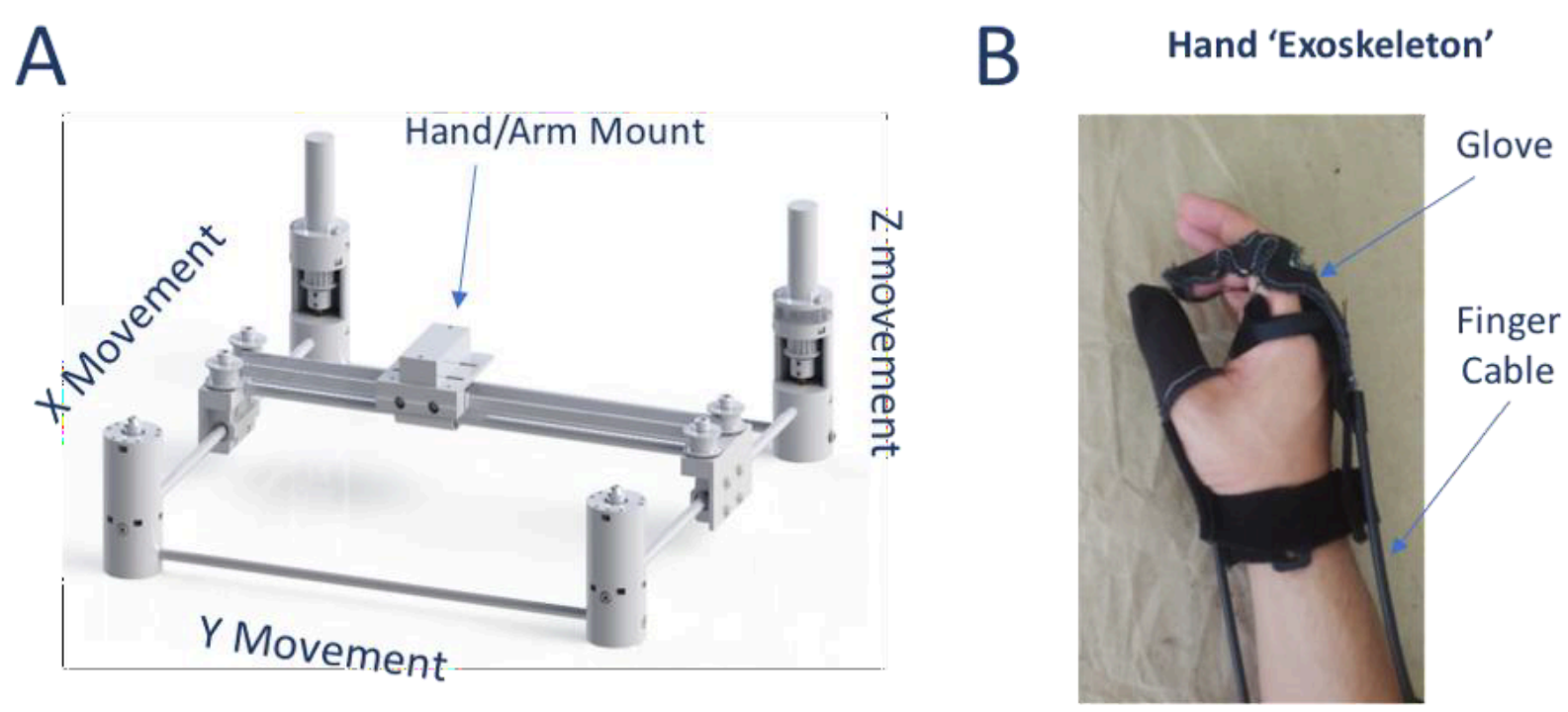

In **Phase 2**, we will test feasibility for both neuroprosthetic control and for decoding speech from neural activity. We will begin to perform experimental testing with the system for control of assistive robotic arms including a custom wearable hand exoskeleton robot and a surface mounted robotic arm which can be classified as non-significant risk devices. As shown in panel A, the exoskeleton system consists of a table top frame that allows x,y,z movement of the arm/hand (i.e. supported by a brace attached to the platform mount). Movements are limited to the natural workspace of each subject. The hand exoskeleton (panel B) will be mounted using a readily releasable magnetic mount. The hand system aims to allow control of the fingers and thumb using a motorized cable system attached to motors. This system will allow us to test restoration of reach to grasp functions in our subjects. The surface mounted robotic arm is a commercially available investigational device for installation on wheelchairs of users with functional limitations or upper body disabilities. It is designed to support active tasks such as eating, drinking, and personal hygiene. There are three options available for control of the robotic arm, including joystick control, a graphical user interface, and an option offering developers to customize control of the robotic arm through a software interface. This system will allow us to test control of a surface mounted robotic assistive arm. There will be no direct physical contact between the Kinova robot arm and gripper and the participant. As outlined in the Risk Analysis (Appendix B), we have multiple safety features to ensure that there is minimal risk for injury during the embodiment and use phase (i.e. the subjects interact with the exoskeleton and surface mounted robotic arm).

Throughout this period, neural signals will be recorded and analyzed, and tasks will be performed toward the development, assessment, and improvement of the neural interface system. We will assess quality of performance using kinematic parameters while performing required tasks. We will analyze stability of neural recordings and performance. To measure stability of the neural representation we will analyze the neural correlates of imagined movements. We will also examine the stability of neural correlates on neuroprosthetic control (e.g. spectral content, timing, spatial recruitment). We will assess changes in the spatial correlation scales and other redundancy measures during learning and stable performance. We anticipate that testing will be conducted in the outpatient office or home setting based on the patient's preference and needs.

In this phase, we will also continue to perform experimental testing with the system for control of a virtual communicating interface. Throughout this period, neural signals will be recorded and analyzed, and tasks will be performed toward the development, assessment, and improvement of the neural interface system. We will assess the ability to control a computer communication interface. To measure stability of the neural representation we will analyze the neural correlates of imagined speech. We will also examine the stability of neural correlates on a neuroprosthetic communication device (e.g. spectral content, timing, spatial recruitment).

Neural activity monitoring: Continuous neural signal data will be acquired from the 253 or 128-channel implanted PMT Subdural Cortical Electrodes and processed with the Digital NeuroPort system hardware by Blackrock Microsystems. Broadly, the neural data will consist of ECoG neural activity from neurons in the vicinity of each recording electrode. The neural data will be actively monitored and recorded via the graphical user interface associated with the NeuroPort hardware commercially available via Blackrock. Data read and download are non-invasive and will be performed with the patient comfortably rested. For both the 128 and 253-channel system, the patient will have one or two high-definition multimedia interface (HDMI) connections from the digital headstage to the digital hub for data acquisition. To mitigate the risk of any pulling, snagging, or tension to this connection, we will position our patients such that there is no activity between the digital headstage and the digital hub. Additionally, to further mitigate any risk of head movement which could lead to pulling, we will offer our patients neck support during each session.

The NeuroPort system has been successfully deployed in monitoring neural activity in patients with motor control disorders (e.g. (Hochberg et al. 2012a, Pandarinath et al. 2015)). Blackrock Microsystems provides commercial software that allows real-time filtering, recording, and visualization of acquired neural data and also allows interfacing with other programming languages such as MATLAB (Mathworks, MA) or Python (Python Software Foundation, VA). Together, this allows the ability to create custom software such as communication device based on neural spiking activity, detailed further in the following section. Overall, the mix of commercial and custom software will allow for real-time signal processing, synchronization, and control of the peripheral communication device, with parallel data streams to store data for offline analyses.

Online BMI control of Assistive Robotic Arm Devices:
ECoG signals will be filtered and processed in real-time using a customized portable multi-channel neurophysiology workstation NeuroPort Biopotential Signal Processing System. We will bandpass each channel into multiple bands. Past experiments, including our own, suggest that movement related information is encoded in these bands.

Initialization Phase.

We will train a ‘decoder’ that maps neural activity to the movement of an assistive robotic arm device. Recent experiments suggest that such methods can rapidly allow control of neuroprosthetic devices. We will train the neural decoder using ‘imagined movements’. As paretic/paralyzed patients will not have access to normal overt movement related neural signals, we will use the

neural basis of imagined movements for training. During the training phase, patients will observe a computer screen with either a cursor or a simulated robotic arm. Initial online decoder performance can be assessed in this virtual environment. We will then aim to transition to physical control of the assistive robotic arm devices (with all the safety constraints outlined in this submission).

In order to compare visual versus visual + proprioceptive/tactile feedback signals during decoder conditioning, we will use the exoskeleton setup for decoder conditioning. The arm and hand will move in a stereotyped fashion while the subjects are instructed to 'imagine' actively tracking its path. Of note, we have experience with similar control of an exoskeleton system (Ganguly et al. 2011, Ganguly et al. 2009). As prior and as outlined below, the current system is developed by the Robert Matthews, PhD and Ruzena Bajcsy, PhD (UC Berkeley). They have long-standing experience with kinematic monitoring, limb dynamics and exoskeleton development (Matthew et al. 2015, Matthew et al. 2016, Oskarsson et al. 2016).

The training of the Kinova robot arm will occur with the arm mounted to either 1) a table adjacent to the subject or 2) the participant's wheelchair. The robot arm will not be powered by the wheelchair's power source, and will only be powered by a wall electrical outlet as intended. In either configuration, the robot will not make any physical contact with the participant. The robot will initially be mounted to a table and positioned greater than 48 in from the subject, ensuring that the subject is outside of the physical reach of the robot. Following at minimum two weeks of testing and demonstrated proficiency of control, the robotic arm will be moved to be closer to the participant. At this point, the robot may be directly mounted to the participant's wheelchair or on a table adjacent to the subject. The integration of the Kinova robotic arm and the participant's wheelchair will be completed following Kinova's instructions outlined in the following integration guides: IG-003 and IG-004, and in consultation with Kinova's Assistive Product Manager and their Field Application Engineers. This location of the robotic arm allows for the performance of tasks in a configuration consistent with how wheelchair-mounted assistive robotic arms are used in real-world settings. The tasks performed will not involve physical interaction with the participant. The subject will be able to control the endpoint of the Kinova robot along with orientation of its gripper and the grasper. Participants will practice object interactions such as controlling the robotic arm to pick up a ball and moving it to a second location. Testing of the robotic arm will only be performed with research study personnel present. Participants and caregivers will not use the robotic arm outside of testing with study personnel. In addition, the Kinova robotic arm will only be powered by a wall electrical outlet, and not by battery power.

While testing is conducted with the wheelchair or table mounted robotic arm, experimenters will visualize all channels of data in real-time and monitor for large variations in the recorded neural signals to detect the presence of noise or artifacts that may suggest electromagnetic interference. Additionally, we have implemented detections to determine if the recorded signals significantly differ from historical values or the baseline values recorded at the start of the session. If significant variations are detected, we will power off the robotic arm to determine if it is the source of the noise.

Training Phase. Subjects will be allowed to practice tasks associated with arms/hand and object manipulation. The position of the robotic arm end effector coordinates (x, y, z) will be under direct neural control. Preliminary experiments with an exoskeleton system showed that end effector control (position and orientation of the wrist) was more intuitive and efficient than position control of individual joints. For this initial phase, the motion will be restricted to a 2-dimensional plane for reaching and grasping objects. There are currently 9 tasks which involve interactions with both static and dynamic environments with various fixtures. We will first limit movements to a 2D environment. The additional degree of freedom involving grasp will be included based on proficiency. In addition, given that 'motivation' and reward are known to influence the overall learning process, a gamed-based training environment with specific goals and scoring systems were developed to engage the subject intellectually and to provide additional enrichment during the training phase.

Testing Phase. To assess robustness and stability of control over days we will assess performance characteristics in three tasks. A) Standard center-out task where subjects have to move to the center, engage a grasp, then move to a target and disengage the grasp to release an object such as a ball. The workspace will be at 95% of the patient's natural reach. Target size will be kept at 5 cm. B) Reaching from a randomized starting and end position in the workspace of Task A. C) Task B except with the need to plan around obstacles that are placed in the direct path.

Online BMI control: Also during phase 2, neural activity will be used to control a real-time communication device using state of the art closed-loop decoders based on rapid changes in neural activity (Shanechi, Orsborn and Carmena 2016). The main advantage of such a decoder lies in its enhanced ability in discriminating user intent and its speed of operating at every event. Such decoders operate at much higher speeds (typically at 200Hz) over previously developed decoders that rely on averaging neural activity (typically at 10Hz). In addition, we will compare this decoder to more standard decoders (e.g. the Weiner Filter, the LMS filter, the Kalman filter and variants). As documented below, the main outcome measure will be the rate of communication using these approaches.

A virtual communication effector will be presented on a computer screen, custom written in the MATLAB or Python programming environment. The interface between MATLAB or Python and the Digital NeuroPort system will be via custom software or commercial software provided by Blackrock Microsystems. The novel decoder will map neural activity to the communication interface. A language processing engine will be concurrently running in the background to model and predict the words and sentences. The following metrics will be utilized to measure performance of the decoder and communication device: selections per minute, accuracy, correct characters per minute (Bacher et al. 2015b) and the bitrate, an information theoretic approach to relate accuracy, time of task completion and complexity (Nuyujukian et al. 2014b, Thompson et al. 2014a). The performance of the novel decoder based communication device will be compared to traditional state space filtering decoders such as the Kalman Filter that has been previously successfully deployed in similar BMI paradigms (Gilja et al. 2012, Bacher et al. 2015b).

Development life cycle of the BMI motor control and communication software:

a) Scope: The intended use of the decoder is to allow the patient to achieve control of external devices and thereby select characters and letters to form sentences, as well as control assistive robotic arm devices. As such the operation of the BCI is therefore dependent on the functionality of the software.

b) Platform: The software will be developed on MATLAB (The Mathworks, MA) and MATLAB supported C/C++ complied programs (MEX files) or (Python Software Foundation, VA) and will be running on the data acquisition PC that interfaces with the Blackrock NeuroPort Array pedestal connector or on a separate real-time processing machine (as in [Moses*, Metzger*, Liu* et al., 2021]). We will use the software libraries that are part of the Blackrock Digital NeuroPort system, potentially in coordination with custom software, to stream neural data into MATLAB or Python in real-time. All neural data will be streamed through the Blackrock Microsystems Neural Signal Processor before streamed by HDMI cord to MATLAB or Python in real-time.

c) Inputs and outputs: The inputs to the software will be the neural signals from the PMT Subdural Cortical Electrodes grid and the output of the software will consist of user controlled (via the user's neural signals) effector position, selections of characters, letters and numbers for communication purposes, in addition to control of a movements of assistive robotic arms.

d) Components: There are four distinct aspects of the software, three that operate 'behind the scenes' and one that serves as a Graphical User Interface for display. First, is the decoder itself that translates neural signals into user intentions. Second, is another parallel decoder that serves to discriminate when the patient has made a particular selection (or e.g. grasping actions). Third, is the software engine that keeps track of the current selections made and generates a list of probable options using a statistical model of movement direction and language (Nadkarni, Ohno-Machado and Chapman 2011b). The fourth and final aspect of the decoder is the Graphical User Interface (GUI) that displays and controls the real-time position of the end effector.

e) Safety: The software provides only visual feedback to the user and does not directly interface with the neural signal data acquisition process. The software only serves to allow the use to control the communication interface and the robotic arm position.

f) Planning phase: In the planning phase, we will identify off-the-shelf components (such as language processing engines) and will aim to further refine and customize it in-house concurrently with the decoders and GUI.

g) Development phase: In the development phase, all components will be developed in parallel as discrete subunits of the overall functional system. A code repository will be maintained to keep track of the life cycle versions and code will be commented wherever appropriate. During the development phase, debugging will be performed at every iteration and documented. The documentation and code will be maintained on secure hard drives.

h) Testing phase: There are two aspects of this phase. One is the performance testing of each of the four individual components and the other is the testing of the entire software. In lieu of actual neural signals, simulated neural signals will be delivered as input, with a known mapping between the input and output state as the ground truth is known a priori. This will allow testing the performance of the decoders (accuracy in estimated positions). The testing of the GUI and the software engine will be performed independently of the decoders by manually controlling the position of the effector. The testing of the GUI and the software engine will be assessed by the stability and

reliability in updating effector position and robotic arm movements, in the turnaround time of displaying the list of predictive words and actions based on current selections. The overall system testing will employ a combination of simulated neural signals and manual position control to assess the ability of the software in allowing a user to communicate and control a hand neuroprosthetic.

i) Error handling: Code will be written to specifically monitor potential sources of errors in real-time decoding due to either noise in neural signals or decoder weight drift that would necessitate recalibration and resetting of the GUI and software engine.

j) Software validation: The validation and formal design review for the overall software will be performed by members of the PI's laboratory not involved with the development and testing of the software prior to software deployment.

k) Resolution and maintenance: Active documentation and daily logs will be noted to keep track of the performance of the software and address issues such as version control, robustness and immediate resolution of unforeseen errors in the software.

Progression of phases: We anticipate phase 1 will last approximately 1 month but will vary based on each subject. Phase 2 will last at a minimum 10-11 months, a total amount of time of at least 1 year after PMT Subdural Cortical Electrodes implantation and neural interface monitoring and testing, a timeframe which has been performed or exceeded without adverse effects by previous studies using the PMT Subdural Cortical Electrodes and Blackrock Microsystems NeuroPort Array pedestal, and NeuroPort system. In this study, recordings were made over 666 days in a non-human primate with no adverse events related to the implanted devices (Degenhart et al. 2016). The ECoG array is identical in manufacture and material to that used for subdural grid placement in patients with intractable epilepsy, which is well tolerated and has few complications (Chang et al. 2010).

Activation of the brain recording function: All data collection in the study visits will be initiated by the study staff.

Conclusion of study: For each enrolled subject, if there have been no serious adverse events, we will present the option to continue with the study at the end of a 1-year period. If the subject chooses, we will continue with testing for another year. We will formally present this option every year for a period of 5 years. Notably, the subject will be reminded that he/she will have the option for surgical removal of the device at any point.

Removal of the ECoG grid and Connector pedestal: At the conclusion of the study, or earlier if medically indicated, the subdural cortical electrodes and connector pedestal will be surgically removed. The skin incision and bone flap will be reopened and the electrode will be removed and discarded. The dura will be sutured tightly. The galea and scalp will be sutured closed. The expected blood loss is minimal (less than 10 cc), and the expected operative time is 30 minutes.

Replacement of the ECoG grid and Connector pedestal for participant B1: Nearing the end of the 5 year period after device implantation, based on multiple other factors such as funding, staffing, clinical and research considerations, etc., we may offer participant B1 the option to replace the original electrode array and pedestal port with the 256-channel Blackrock Microsystems connector pedestal and 253 channel PMT Subdural Cortical Electrode, a potentially higher performing FDA IDE approved investigational device. If device replacement occurs, participant

B1 will again be presented the option for device removal or to continue in the study every year for a period of up to an additional 5 years. If not, explantation will be the default at the end of 5 years. Device replacement will allow this participant who is already well trained in BCI control, has expressed a strong interest and has proven motivation to continue to contribute to and further advance research efforts for an additional 5 years, by taking advantage of improved device technology. A new consent form, separate from the original consent form, will need to be signed by the participant. Explantation at the end of the 5 year period will remain the default if this option is not offered or is declined by the participant.

This participant will incur additional risk related to undergoing an additional surgical procedure to replace the original device. These risks, however, are mainly similar to the general risks previously described in the Risk Analysis under "Standard Brain Surgery for Implantation of a Device", and similar risk mitigation measures will be applied. In addition, the cortical surface and surrounding tissues will be carefully examined after the removal of the original device. If there are any signs of infection, other concerning clinical findings, or if any technical difficulties are encountered, then we will only perform device explantation and will not proceed with device replacement. Re-implantation may also be associated with higher risk due to the presence of scarring resulting from the previous surgery. This risk will be addressed with the surgical plan below. Overall, the risk of device replacement at the time of device explantation reduces the overall risks compared to the alternative of device explantation separate from future implantation of a new device.

In addition, the potential benefits for re-implanting our first participant B1 with the updated investigational device rather than extend the duration of testing with his current device are manifold. First, there is compelling existing evidence from outside of this clinical trial that increased ECoG density beyond the 4-mm center-to-center spacing of the original investigational device would enable higher-fidelity signal acquisition and ultimately increased decoder performance (Duraivel et al. 2022; Muller et al. 2016). This was then further corroborated by our work with our more recent participant, B3, where we show that the updated 253 channel grid investigational device (with a 3-mm center-to-center spacing) enabled speech decoding that was significantly superior to what we have shown with our first participant with the original investigational device (Metzger et al. 2023). By using the 253 channel grid investigational device with our first participant, we will gain further insight into the efficacy of the updated investigational device for enabling robust speech decoding from brain activity.
Second, re-implanting the first participant with the 253 channel grid will enable an unprecedented opportunity to compare and contrast acquired signals and decoder performance between the original and updated investigational devices without the confound of variability between participants. Each brain is slightly different, and each participant will be different from other participants in ways that may be difficult to fully account for. In this case, we will have much closer to an "apples-to-apples" comparison in which we can perform more principled assessments between our observations with the two types of devices. This will help us understand how much of the differences in performance that we've observed between our first participant and the most recent participant are attributable to the updated device (versus other factors, such as individual variability), providing valuable insight that could inform the design of future brain-computer interfaces.

The previously implanted device will be surgically removed by re-opening the original incisions, temporary removal of the bone, and removal of the PMT Subdural Cortical Electrodes from the

brain and pedestal from the skull. The cortical surface and surrounding tissues will be examined. If there are any signs of infection, or if any technical difficulties are encountered, then we will proceed with device explantation only. Otherwise, participants will undergo surgical placement of the new ECoG array over the same sensorimotor and speech cortices.

Re-implantation may be technically more difficult due to the presence of potential adherent scarring resulting from the previous surgery. Considering the scar tissue can be associated with a potentially more complicated surgery, there is an inherently greater risk of complication and damage to the tissues with resultant irreversible neurological injuries that can impact the individual's function. To reduce bleeding and to avoid cautery use, we will use pre-incision injection of lidocaine with epinephrine to reduce scalp bleeding hemostasis. We will also use Raney clips to reduce bleeding from the scalp edge. The next step will require removal of the bone flap by removing the titanium plating and careful dissection of the bone flap from the dura. The dura over the array will be exposed and it will be gently removed from the cortical surface. We expect the array to slide off smoothly, but it is possible there may be some encapsulation or fibrosis and this may require careful dissection under loupe visualization. We will use hemostatic agents, such as Floseal hemostatic matrix (gelatin matrix and thrombin), as needed for additional hemostasis. Once the array is dissected out, we will cut the leads to the Blackrock percutaneous pedestal port and remove the array. The screws anchoring the port will be removed, and the pedestal port will be removed off the field. The new replacement 253-channel grid will be placed over the location of the prior grid, and the pedestal port shall be fixed to the same percutaneous site. A separate 2 cm scalp incision will be made for the new pedestal connector if needed. The pedestal will then be secured to the skull with 8 small titanium screws. Meticulous care will be taken to minimize blood loss and injury to cortex. If there is too much scarring or bleeding, the replacement array will not be implanted.

The dura will be re-sewn together, and the bone fixed again with titanium screws.

The expected operative time is 3-6 hours. Induction and wake-up will take place in the operating room, and patients will be monitored in the post-anesthesia care unit (PACU) after surgery for 2-3 hours during the peri-operative period. After surgery, the patients will be admitted to a neurosurgical transitional care unit and monitored for any postoperative complications. The participant will then recover in the intensive care unit and be observed for 24-hours before transferring to the surgical ward for 2 days. A postoperative head CT will be obtained to evaluate for any hemorrhage and to confirm the position of the device. IV antibiotics will be administered for 24-hours after surgery followed by prophylactic antibiotics given up to suture removal plus 2-days.

Subjects will remain in the hospital for monitoring for approximately 3-5 days until deemed medically stable for safe discharge. Caregivers will again be trained to perform skin care around the pedestal site with proper sterile handling and cleaning techniques, as well as procedures for identifying and reporting clinical signs of infection. Caregivers will be instructed to contact the Study PI immediately for any potential Adverse Events."

7.0 Clinical Measurements

Primary

This is a pilot study to test feasibility in eight subjects.

For BCI motor control, we will use outcome measures that are frequently used in preclinical studies of neuroprostheses (e.g. accuracy and reliability of cursor and limb control). As outlined below, the primary goal will be to gather statistics regarding the best achievable control using ECoG signals and state-of-the-art methods to allow motor neuroprosthetic control. For each of the parameters below we aim to describe the statistics as the mean and the variance (Bacher et al. 2015a, Nadkarni et al. 2011a, Nuyujukian et al. 2014a, Shanechi et al. 2016, Thompson et al. 2014b). Ultimately, we will compare these values to a wealth of published data regarding movements in able-bodied subjects, e.g.(Bacher et al. 2015a).

1. *Quality of performance* will be assessed using kinematic parameters while performing the required tasks. We will assess stability of the trajectories in the tasks. We will then assess the ability for generalization from any region in the workspace to another random point. Position errors from the selected trajectory of the task as well as velocity and acceleration will be studied in both joint space and the end effector space.

2. *Recording and Performance Stability.* We will use previously established metrics to analyze stability of neural recordings (Shanechi et al. 2016, Thompson et al. 2014b) and performance. To measure stability of the neural representation we will analyze the neural correlates of daily imagined movements. We will also examine the stability of the neural correlates of neuroprosthetic movements (e.g. spectral content, timing, spatial recruitment).

3. *Spatial Scale.* An important question for ECoG recordings is the optimal spatial scale for the electrode grid. This has implications for maximizing the amount of information that can be obtained from the recording setup but also for defining the design specifications of implantable electronics (e.g. power requirements could vary greatly depending on the spatial and temporal resolution of the neural data required). We will look at changes in the spatial correlation scales and other redundancy measures during learning and stable performance.

The following metrics will further be utilized to measure performance of the decoder and communication device: selections per minute, accuracy, correct characters per minute (Bacher et al. 2015b) and the bitrate, an information theoretic approach to relate accuracy, time of task completion and complexity (Nuyujukian et al. 2014b, Thompson et al. 2014a). Physiological measurements related to neural activity will include statistical assessments of z-scored activity from single electrodes as well as population dynamics. Physiological measurements related to oscillatory activity will include: wide spectrum power-spectral analysis as well as using specific frequency

domain for mean log power (i.e. in the delta, theta, alpha, beta, gamma bands), coupling between the phase of low frequency rhythms and broadband gamma amplitude (phase-amplitude coupling, abbreviated PAC) (Canolty et al. 2006, Miller et al. 2010, Tort et al.).

Clinical measures and physiological measurements will be collected and recorded by members of the research and clinical team.

8.0 Data Management

All clinical and physiological data will be stored in encrypted and password-protected computers in the PI's laboratory that is always locked. If the Digital NeuroPort system is stored in the participant's residence, all research data will be maintained in accordance with UCSF standard encryption policy. In publications or presentations of the data, data will be grouped by case number in chronological order with no name identification. All patients will be asked to sign a separate consent for audio-video recording. When presenting videotape data at scientific conferences, we will utilize only videos from patients who have consented to have their videos shown. De-identified electrophysiological data may be shared with other researchers at other institutions.

9.0 Statistical Methods and Data Analysis

To assess the performance of the decoder, analyses will be performed on the kinematics associated with control, such as time to reach a target, trajectory curvature etc., in conjunction with measures associated with the communication device such as the bit rate, accuracy, characters per minute. The statistical reliability of the decoder will be assessed by non-parametric data permutation wherein the learned mapping between neural activity and the effector position will be artificially broken down and shuffled. Field potential data will be analyzed using wide spectrum power-spectral analysis as well as using specific frequency domain for mean log power (i.e. in the delta, theta, alpha, beta, gamma bands). We will also examine coherence and cross-frequency coupling between the channels (Canolty et al. 2006, Miller et al. 2010, Tort et al.). Using a repeated measures ANOVA statistical analysis, summary statistics for power in relevant frequency bands, control related power changes, and indices of phase-amplitude coupling will be compared at different time-points of control. Additionally, bootstrap statistical tests and general linear mixed models can be utilized to investigate potential statistical effects, given the small sample size. Mean, median, variance and median absolute deviation describing the statistics of each of the measured outcomes (such as accuracy, effector position control) will be recorded for each subject.

Sample size calculation: This is a pilot study to assess the feasibility of an ECoG based implantable BCI device in patients with motor control disorders using intracranial recordings, a communication interface and assistive robotic arms. The collected pilot data will aid in determining the feasibility, reliability and future directions of the brain machine interface for communication and motor control. In addition, the pilot data will be used to formulate more detailed hypothesis on neural plasticity and BMI control in humans. As such, there is no formal sample size requirement for this pilot study.

Criteria for study success that would justify a larger subsequent trial:

1. Ability to use ECoG-based neural activity to control a neuroprosthetic device and communication interface.
2. No permanent serious adverse events occur (such as trauma with long-term motor deficit).
3. Benefits to the patient in regaining a sense of control over the ability to exert motor control and communicate in an efficient manner.

10.0 Regulatory Requirements

Prior to the start of the study, the following documents will be collected and filed:

- Signed protocol signature page
- Curriculum vitae of the PIs and Sub-investigators, updated within 2 years
- Current medical licenses for the PIs and all Sub-investigators
- Financial disclosure form signed by the PIs and all Sub-investigators
- Copy of the IRB approval letter for the study and the IRB Membership List
- Investigator Agreement

Investigator Obligations

Redacted will be responsible for ensuring that all study site personnel adhere to all FDA regulations and guidelines regarding clinical trials, including guidelines for GCP (including the archiving of essential documents), both during and after study completion. Additionally, they are responsible for the subject's compliance to the study protocol.

All information obtained during the conduct of the study with respect to the patients' state of health will be regarded as confidential. This is detailed in the written information provided to the patient. An agreement for disclosure of any such information will be obtained in writing and will be signed by the patient.

Informed Consent

The investigators will obtain and document informed consent for each patient screened for this study. All patients will be informed in writing of the nature of the protocol and investigational therapy, its possible hazards, and their right to withdraw at any time, and will sign a form indicating their consent to participate prior to the initiation of study procedures.

Institutional Review Board

This protocol and relevant supporting data are to be submitted to the appropriate IRB for review and approval before the study can be initiated (UCSF, Human Research Protection Program, 3333 California Street, Suite 315, San Francisco, CA, 94118, FWA#00000068; IRB Registration 00000229, Lisa Denney, HRRP Director). Amendments to the protocol will also be submitted to the IRB prior to implementation of the change. The PIs are responsible for informing the IRB of the progress of the study and for obtaining annual IRB renewal. The IRB must be informed at the time of completion of the study and should be provided with a summary of the results of the study by the PIs. The PIs must notify the IRB in writing of any SAE or any unexpected AE according to ICH guidelines.

Data safety monitory board (DSMB) and safety monitoring plan

Treatment emergent adverse events that are assessed by the principal investigators as possibly, probably, or definitely related to surgical implantation or chronic cortical recording AND are unexpected or meet seriousness criteria (death, immediately life threatening, hospitalization >24 hours, persistent or significant disability, or significant intervention required to prevent one of the previously-stated outcomes) will be recorded and reported to the IRB, device manufacturer and the FDA via the MedWatch online voluntary reporting form within 10 working days of the study team's knowledge of the event.

All such events will also be reported to the data safety monitor board (DSMB), led by *Redacted*, who does not have direct involvement in this study but who has expertise in implantable devices, pain management and neurosurgery. The DSMB will meet regularly to review data related to the clinical trial, provide guidance and feedback, and review any adverse event reports. Treatment-related adverse events assessed as definitely, probably, or possibly related to study procedures and either serious or unexpected, noted by any study personnel will be reported within 10 working days of their knowledge of the event to the DSMB. The DSMB will then advise the PI on potential changes in procedures to improve safety. The safety endpoint will consist of all adverse events.

A Data and Safety Monitoring Board (DSMB), which includes an independent Medical Monitor and Neuroethics expert, will regularly assess reports of unanticipated study-related events. As a part of the DSMB, the independent medical monitor will receive mandatory adverse event reporting in the same timeframe as the DSMB. Apart from this, a report of accumulated adverse event data will be provided on a quarterly basis to the independent medical monitor for review.

Surgical and/or non-Surgical protocol defined adverse events will be recorded on adverse event case report forms. Any serious or unanticipated adverse events will be promptly reported to the DSMB, NIDCD, IRB and FDA in line with regulatory policies.

Throughout the clinical trial, should a serious adverse event occur that is assessed to be related to the presence or surgical implantation of the electrode system, such as infection, the device will be removed and the study halted for the patient. Removal will be accomplished by re-opening the original incisions, temporary removal of the bone, and removal of the PMT Subdural Cortical Electrodes from the brain and NeuroPort Array pedestal from the skull. The dura will be re-sewn together and the bone fixed again with titanium screws

Furthermore, if there is a serious surgical or non-surgical adverse event, or with the onset of suicidality, the study will be halted for the patient.

If two patients meet one or more of these criteria (i. serious surgical or nonsurgery-related adverse event, or ii. onset of suicidality), the study will be halted until information is reviewed by the DSMB and FDA.

References

Ajiboye, A. B., J. D. Simeral, J. P. Donoghue, L. R. Hochberg & R. F. Kirsch (2012) Prediction of imagined single-joint movements in a person with high-level tetraplegia. *IEEE Trans Biomed Eng,* 59**,** 2755-65.

Anderson, K. D. (2004) Targeting recovery: priorities of the spinal cord-injured population. *J Neurotrauma,* 21**,** 1371-83.

Bacher, D., B. Jarosiewicz, N. Y. Masse, S. D. Stavisky, J. D. Simeral, K. Newell, E. M. Oakley, S. S. Cash, G. Friehs & L. R. Hochberg (2015a) Neural Point-and-Click Communication by a Person With Incomplete Locked-In Syndrome. *Neurorehabil Neural Repair,* 29**,** 462-71.

Bacher, D., B. Jarosiewicz, N. Y. Masse, S. D. Stavisky, J. D. Simeral, K. Newell, E. M. Oakley, S. S. Cash, G. Friehs & L. R. Hochberg (2015b) Neural point-and-click communication by a person with incomplete locked-in syndrome. *Neurorehabilitation and neural repair,* 29**,** 462-471.

Bensmaia, S. J. & L. E. Miller (2014) Restoring sensorimotor function through intracortical interfaces: progress and looming challenges. *Nat Rev Neurosci,* 15**,** 313-25.

Birbaumer, N., N. Ghanayim, T. Hinterberger, I. Iversen, B. Kotchoubey, A. Kubler, J. Perelmouter, E. Taub & H. Flor (1999) A spelling device for the paralysed. *Nature,* 398**,** 297-8.

Bouchard, K. E., N. Mesgarani, K. Johnson & E. F. Chang (2013) Functional organization of human sensorimotor cortex for speech articulation. *Nature,* 495**,** 327-32.

Bouton, C. E., A. Shaikhouni, N. V. Annetta, M. A. Bockbrader, D. A. Friedenberg, D. M. Nielson, G. Sharma, P. B. Sederberg, B. C. Glenn, W. J. Mysiw, A. G. Morgan, M. Deogaonkar & A. R. Rezai (2016) Restoring cortical control of functional movement in a human with quadriplegia. *Nature,* 533**,** 247-50.

Canolty, R. T., E. Edwards, S. S. Dalal, M. Soltani, S. S. Nagarajan, H. E. Kirsch, M. S. Berger, N. M. Barbaro & R. T. Knight (2006) High gamma power is phase-locked to theta oscillations in human neocortex. *Science,* 313**,** 1626-8.

Carmena, J. M., M. A. Lebedev, R. E. Crist, J. E. O'Doherty, D. M. Santucci, D. F. Dimitrov, P. G. Patil, C. S. Henriquez & M. A. Nicolelis (2003) Learning to control a brain-machine interface for reaching and grasping by primates. *PLoS Biol,* 1**,** E42.

Chang, E. F., J. W. Rieger, K. Johnson, M. S. Berger, N. M. Barbaro & R. T. Knight (2010) Categorical speech representation in human superior temporal gyrus. *Nat Neurosci,* 13**,** 1428-32.

Chao, Z. C., Y. Nagasaka & N. Fujii (2010) Long-term asynchronous decoding of arm motion using electrocorticographic signals in monkeys. *Front Neuroengineering,* 3**,** 3.

Chestek, C. A., V. Gilja, P. Nuyujukian, R. J. Kier, F. Solzbacher, S. I. Ryu, R. R. Harrison & K. V. Shenoy (2009) HermesC: Low-Power Wireless Neural Recording System for Freely Moving Primates. *Ieee Transactions on Neural Systems and Rehabilitation Engineering,* 17**,** 330-338.

Churchland, M. M., J. P. Cunningham, M. T. Kaufman, J. D. Foster, P. Nuyujukian, S. I. Ryu & K. V. Shenoy (2012) Neural population dynamics during reaching. *Nature,* 487**,** 51-6.

Collinger, J. L., S. Foldes, T. M. Bruns, B. Wodlinger, R. Gaunt & D. J. Weber (2013) Neuroprosthetic technology for individuals with spinal cord injury. *J Spinal Cord Med,* 36**,** 258-72.

Degenhart, A. D., J. Eles, R. Dum, J. L. Mischel, I. Smalianchuk, B. Endler, R. C. Ashmore, E. C. Tyler-Kabara, N. G. Hatsopoulos, W. Wang, A. P. Batista & X. T. Cui (2016) Histological evaluation of a chronically-implanted electrocorticographic electrode grid in a non-human primate. *J Neural Eng,* 13**,** 046019.

Duraivel, Suseendrakumar, Shervin Rahimpour, Chia-Han Chiang, Michael Trumpis, Charles Wang, Katrina Barth, Shivanand P. Lad, Allan H. Friedman, Derek G. Southwell, Saurabh R. Sinha, Jonathan Viventi, and Gregory Cogan. 2022. High-Resolution Neural Recordings Improve the Accuracy of Speech Decoding. preprint. Neuroscience. doi: 10.1101/2022.05.19.492723.

Ganguly, K. & J. M. Carmena (2009) Emergence of a stable cortical map for neuroprosthetic control. *PLoS Biol,* 7**,** e1000153.

Ganguly, K., D. F. Dimitrov, J. D. Wallis & J. M. Carmena (2011) Reversible large-scale modification of cortical networks during neuroprosthetic control. *Nat Neurosci,* 14**,** 662-7.

Ganguly, K., L. Secundo, G. Ranade, A. Orsborn, E. F. Chang, D. F. Dimitrov, J. D. Wallis, N. M. Barbaro, R. T. Knight & J. M. Carmena (2009) Cortical representation of ipsilateral arm movements in monkey and man. *J Neurosci,* 29**,** 12948-56.

Gilja, V., C. A. Chestek, I. Diester, J. M. Henderson, K. Deisseroth & K. V. Shenoy (2011) Challenges and opportunities for next-generation intracortically based neural prostheses. *IEEE Trans Biomed Eng,* 58**,** 1891-9.

Gilja, V., P. Nuyujukian, C. A. Chestek, J. P. Cunningham, M. Y. Byron, J. M. Fan, M. M. Churchland, M. T. Kaufman, J. C. Kao & S. I. Ryu (2012) A high-performance neural prosthesis enabled by control algorithm design. *Nature neuroscience,* 15**,** 1752-1757.

Hochberg, L. R., D. Bacher, B. Jarosiewicz, N. Y. Masse, J. D. Simeral, J. Vogel, S. Haddadin, J. Liu, S. S. Cash & P. van der Smagt (2012a) Reach and grasp by people with tetraplegia using a neurally controlled robotic arm. *Nature,* 485**,** 372-375.

Hochberg, L. R., D. Bacher, B. Jarosiewicz, N. Y. Masse, J. D. Simeral, J. Vogel, S. Haddadin, J. Liu, S. S. Cash, P. van der Smagt & J. P. Donoghue (2012b) Reach and grasp by people with tetraplegia using a neurally controlled robotic arm. *Nature,* 485**,** 372-5.

Hochberg, L. R., M. D. Serruya, G. M. Friehs, J. A. Mukand, M. Saleh, A. H. Caplan, A. Branner, D. Chen, R. D. Penn & J. P. Donoghue (2006) Neuronal ensemble control of prosthetic devices by a human with tetraplegia. *Nature,* 442**,** 164-71.

Homer, M. L., A. V. Nurmikko, J. P. Donoghue & L. R. Hochberg (2013) Sensors and decoding for intracortical brain computer interfaces. *Annu Rev Biomed Eng,* 15**,** 383-405.

Huggins, J. E., P. A. Wren & K. L. Gruis (2011) What would brain-computer interface users want? Opinions and priorities of potential users with amyotrophic lateral sclerosis. *Amyotroph Lateral Scler,* 12**,** 318-24.

Kennedy, P. R. (1994) 'Locked-in' patients. *Neurology,* 44**,** 366-7.

Kennedy, P. R. & R. A. Bakay (1998) Restoration of neural output from a paralyzed patient by a direct brain connection. *Neuroreport,* 9**,** 1707-11.

Kim, S. P., J. D. Simeral, L. R. Hochberg, J. P. Donoghue & M. J. Black (2008a) Neural control of computer cursor velocity by decoding motor cortical spiking activity in humans with tetraplegia. *Journal of Neural Engineering,* 5**,** 455-76.

--- (2008b) Neural control of computer cursor velocity by decoding motor cortical spiking activity in humans with tetraplegia. *J Neural Eng,* 5**,** 455-76.

Kubler, A., B. Kotchoubey, J. Kaiser, J. R. Wolpaw & N. Birbaumer (2001) Brain-computer communication: unlocking the locked in. *Psychol Bull,* 127**,** 358-75.

Leuthardt, E. C., K. J. Miller, G. Schalk, R. P. Rao & J. G. Ojemann (2006) Electrocorticography-based brain computer interface--the Seattle experience. *IEEE Trans Neural Syst Rehabil Eng,* 14**,** 194-8.

Leuthardt, E. C., G. Schalk, J. Roland, A. Rouse & D. W. Moran (2009) Evolution of brain-computer interfaces: going beyond classic motor physiology. *Neurosurg Focus,* 27**,** E4.

Leuthardt, E. C., G. Schalk, J. R. Wolpaw, J. G. Ojemann & D. W. Moran (2004) A brain-computer interface using electrocorticographic signals in humans. *J Neural Eng,* 1**,** 63-71.

Matthew, R. P., E. J. Mica, W. Meinhold, J. A. Loeza, M. Tomizuka & R. Bajcsy (2015) Initial investigation into the effect of an Active/Passive exoskeleton on hammer curl performance in healthy subjects. *Conf Proc IEEE Eng Med Biol Soc,* 2015**,** 3607-10.

Matthew, R. P., V. Shia, G. Venture & R. Bajcsy (2016) Generating physically realistic kinematic and dynamic models from small data sets: An application for sit-to-stand actions. *Conf Proc IEEE Eng Med Biol Soc,* 2016**,** 2173-2178.

Metzger, Sean L., Kaylo T. Littlejohn, Alexander B. Silva, David A. Moses, Margaret P. Seaton, Ran Wang, Maximilian E. Dougherty, Jessie R. Liu, Peter Wu, Michael A. Berger, Inga Zhuravleva, Adelyn Tu-Chan, Karunesh Ganguly, Gopala K. Anumanchipalli, and Edward F. Chang. 2023. “A High-Performance Neuroprosthesis for Speech Decoding and Avatar Control.” Nature. doi: 10.1038/s41586-023-06443-4.

Miller, K. J., D. Hermes, C. J. Honey, M. Sharma, R. P. Rao, M. den Nijs, E. E. Fetz, T. J. Sejnowski, A. O. Hebb, J. G. Ojemann, S. Makeig & E. C. Leuthardt (2010) Dynamic modulation of local population activity by rhythm phase in human occipital cortex during a visual search task. *Front Hum Neurosci,* 4**,** 197.

Monti, M. M., A. Vanhaudenhuyse, M. R. Coleman, M. Boly, J. D. Pickard, L. Tshibanda, A. M. Owen & S. Laureys (2010) Willful modulation of brain activity in disorders of consciousness. *N Engl J Med,* 362**,** 579-89.

Morrell, M. J. & R. N. S. S. i. E. S. Group (2011) Responsive cortical stimulation for the treatment of medically intractable partial epilepsy. *Neurology,* 77**,** 1295-304.

Moses, D.A., Metzger, S.L., Liu, J.R., et al., (2021) Neuroprosthesis for Decoding Speech in a Paralyzed Person with Anarthria. *N Engl J Med,* 385, 217-227.

Muller, Leah, Sarah Felix, Kedar G. Shah, Kye Lee, Satinderpall Pannu, and Edward F. Chang. 2016. “Thin-Film, High-Density Micro-Electrocorticographic Decoding of a Human Cortical Gyrus.” Pp. 1528–31 in 2016 38th Annual International Conference of the IEEE Engineering in Medicine and Biology Society (EMBC). Orlando, FL, USA: IEEE.

Nadkarni, P. M., L. Ohno-Machado & W. W. Chapman (2011a) Natural language processing: an introduction. *J Am Med Inform Assoc,* 18**,** 544-51.

Nadkarni, P. M., L. Ohno-Machado & W. W. Chapman (2011b) Natural language processing: an introduction. *Journal of the American Medical Informatics Association,* 18**,** 544-551.

Nicolelis, M. A. & M. A. Lebedev (2009) Principles of neural ensemble physiology underlying the operation of brain-machine interfaces. *Nat Rev Neurosci,* 10**,** 530-40.

Nuyujukian, D. S., J. Voutsinas, L. Bernstein & S. S. Wang (2014a) Medication use and multiple myeloma risk in Los Angeles County. *Cancer Causes Control,* 25**,** 1233-7.

Nuyujukian, P., J. C. Kao, J. M. Fan, S. D. Stavisky, S. I. Ryu & K. V. Shenoy (2014b) Performance sustaining intracortical neural prostheses. *Journal of neural engineering,* 11**,** 066003.

Oskarsson, B., N. C. Joyce, E. De Bie, A. Nicorici, R. Bajcsy, G. Kurillo & J. J. Han (2016) Upper extremity 3-dimensional reachable workspace assessment in amyotrophic lateral sclerosis by Kinect sensor. *Muscle Nerve,* 53**,** 234-41.

Pandarinath, C., V. Gilja, C. H. Blabe, P. Nuyujukian, A. A. Sarma, B. L. Sorice, E. N. Eskandar, L. R. Hochberg, J. M. Henderson & K. V. Shenoy (2015) Neural population dynamics in human motor cortex during movements in people with ALS. *Elife,* 4**,** e07436.

Pasley, B. N., S. V. David, N. Mesgarani, A. Flinker, S. A. Shamma, N. E. Crone, R. T. Knight & E. F. Chang (2012) Reconstructing speech from human auditory cortex. *Plos Biology,* 10**,** e1001251.

Schalk, G., K. J. Miller, N. R. Anderson, J. A. Wilson, M. D. Smyth, J. G. Ojemann, D. W. Moran, J. R. Wolpaw & E. C. Leuthardt (2008) Two-dimensional movement control using electrocorticographic signals in humans. *J Neural Eng,* 5**,** 75-84.

Schwartz, A. B. (2004) Cortical neural prosthetics. *Annu Rev Neurosci,* 27**,** 487-507.

Schwartz, A. B., X. T. Cui, D. J. Weber & D. W. Moran (2006) Brain-controlled interfaces: movement restoration with neural prosthetics. *Neuron,* 52**,** 205-20.

Selzer, M. E., S. Clarke, L. G. Cohen, G. Kwakkel & R. H. Miller. 2014. *Textbook of neural repair and rehabilitation*. Cambridge: Cambridge University Press.

Shanechi, M. M., A. L. Orsborn & J. M. Carmena (2016) Robust Brain-Machine Interface Design Using Optimal Feedback Control Modeling and Adaptive Point Process Filtering. *PLoS Comput Biol,* 12**,** e1004730.

Shenoy, K. V. & J. M. Carmena (2014) Combining decoder design and neural adaptation in brain-machine interfaces. *Neuron,* 84**,** 665-80.

Simeral, J. D., S. P. Kim, M. J. Black, J. P. Donoghue & L. R. Hochberg (2011) Neural control of cursor trajectory and click by a human with tetraplegia 1000 days after implant of an intracortical microelectrode array. *J Neural Eng,* 8**,** 025027.

Slutzky, M. W., L. R. Jordan, E. W. Lindberg, K. E. Lindsay & L. E. Miller (2011) Decoding the rat forelimb movement direction from epidural and intracortical field potentials. *J Neural Eng,* 8**,** 036013.

Spataro, R., M. Ciriacono, C. Manno & V. La Bella (2014) The eye-tracking computer device for communication in amyotrophic lateral sclerosis. *Acta Neurol Scand,* 130**,** 40-5.

Taylor, D. M., S. I. Tillery & A. B. Schwartz (2002) Direct cortical control of 3D neuroprosthetic devices. *Science,* 296**,** 1829-32.

Thompson, D. E., L. R. Quitadamo, L. Mainardi, S. Gao, P.-J. Kindermans, J. D. Simeral, R. Fazel-Rezai, M. Matteucci, T. H. Falk & L. Bianchi (2014a) Performance measurement for brain–computer or brain–machine interfaces: a tutorial. *Journal of neural engineering,* 11**,** 035001.

Thompson, D. E., L. R. Quitadamo, L. Mainardi, K. U. Laghari, S. Gao, P. J. Kindermans, J. D. Simeral, R. Fazel-Rezai, M. Matteucci, T. H. Falk, L. Bianchi, C. A. Chestek & J. E. Huggins (2014b) Performance measurement for brain-computer or brain-machine interfaces: a tutorial. *J Neural*

Eng, 11**,** 035001.
Tort, A. B., R. Komorowski, H. Eichenbaum & N. Kopell (2010) Measuring phase-amplitude coupling between neuronal oscillations of different frequencies. *J Neurophysiol,* 104**,** 1195-210.
Wolpaw, J. R., N. Birbaumer, D. J. McFarland, G. Pfurtscheller & T. M. Vaughan (2002) Brain-computer interfaces for communication and control. *Clin Neurophysiol,* 113**,** 767-91.
