# Supplementary material for: Simultaneous speech and gesture decoding for multimodal communication in paralysis
Source: Nat Neurosci. 2026 Sep 14;29(10):2445–55. doi: 10.1038/s41593-026-02446-2 (PMC13633112; doi:10.1038/s41593-026-02446-2)
Supplement: Supplementary file 2 [file 41593_2026_2446_MOESM2_ESM.pdf]

nature portfolio

Corresponding author(s): Edward F. Chang

Last updated by author(s): Aug 4, 2026

Reporting Summary

Nature Portfolio wishes to improve the reproducibility of the work that we publish. This form provides structure for consistency and transparency in reporting. For further information on Nature Portfolio policies, see our Editorial Policies and the Editorial Policy Checklist.

Statistics

For all statistical analyses, confirm that the following items are present in the figure legend, table legend, main text, or Methods section.

n/a	Confirmed	
☐	☒	The exact sample size (*n*) for each experimental group/condition, given as a discrete number and unit of measurement
☐	☒	A statement on whether measurements were taken from distinct samples or whether the same sample was measured repeatedly
☐	☒	The statistical test(s) used AND whether they are one- or two-sided<br>*Only common tests should be described solely by name; describe more complex techniques in the Methods section.*
☒	☐	A description of all covariates tested
☐	☒	A description of any assumptions or corrections, such as tests of normality and adjustment for multiple comparisons
☐	☒	A full description of the statistical parameters including central tendency (e.g. means) or other basic estimates (e.g. regression coefficient) AND variation (e.g. standard deviation) or associated estimates of uncertainty (e.g. confidence intervals)
☐	☒	For null hypothesis testing, the test statistic (e.g. *F*, *t*, *r*) with confidence intervals, effect sizes, degrees of freedom and *P* value noted<br>*Give P values as exact values whenever suitable.*
☒	☐	For Bayesian analysis, information on the choice of priors and Markov chain Monte Carlo settings
☒	☐	For hierarchical and complex designs, identification of the appropriate level for tests and full reporting of outcomes
☐	☒	Estimates of effect sizes (e.g. Cohen's *d*, Pearson's *r*), indicating how they were calculated

Our web collection on statistics for biologists contains articles on many of the points above.

Software and code

Policy information about availability of computer code

Data collection	Open source: Python 3.8<br>Commercial software: We used software from Blackrock Microsystems to acquire and perform basic preprocessing on data from the neural implant.<br>Custom code: We used a real-time software package (referred to as rtNSR; described in prior literature that is referenced in the current manuscript) to further process neural data in real time.
Data analysis	Open source: Python 3.8<br>Custom code: We used custom scripts and modules to analyze the neural data. The accompanying manuscript code will be publicly available in the GitHub repository associated with this paper at https://github.com/ChangLabUcsf/Brosler2026_simultaneous_decoding.git upon publication, but the authors still reserve all rights to the code.

For manuscripts utilizing custom algorithms or software that are central to the research but not yet described in published literature, software must be made available to editors and reviewers. We strongly encourage code deposition in a community repository (e.g. GitHub). See the Nature Portfolio guidelines for submitting code & software for further information.

Data

Policy information about availability of data

All manuscripts must include a data availability statement. This statement should provide the following information, where applicable:

- Accession codes, unique identifiers, or web links for publicly available datasets
- A description of any restrictions on data availability
- For clinical datasets or third party data, please ensure that the statement adheres to our policy

ECoG neural data relevant to this study is under restricted access according to our clinical trial protocol. The protocol allows us to share de-identified data with researchers from other institutions upon reasonable request but prohibits us from making it publicly available. All ECoG neural data is available in a restricted-access Zenodo repository at 10.5281/zenodo.21829509. Requests for access should be directed to E.F.C. at edward.chang@ucsf.edu and will typically be reviewed within three weeks. Approved recipients must maintain confidentiality of the data and may not redistribute without prior authorization from E.F.C. The clinical trial protocol is included as Supplementary Information.

Research involving human participants, their data, or biological material

Policy information about studies with human participants or human data. See also policy information about sex, gender (identity/presentation), and sexual orientation and race, ethnicity and racism.

Reporting on sex and gender

Participants' gender was recorded as part of clinical-trial participation and is reported using participant pronouns. The study included two male participants and one female participant. Sex- and gender-based analyses were not performed because this was a feasibility study involving three individuals with severe speech and motor impairments, and the study was not designed or powered to assess effects of sex or gender.

Reporting on race, ethnicity, or other socially relevant groupings

Race, ethnicity and other socially relevant groupings were not used as experimental variables and were not included in the analyses. The study was a small feasibility study of ECoG BCI control in three participants with severe speech and motor impairments and was not designed or powered to assess differences across socially relevant groups.

Population characteristics

Three participants were recruited and enrolled through the BCI Restoration of Arm and Voice clinical trial between November 2018 and March 2025. Participants enrolled at ages 36, 47 and 60. All participants had intact cognitive function and severe speech and motor impairments. Bravo-1r had severe spastic quadriparesis and anarthria due to a bilateral pontine stroke at age 20; Bravo-3 had quadriplegia and anarthria after a pontine stroke at age 30; and Bravo-6 had amyotrophic lateral sclerosis with progressive vocal-tract paralysis and loss of intelligible speech. All participants used augmentative or alternative communication methods in daily life. Bravo-1r's native language is Spanish, Bravo-3's native language is English, and Bravo-6's native language is German; all participants are fluent in English.

Recruitment

Participants were recruited through the BCI Restoration of Arm and Voice clinical trial from clinics specializing in the treatment of stroke, ALS, and general neurological disorders, at UCSF and the San Francisco VA Medical Center. Prior to enrollment into the study, an informal phone interview to schedule an office-based evaluation takes place, followed by three outpatient screening visits. During the first outpatient visit, we describe the trial in detail and answer all questions. Should the participant choose to continue, we schedule another visit to conduct a physical exam and to perform screening to determine eligibility. An MRI and CT of the brain is obtained for future surgical planning and to determine further eligibility. Additionally, an ECG and chest x-ray are also obtained. We schedule a third follow-up visit to review this data and answer remaining questions prior to enrollment in the trial.

Eligible participants had severe speech and motor impairments and were enrolled after providing informed consent. Because this was an early feasibility clinical trial requiring neurosurgical implantation and repeated data-collection sessions, the cohort was small and may reflect self-selection of individuals willing and able to participate in an implanted BCI study. These factors do not affect the within-participant decoding analyses that were the primary focus of the study.

Ethics oversight

The U.S. Food and Drug Administration approved the investigational use of the neural implant device. The study protocol was approved by the University of California, San Francisco Institutional Review Board and the National Institute on Deafness and Other Communication Disorders, National Institutes of Health. All participants provided informed consent for participation and for the release of recorded videos.

Note that full information on the approval of the study protocol must also be provided in the manuscript.

Field-specific reporting

Please select the one below that is the best fit for your research. If you are not sure, read the appropriate sections before making your selection.

☒ Life sciences ☐ Behavioural & social sciences ☐ Ecological, evolutionary & environmental sciences

For a reference copy of the document with all sections, see nature.com/documents/nr-reporting-summary-flat.pdf

Life sciences study design

All studies must disclose on these points even when the disclosure is negative.

Sample size	Sample sizes for tests were determined by the amount of available data with the participant. The amount of data collected with the participant was dependent on our estimation of how much data (e.g. how many trials of the tasks) would be required to reasonably estimate the measurements of interest.
Data exclusions	No trials were excluded from model training or decoding analyses. For visualization of high-gamma activity maps on the cortical surface, trials were excluded only if they contained clear neural signal artifacts that produced maximum high-gamma activity values outside the physiologically plausible range. The exclusion threshold was determined by manual inspection of the high-gamma activity distributions.
Replication	To verify reproducibility of the experimental findings, we evaluated decoding performance across multiple recording blocks, task contexts, and participants. During real-time evaluation of the simultaneous speech-and-gesture decoding system in participant Bravo-6, we collected 200 trials across 12 recording blocks and observed consistent performance across blocks, as described in the manuscript. We further tested the system in a conversational setting, in which the participant responded to prompts spoken by the experimenter across five example conversation blocks, and observed similarly high performance. To assess reproducibility across participants, we also evaluated the real-time conversational decoding task in a second participant with a different disease etiology: Bravo-6 had ALS, whereas Bravo-1r had a brainstem stroke. This second participant also showed high real-time decoding performance. In both participants, we additionally performed offline analyses using 10-fold cross-validation to assess decoding accuracy across held-out trials.
Randomization	Random allocation of participants into experimental groups was not applicable because this study did not involve assignment to treatment or control groups. Neural activity and decoding performance were analyzed separately within each participant. For model evaluation, trials were assigned to cross-validation folds using a stratified randomization procedure to ensure that each fold contained a balanced distribution of target labels, reducing potential effects of class imbalance on decoding accuracy.
Blinding	Blinding was not applicable because this study did not involve treatment or control group assignment. The goal was to evaluate the feasibility and performance of a real-time multi-effector speech-and-gesture decoding system controlled by neural activity from participants with paralysis.

Reporting for specific materials, systems and methods

We require information from authors about some types of materials, experimental systems and methods used in many studies. Here, indicate whether each material, system or method listed is relevant to your study. If you are not sure if a list item applies to your research, read the appropriate section before selecting a response.

Materials & experimental systems

n/a	Involved in the study
☒	☐ Antibodies
☒	☐ Eukaryotic cell lines
☒	☐ Palaeontology and archaeology
☒	☐ Animals and other organisms
☐	☒ Clinical data
☒	☐ Dual use research of concern
☒	☐ Plants

Methods

n/a	Involved in the study
☒	☐ ChIP-seq
☒	☐ Flow cytometry
☒	☐ MRI-based neuroimaging

Clinical data

Policy information about clinical studies

All manuscripts should comply with the ICMJE guidelines for publication of clinical research and a completed CONSORT checklist must be included with all submissions.

Clinical trial registration	Clinicaltrials.gov NCT03698149
Study protocol	A description of the study is available on ClinicalTrials.gov under identifier NCT03698149 (https://clinicaltrials.gov/ct2/show/NCT03698149). The full clinical-trial protocol is available as a supplementary file to this paper.
Data collection	Data was collected either in participants' homes or in a nearby office space. Participants were enrolled as part of the BCI Restoration of Arm and Voice clinical trial. Data collection for the present study occurred between September 2022 and January 2024 for Bravo-3, May 2024 and December 2024 for Bravo-1r, and June 2025 and November 2025 for Bravo-6.
Outcomes	This clinical trial is a phase I, single-center early-feasibility study designed to evaluate the long-term potential of ECoG-based neural interfaces to control advanced neuroprostheses for motor and communication restoration. The primary outcomes were predefined in the clinical trial protocol and assess the safety and feasibility of the investigational device. Safety is assessed through the incidence and evaluation of treatment-emergent adverse events, and feasibility is assessed through participants' ability to control motor and communication interfaces. Because this is a low participant enrollment, exploratory clinical trial, secondary outcomes were not predefined. A variety of participant-specific tasks and analysis methods are therefore explored throughout the trial to assess device

performance and guide future research. The decoder-performance measures reported in this manuscript are exploratory and were not predefined clinical trial outcomes.

Plants

Seed stocks

Report on the source of all seed stocks or other plant material used. If applicable, state the seed stock centre and catalogue number. If plant specimens were collected from the field, describe the collection location, date and sampling procedures.

Novel plant genotypes

Describe the methods by which all novel plant genotypes were produced. This includes those generated by transgenic approaches, gene editing, chemical/radiation-based mutagenesis and hybridization. For transgenic lines, describe the transformation method, the number of independent lines analyzed and the generation upon which experiments were performed. For gene-edited lines, describe the editor used, the endogenous sequence targeted for editing, the targeting guide RNA sequence (if applicable) and how the editor was applied.

Authentication

Describe any authentication procedures for each seed stock used or novel genotype generated. Describe any experiments used to assess the effect of a mutation and, where applicable, how potential secondary effects (e.g. second site T-DNA insertions, mosiacism, off-target gene editing) were examined.
